# How adverse childhood experiences get under the skin: A systematic review, integration and methodological discussion on threat and reward learning mechanisms

**DOI:** 10.7554/eLife.92700

**Published:** 2024-07-16

**Authors:** Julia Ruge, Mana R Ehlers, Alexandros Kastrinogiannis, Maren Klingelhöfer-Jens, Alina Koppold, Rany Abend, Tina B Lonsdorf

**Affiliations:** 1 University Medical Center Hamburg-Eppendorf, Institute for Systems Neuroscience Hamburg Germany; 2 University of Bielefeld Bielefeld Germany; 3 Department of Neurology, Max Planck Institute for Human Cognitive and Brain Sciences Leipzig Germany; 4 Reichman University Herzliya Israel; Trinity College Dublin Ireland; University of California, Los Angeles United States

**Keywords:** fear conditioning, reward learning, adverse childhood experiences, systematic literature review

## Abstract

Adverse childhood experiences (ACEs) are a major risk factor for the development of multiple psychopathological conditions, but the mechanisms underlying this link are poorly understood. Associative learning encompasses key mechanisms through which individuals learn to link important environmental inputs to emotional and behavioral responses. ACEs may impact the normative maturation of associative learning processes, resulting in their enduring maladaptive expression manifesting in psychopathology. In this review, we lay out a systematic and methodological overview and integration of the available evidence of the proposed association between ACEs and threat and reward learning processes. We summarize results from a systematic literature search (following PRISMA guidelines) which yielded a total of 81 articles (threat: n=38, reward: n=43). Across the threat and reward learning fields, behaviorally, we observed a converging pattern of aberrant learning in individuals with a history of ACEs, independent of other sample characteristics, specific ACE types, and outcome measures. Specifically, blunted threat learning was reflected in reduced discrimination between threat and safety cues, primarily driven by diminished responding to conditioned threat cues. Furthermore, attenuated reward learning manifested in reduced accuracy and learning rate in tasks involving acquisition of reward contingencies. Importantly, this pattern emerged despite substantial heterogeneity in ACE assessment and operationalization across both fields. We conclude that blunted threat and reward learning may represent a mechanistic route by which ACEs may become physiologically and neurobiologically embedded and ultimately confer greater risk for psychopathology. In closing, we discuss potentially fruitful future directions for the research field, including methodological and ACE assessment considerations.

## Introduction

Adverse childhood experiences (ACEs) are defined as ‘experiences that are likely to require significant adaptation by an average child and that represent a deviation from the expectable environment’ (cf. McLaughlin, 2016), which in turn impacts a typical (neuro-) developmental trajectory. This definition of ACEs covers a wide range of adverse experiences, such as sexual and physical abuse, emotional abuse and/or neglect, physical neglect, witnessing domestic violence, peer victimization and institutional rearing (e.g. Anda et al., 2006; McLaughlin et al., 2016). For a recent overview of the assessment and operationalization of ACEs in the literature, see Koppold et al., 2023. ACEs confer a heightened risk for developing severe and enduring behavioral, somatic and psychopathological conditions (Felitti, 2002; Gilbert et al., 2009; Green et al., 2010; Heim and Nemeroff, 2001; McLaughlin et al., 2012; Moffitt et al., 2007; Teicher et al., 2022), which incur not only substantial individual suffering but also significant societal costs (Hughes et al., 2021), and are associated with considerable mortality and morbidity. As approximately 60% of all children and adolescents are exposed to at least one adverse event (Madigan et al., 2023), understanding the mechanisms altered by ACEs is crucial for developing theory-guided prevention and intervention approaches (e.g., McLaughlin et al., 2019; Romeo et al., 2018). Early work on ACEs typically focused on single adversity types, such as neglect (Crouch and Milner, 1993) or sexual abuse (Heim et al., 2013) or considered the total number of events experienced as cumulative risk (e.g. *allostatic load hypothesis*, McEwen, 2003). Over time, the latter approach has been criticized, as different types of experiences are simply ‘lumped’ into a single general adversity category, implicitly assuming a common and equally powerful, additive impact of different ACE types (Smith and Pollak, 2021). In contrast, the *specificity model* (e.g. McMahon et al., 2003; Pollak et al., 2000; Pollak and Tolley-Schell, 2004) posits that different types of adverse events impact distinct mechanisms, leading to distinct negative outcomes. Alongside distinct adversity types, research has begun to examine the impact of ACE characteristics on developmental trajectories and outcomes, such as chronicity and intensity, environmental context, or developmental timing of exposure (for a detailed overview see Smith and Pollak, 2021).

Recently, the *dimensional model of adversity and psychopathology* (DMAP) has emerged as a model of ACE that extends the cumulative and specificity approaches by specifically implicating heightened environmental threat and deprivation as two primary adverse experience dimensions that impact distinct neurobiological systems and lead to differential clinical outcomes (Berens et al., 2017; Kuhlman et al., 2017; Machlin et al., 2019; McLaughlin et al., 2016; McLaughlin et al., 2014; Sheridan and McLaughlin, 2016; Sheridan and McLaughlin, 2014; Smith and Pollak, 2021; for an extension see Ellis et al., 2022). In the DMAP, threat has been conceptualized as ‘the presence of an atypical (i.e. unexpected) experience characterized by actual or threatened death, injury, sexual violation, or harm to one’s physical integrity’, while deprivation has been conceptualized as ‘the absence of expected environmental inputs in cognitive (e.g. language) and social domains, as well as the absence of species- and age-typical complexity in environmental stimulation’ (cf. Sheridan and McLaughlin, 2014). Accordingly, DMAP suggests a specific impact of threat-related experiences on emotional functioning whereas deprivation experiences are hypothesized to affect cognitive functioning (Sheridan and McLaughlin, 2014). A conceptually appealing advantage of DMAP is that threat and deprivation are considered distinct adversity dimensions with specific and distinct effects on developmental mechanisms that can supposedly be examined in parallel. Although there is still an ongoing scientific debate in which the DMAP has been discussed controversially (McLaughlin et al., 2021; Pollak and Smith, 2021; Smith and Pollak, 2021), a common emphasis placed by all models is on the impact of adversity on the development of associative learning processes in mediating the enduring effects of ACE on later functioning (McLaughlin, 2016; McLaughlin et al., 2019). Associative learning describes implicit learning processes through which environmental cues gain predictive value of positive or negative outcomes. Specifically, threat learning entails an initially neutral stimulus becoming associated with an aversive outcome, while reward learning involves environmental cues becoming predictive of a positive outcome. Being able to identify environmental threats and associated cues and rapidly mobilize adequate defensive responses is essential to ensure survival of the organism. Likewise, interpreting the predictive value of environmental cues or actions that are associated with or are reinforced by rewards is central to guiding motivated behavior and decision-making (Cisek, 2019; Dranias et al., 2008; LeDoux, 2012).

Crucially, a considerable amount of cross-species research suggests that the neurobiological circuitry contributing to threat and reward learning is particularly malleable and undergoes substantial maturation and shaping during childhood and adolescence (McLaughlin et al., 2014; Somerville et al., 2010; Tottenham and Galván, 2016). Given this developmental vulnerability, ACEs have been suggested to exert their enduring deleterious effects by impacting the normative development of the circuitry underlying implicit threat and reward learning (Oltean et al., 2023), which is thought to represent an adaptation to unpredictable or fast changing environments (McLaughlin et al., 2019; McLaughlin et al., 2014). For example, growing up in a household with a high probability of physical violence may impact patterns of threat learning: Growing up in a volatile and uncertain environment where incidences of threatening events cannot be predicted and seemingly occur at random disrupt associative learning processes in the long term. Enduring disruptions in associative learning processes may then manifest as persistently maladaptive emotional, cognitive, and behavioral responses, and thereby constitute an important mechanism underlying the onset and persistence of psychopathology (McLaughlin et al., 2019; McLaughlin and Sheridan, 2016). Aberrant threat learning manifesting in reduced discrimination between threat and safety signals has consequences for survival of the organism and the efficient allocation of energy. Furthermore, while the generalization of fear responses to similar cues is adaptive to ensure survival in the potential presence of threat (‘better safe than sorry’), excessive or context-inappropriate overgeneralization can be very costly for the organism and depriving it from benefits (Duits et al., 2015; McCrory et al., 2017). However, not only threat learning but also reward learning is central to an organism’s adaptive functioning. For instance, aberrant patterns of reward learning such as reduced reward anticipation and reduced sensitivity to rewarding feedback, are characteristic of anhedonia-like symptoms typical for mood disorders (Gerin et al., 2017; Oltean et al., 2023). Of note, also other mechanisms, that are, however, not the focus of this work, are of potential relevance including emotion regulation and executive control (McCrory et al., 2017) as well as the quality of the social network (McCrory et al., 2022). Thus, early adverse experiences have enduring consequences due to the heightened neural plasticity during development (Kolb and Gibb, 2014) and are considered to convey an adaptation to the environment in which the child develops (i.e. experience-dependent plasticity, McCrory et al., 2017).

In order to paint a coherent picture of the influence of ACEs on associative learning processes, it is essential to provide a systematic and methodological investigation and integration of the relatively insulated fields of threat and reward learning. Improving our understanding of how reward and threat learning may be impacted by ACEs could inform the mechanistic link between childhood adversity and psychopathology. Further, it holds promise to improve existing and develop novel avenues in targeted prevention and intervention approaches for psychopathology associated with a history of ACEs (McLaughlin et al., 2019; Odriozola and Gee, 2021). In addition, this systematic investigation serves to identify key methodological considerations and challenges in the study of ACEs and can be expected to spark discussion for future research.

## Methods

The systematic literature search was conducted according to the PRISMA guidelines (Moher et al., 2015; Page et al., 2021). Studies published before December 2022 were included if tasks involved a threat or reward learning element (instructed or uninstructed) and investigated associations with ACEs using terms related to ‘fear or threat conditioning’, ‘aversive anticipation’, ‘threat of shock’ in the threat learning field and ‘reward or reinforcement learning or anticipation’ in the reward learning field, as well as the terms ‘adversity’, ‘maltreatment’, ‘abuse’, ‘neglect’, ‘stress’, ‘trauma’, ‘deprivation’, ‘institutionalization’, ‘orphanage’, ‘adoption’, ‘harassment’, ‘bullying’, ‘household violence’, ‘domestic violence’, ‘poverty’, ‘low SES’, ‘food insecurity’ and ‘adverse childhood experiences’ in ‘children’, ‘childhood’, ‘early’, ‘youth’ or ‘adolescents’. Articles were excluded, if they were reviews or meeting abstracts, or if they used non-human samples, if they did not include a learning element or if they assessed general life-time trauma or adversity instead of ACEs (see Supplementary file 1 for details).

A threat or reward learning element was defined as physiological or behavioral adaptation over time to the repetitive or prolonged presentation of a cue. While fear conditioning and other reinforcement learning tasks can clearly be categorized as learning tasks, the view on the monetary incentive delay (MID) task is more nuanced. The MID can also be understood as a learning task because participants show a response modulation by the reward amount (Dhingra et al., 2020). More precisely, the task induces changes in sensory processing over time (Krugliakova et al., 2019), providing evidence of instrumental or reinforcement learning, where the presentation of a specific cue triggers an action that is rewarded. Likewise, the learned, anticipatory response (Wilson et al., 2018; as assessed through BOLD fMRI) to a reward is conceptually comparable to BOLD fMRI responses during presentation of CS+ vs. CS- in threat learning (see Box 1 for information on the paradigm), that is the anticipation of threat vs. safety.

Box 1. **Paradigms employed to study threat – and reward-related learning processes.**

**Threat learning paradigms**

**Fear conditioning paradigms** (Marusak et al., 2021; Jovanovic et al., 2022; Qiu et al., 2023; France et al., 2022; McLaughlin, 2016; Jenness et al., 2019; Susman et al., 2021; Lange et al., 2019; Zoladz et al., 2022; Scharfenort and Lonsdorf, 2016a; Lis et al., 2020; Thome et al., 2018; Bremner et al., 2005; Kuehl et al., 2020; Morrison et al., 2022; Klingelhöfer-Jens et al., 2023; Jovanovic et al., 2020; Stenson et al., 2021; Rowland et al., 2022; Estrada et al., 2020; Morey et al., 2015; Deslauriers et al., 2018; Harnett et al., 2019).

The Fear Conditioning paradigm consists of a number of successive experimental phases. During fear acquisition training, an initially neutral stimulus (i.e. conditioned stimulus, CS+) is repeatedly paired with an unconditioned aversive stimulus (US) and as a consequence the CS+ acquires the ability to elicit conditioned responses (CRs). Typically, a second stimulus (i.e. CS-) is never paired with the US and hence serves as a control stimulus which has also been suggested to signal safety. Following fear acquisition a generalization phase can take place, during which the potential generalization of CRs to new stimuli, perceptually resembling the CSs (generalization stimuli, GS) can be observed. In a subsequent (optional) extinction training phase, both CSs are presented without the US, which leads to a gradual waning of the CRs (Milad and Quirk, 2012; Vervliet et al., 2013). While fear acquisition training serves as a laboratory model for the acquisition of fear, extinction serves as a model for the active ingredient of exposure based treatment. Relapse phenomena have been modeled experimentally in return of fear phases such as renewal (Vervliet et al., 2013) or reinstatement (Haaker et al., 2014).

**Fear conditioning paradigms in pediatric samples** (Machlin et al., 2019; Milojevich et al., 2020; Silvers et al., 2016; DeCross et al., 2022)

Typical fear conditioning paradigm has also been modified in different ways to be applicable in samples of (young) children and hence differ partly substantially from those employed in adults (see Shechner et al., 2014 for recommendations). The paradigms specifically tailored to children samples often rely on a block design with blocks of reinforced and unreinforced CS+ as well as blocks of CS- trials (Machlin et al., 2019; Milojevich et al., 2020; Silvers et al., 2016), following the reasoning that this facilitates learning in young cohorts. Yet, these paradigms have also been applied in older children and adolescents and also non-block designs for fear acquisition and generalization have been successfully applied in samples of children (Qiu et al., 2023; Schiele et al., 2016). Typically the use of aversive electrotactile stimulation is precluded for ethical reasons and hence aversive tones, human screams or airblasts delivered at the larynx are typically used.

**AX+BX- task** (Jovanovic et al., 2009; Huskey et al., 2022)

The AX+ BX- task, also referred to as the conditional discrimination paradigm, is a variant of the fear conditioning paradigm used to study fear inhibition (Jovanovic et al., 2005; Myers and Davis, 2004). It was designed to allow for the systematic comparison of excitatory and inhibitory learning. Two different cues (A and B) provide the information whether a third stimulus (X), presented in compound with either A or B, is paired with an aversive stimulus or not. More precisely, stimuli A and X are presented simultaneously and paired with aversive shock, and stimuli B and X were presented simultaneously without any aversive shock being presented. Typically, startle responses are used as the main outcome measure and typically, higher startle responses are seen in the presence of A and AX as compared to B and AB because B serves as a conditioned inhibitor.

**Aversive anticipation task** (Stout et al., 2021)

In the aversive anticipation task, colored circles are presented. The color of the circles predicts the presentation of either positive or negative images. Participants are asked to anticipate the type of image they are about to see, while the cue (i.e. the colored circle) is presented. Participants learn the association between cue and valence of the image, which can be observed in the anticipatory-potentiated startle responses.

**NPU-threattest** (Wolitzky-Taylor et al., 2014; Kreutzer and Gorka, 2021; Hall et al., 2022; Radoman et al., 2019)

The NPU-threat test (Schmitz and Grillon, 2012) was developed to assess short-duration (fear) and long-duration (anxiety) aversive states in human participants - both adult and pediatric samples. The experiment typically consists of three conditions with threat contingencies being explicitly instructed (i.e. instructed learning): a safe condition (neutral (N)), during which no aversive stimuli are presented, and two threat conditions. In the predictable threat condition (P), aversive events are administered predictably and are signaled by a cue. In the unpredictable conditioning (U), the aversive stimuli are administered unpredictably. Typically, the main outcome measure is the human startle reflex.

**Threat of shock paradigm** (Pole, 2007; Schellhaas et al., 2022; Young et al., 2018; Young et al., 2019)

The Threat of Shock Paradigm typically involves explicit verbal information (i.e. instructed learning) that participants may receive aversive shock with different probabilities in different experimental conditions such as high threat, medium threat and low or no threat. The different conditions are typically explicitly indicated through visual cues.

**Reward learning paradigms**

**Monetary incentive delay task** (Birn et al., 2017; Boecker-Schlier et al., 2016; DelDonno et al., 2019; Dennison et al., 2016; Dennison et al., 2019; Dillon et al., 2009; Hendrikse et al., 2022; Mehta et al., 2010; Morelli et al., 2021; Sheridan et al., 2018; Yang et al., 2021)

The monetary incentive delay (MID) task is a widely used decision-making task in humans embedded in the framework of reward processing see e.g. (Wilson et al., 2018) which also contains a learning element (see below). Typically, the task is implemented as an fMRI task for the investigation of neural mechanisms underlying motivational salience processes. Importantly, the MID contains two phases allowing to differentiate between two distinct aspects of reward processing: anticipation and feedback. A typical task contains three different types of stimuli presented in fixed order. First, a visual cue representing gain, loss or a neutral outcome is presented allowing to assess brain activation during the anticipation period. Second, a target cue is presented representing a prompt to perform a certain action (e.g. pressing a button as fast as possible). Of note, this learned, anticipatory response (as assessed through BOLD fMRI) to a reward in a monetary incentive delay (MID) task is conceptually comparable to BOLD fMRI responses during presentation of CS+ vs. CS- in threat learning, that is the anticipation of threat vs. safety. Likewise the MID can be understood as a learning element, because participants decrease response time to the rewarded cue over the course of the experiment providing evidence of instrumental or reinforcement learning where the presentation of a specific cue triggers an action that is rewarded. Following the anticipation phase, feedback about the outcome of the trial, that is (financial) gain, loss or neutral dependent on anticipation and performance is presented. Behavioral outcome measures of interest include reaction time, differences in gain vs. loss trials, as well as the total amount of rewards earned. For neuroimaging, a recent meta-analysis shows robust activation of the striatum, the anterior cingulate cortex, as well as the insula during anticipation of both gains and losses (Wilson et al., 2018). A child friendly version of the MID, that was designed to be visually appealing and engaging, requires children to virtually beat pinatas with variable numbers of stars as quickly as possible (Helfinstein et al., 2013). They are instructed that the number of stars determine the reward they receive at the end of the task.

**Monetary incentive saccade task** (Mueller et al., 2012) In the monetary incentive saccade task, a cue indicates whether the participant is supposed to make an eye movement toward (prosaccade) or away from (antisaccade) a target presented subsequently. The cue further informs the participants, whether they would win, lose or not receive reward or punishment upon correct eye movement. Participants learn to adapt their behaviour (i.e. direction and speed of saccade) in response to the different cues. Feedback indicating win or loss was displayed after each completed trial in the location of the correct eye movement target.

**Passive avoidance task** (Gerin et al., 2017; Blair et al., 2022; White et al., 2022) In the probabilistic passive avoidance task (White et al., 2013), participants learn which of four different stimuli are associated with a (higher) chance of winning or losing. In each trial, participants have to decide whether they want to actively approach (i.e. respond) or passively avoid (i.e. withhold) a response to a stimulus. In the subsequent feedback phase participants are then informed whether they lost or gained points and how much. Two of the four stimuli lead to a reward (high vs. low) in 70–80% of all trials and the other two lead to punishment (high vs. low) in 70–80% of all trials when responded to. Passive avoidance results in no feedback and no reward or punishment.

**Three-arm bandit task** (Cisler et al., 2019; Letkiewicz et al., 2022) Participants have to choose one out of three different stimuli to allocate money to. They have to learn that those stimuli have varying probabilities of a positive outcome and vary in the amount of the return ranging from more money than invested to no return at all. Besides the choices made and the reaction time during the decision phase, brain activation during the decision, anticipation (waiting for outcome) and feedback phase are of interest.

**Stimulus selection task** (Pechtel and Pizzagalli, 2013)

A stimulus selection task is a version of a reinforcement learning task with a phase during which contingencies are learned and a test phase during which decisions have to be made based on previously learned contingencies. During the learning phase participants are presented with different stimulus pairs with different probabilities of being rewarded. For example, stimulus pairs A-B, C-D, and E-F might have probabilities of 80 vs. 20%, 70 vs. 30%, and 60 vs. 40% chance of being rewarded when choosing one stimulus or the other. Participants are informed that they made the correct choice when they choose the stimulus that has a higher chance of leading to a reward for any given stimulus pair. The learning phase is completed when a certain performance criterion is reached. In the subsequent test phase, the familiar stimulus pairs from the learning phase are mixed with novel combinations of all stimuli presented before. No feedback is given during the test phase to prevent further learning. Instead the focus is on decision making based on previously learned information that needs to be employed here in a novel context.

**Card-guessing task** (Eckstrand et al., 2019; Romens et al., 2015)

During a reward-based card-guessing task (Delgado et al., 2000), participants are presented with a card in every trial and have to guess whether the card when revealed has a value higher or lower than 5. Their guess does not influence the actual outcome of the trial, which results in four different experimental conditions (i.e. win trials – expected win and actual win, loss trials – expected and actual loss, mixed trials – mismatch between expectation and outcome, neutral trials – no expectation and no change during outcome) and allows to evaluate brain activation patterns in anticipation of the outcome and during the feedback phase. Such decision-making processes are thought to be modulated by reinforcement learning processes because the decision in each trial is affected by previous rewards and punishments (Dong et al., 2014).

**Probabilistic (reinforcement) learning task** (Hanson et al., 2017; Kennedy et al., 2021; Wilkinson et al., 2021) Participants are presented with two stimuli (e.g. everyday objects) and are told to choose one of them via button press (van den Bos et al., 2012). This decision phase was followed by positive or negative feedback. Feedback is dependent on the unknown reinforcement schedule of each stimulus pair and has to be learned through trial and error. For example, in AB stimulus pairs, choosing stimulus A leads to positive feedback on 80% of trials (as opposed to 20% for stimulus B), whereas in CD pairs positive feedback is received in 70% and 30% of trials. The goal of the participants is to receive as much positive feedback as possible in order to maximize the reward earned.

**Probabilistic reversal learning task** (Wilkinson et al., 2021)

In the probabilistic reversal learning task (Cools et al., 2002), participants have to choose between rich and lean patterns in order to maximize points. The rich stimulus is rewarded in 80% of all cases, the lean stimulus in 20%. After a certain number of correct choices, contingencies are reversed. Measures of interest are win-stay probability which describes the probability that a participant chooses the same stimulus again if they just won with that stimulus and vice versa for lose-shift probability. Additionally, the number of rule changes that a participant reached is assessed. Moreover, a reinforcement learning model can be used to model learning rate as well as degree of choice variability. It further allows comparing an individual’s performance to an optimal performance.

**Signal detection task** (Morris et al., 2015) Participants are presented with two stimuli (e.g. short and long line), one of which was rewarded more often when chosen. A response bias or successful reward learning was indexed by a higher proportion of choices made that would lead to a reward.

**Stimulus context reversal paradigm** (Weiss et al., 2019) In this task (Levy-Gigi et al., 2014), stimuli (e.g. everyday objects) are shown in boxes overlaid on a neutral or salient (e.g. drug-related) context. In each trial, the participant has to decide whether to open the box or not. During the acquisition phase, the participant has to learn which combination of object in the box and background is associated with a positive and which with a negative outcome with the overarching goal to maximize the reward earned. After reaching a certain number of correct responses, the participant moves on to the retention phase during which the combinations from the acquisition phase are presented alongside new combinations with old boxes but new backgrounds (neutral or drug-related). In the trials with the changed background, the reward associated with a box is reversed such that previously rewarded boxes are no longer rewarded and vice versa. The participant is expected to learn that the change in the background indicates a change in the reward contingencies but that box - background combinations from the acquisition phase are not affected. The proportion of correct answers in the different phases relative to each other is used as the main outcome measure.

**Children’s gambling task** (Delgado et al., 2022)

Participants were presented with two decks of cards that could be differentiated visually by their back. One of the card decks was advantageous over the course of the experiment because more net reward could be earned while the other one was disadvantageous. Thus, the aim of the gambler is to make relatively more advantageous than disadvantageous choices.

**Patch foraging task** (Lloyd et al., 2021)

A patch foraging task is a sequential decision-making task in which participants have to optimize an explore/exploit trade-off (Lloyd et al., 2021). During this task a participant is set at a patch and can choose to exploit the patch and collect rewards there or move to a different patch instead. Importantly, the longer the participant remains within the same patch, the fewer rewards are available. This is called the depletion rate. When choosing to forage and move to a different patch the participant has to consider that the travel time between patches is not rewarded. Thus throughout this task the participant has to learn when it is best to explore and when to exploit. The initial richness of the new patch is based on the number of rewards on the first patch. The experimenter can further vary the richness of the environment. In a poor quality environment, an optimal forager would exploit each patch more, whereas a high-quality environment would require a higher degree of exploration in order to maximize rewards.

**Salience attribution test** (McCutcheon et al., 2019)

The salience attribution test is employed to measure aberrant and adaptive salience (Roiser et al., 2009). In this task, in each trial, one stimulus of four different categories is presented (e.g. blue animal, red animal, blue household object, red household object) spanning two dimensions (here type of objects and colour). Stimulus presentation is followed by a probe to which participants have to respond to as quickly as possible to maximize potential reward. At any given point, one dimension is relevant for the reward probability. One of the two stimuli of the relevant dimension has a high chance of being rewarded, the other stimulus has a low probability. The irrelevant dimension does not affect reward probability. Adaptive salience encompasses that participants learn to expect a higher reward for and respond with faster reaction times to a stimulus of the relevant dimension relative to the irrelevant dimension.

**Mixed appetitive and aversive conditioning** (Smith and Pollak, 2022) In a mixed Pavlovian conditioning paradigm (Metereau and Dreher, 2015), participants are exposed to different stimuli that are either followed by an appetitive or an aversive reinforcer. A neutral reinforcer can further be integrated as a control condition. In a typical trial, participants are presented with a stimulus and either after a certain delay or a button press, the reinforcer is presented. Often stimulus ratings of e.g. goodness or likeability are completed before and after conditioning or throughout in order to measure how the association with the reinforcer changed the subjective experience of the stimulus. If a button press was required or encouraged, response times can be used to model learning rates using the Rescorla and Wagner reinforcement learning framework (1972). Here, learning is understood as an adaptive process in which expectations are updated on a trial-by-trial basis based on expected and actual outcome.

**Associative learning task** (Wismer Fries and Pollak, 2017) A visually cued reward schedule task is designed to test children’s ability to learn associative connections with rewards in their environment and adapt their behaviour (Liu et al., 2000). Each trial is initiated by the child keeping a button pressed, which in turn triggers the presentation of a stimulus on the screen. The stimulus is either a coloured circle (in the colour discrimination version) or a geometric shape (in the shape discrimination version). As soon as those stimuli change to a different colour or shape in the respective version of the task, the child is instructed to release the button. Reaction time to the change as measured by the release of the button, is recorded. Children have to complete 1, 2, or 3 consecutive trials correctly before receiving a reward.

**Instrumental learning** (Patterson et al., 2013; Harms et al., 2018) In an instrumental learning task, a participant learns that an action such as a simple button press in response to a stimulus (Finger et al., 2008) or the execution of a certain sequence of button presses (Vogel-Sprott, 1967) leads to a reward. The reward schedule might indicate that all correctly performed actions are rewarded, or a partial reinforcement schedule might determine that a certain percentage of correctly performed actions is rewarded. A purely reward-based task can also be mixed with a component of punishment in which the response to a different stimulus or the performance of an incorrect action might lead to the loss of points to be maximized. Variations of an instrumental learning task might further involve a reversal condition in which the association between action and reward or punishment is reversed, that is actions that were previously rewarded are now punished and vice versa.

In Supplementary file 10, we provide additional results for likelihood heuristics (Lakens and Etz, 2017) as an indicator of the (relative) evidence for either the alternative (H1) or the null hypothesis (H0) in sets of studies that yield mixed results. In fact, it is highly unlikely that a given set of studies will all yield significant effects. With these tests, the probability of the observed ratio of statistically significant results and null effects is calculated under the assumption that either the H0 or the H1 is true (based on code provided by Lakens and Etz, 2017). As likelihood ratio tests do not provide quantitative meta-analytic evidence and are also not weighted by study quality, these results should be interpreted as heuristic estimates of the overall pattern and in light of their limitations.

For data analyses and visualizations as well as for the creation of the manuscript, we used the following R packages (R Version 4.2.3; R Development Core Team, 2023) and the R-packages *ade4* (Bougeard and Dray, 2018; Chessel et al., 2004; Dray et al., 2007; Dray and Dufour, 2007), *citr* (Version 0.3.2; Aust, 2019), *data.table* (Version 1.15.2; Dowle and Srinivasan, 2023), *dplyr* (Version 1.1.4; Wickham et al., 2023c), *forcats* (Version 1.0.0; Wickham, 2023a), *ggplot2* (Version 3.5.1; Wickham, 2016), *ggpubr* (Kassambara, 2023), *here* (Version 1.0.1; Müller, 2020), *knitr* (Version 1.45; Xie, 2015), *lubridate* (Version 1.9.3; Grolemund and Wickham, 2011), *papaja* (Version 0.1.2; Aust and Barth, 2022), *patchwork* (Version 1.2.0; Pedersen, 2022), *psych* (Version 2.4.3; Revelle, 2023), *purrr* (Version 1.0.2; Wickham and Henry, 2023d), *qgraph* (Epskamp et al., 2012), *RColorBrewer* (Version 1.1.3; Neuwirth, 2022), *readr* (Version 2.1.5; Wickham et al., 2023e), *readxl* (Version 1.4.3; Wickham and Bryan, 2023b), *reshape2* (Version 1.4.4; Wickham, 2007), *stringr* (Version 1.5.1; Wickham, 2022), *tibble* (Version 3.2.1; Müller and Wickham, 2023), *tidyr* (Version 1.3.1; Wickham et al., 2023f), *tidyverse* (Version 2.0.0; Wickham et al., 2019), *tinylabels* (Version 0.2.4; Barth, 2022), *viridis* (Garnier et al., 2022; Version 0.6.5; Garnier et al., 2023), and *viridisLite* (Version 0.4.2; Garnier et al., 2022).

## Results

**Figure 1.**
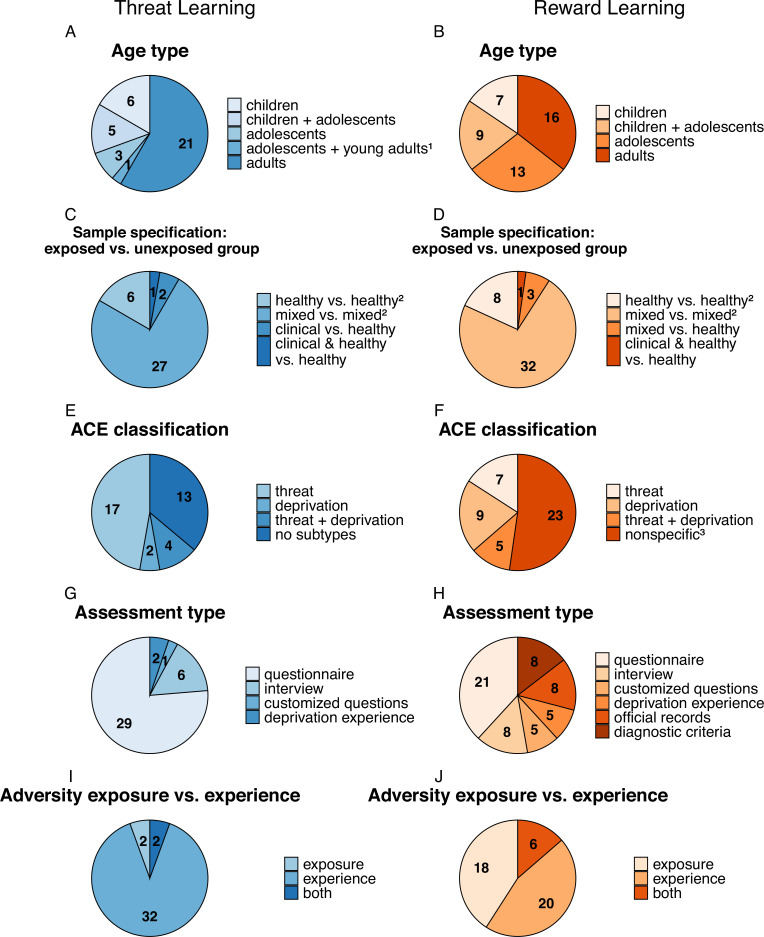
Sample characterization and ACE assessment instruments used in the studies included in the review on an association between ACEs and threat (n=38; A, C, E,G, I in blue) and reward (n=43; B, D, F, H, J in orange) learning processes. Numbers represent the number of studies to which a specific characteristic applies (note that these do not add up to the total number of studies as multiple characteristics may apply to a single study). Total sample sizes of the individual studies range from N=19 to N=11,360 (see Figure 1—figure supplement 1 for details). ^1^ Refers to participants aged 17–19 years; ^2^ Includes studies that assess ACEs dimensionally across all participants as well as studies that excluded participants with psychological disorders. ^3^ Includes studies that assess ACEs that cannot be classified as either threat or deprivation. Figure 1—figure supplement 1. Distribution of sample sizes across studies.

**Figure 2.**
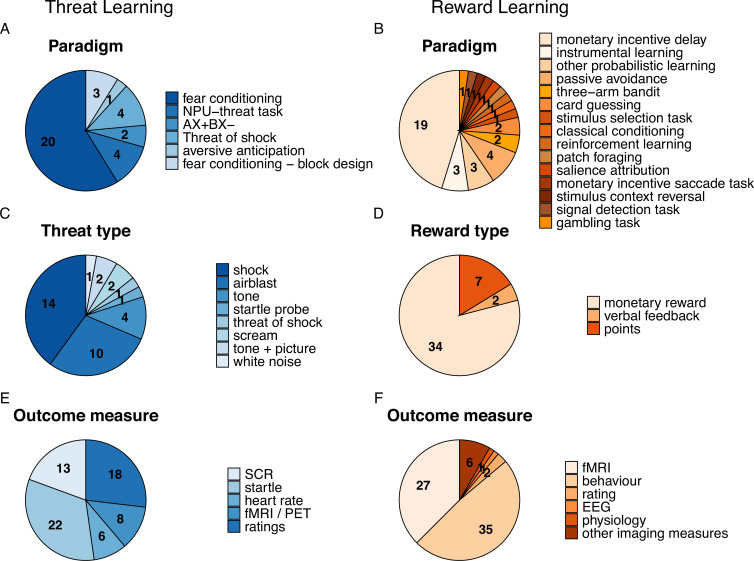
Paradigm specifications as well as outcome measures used in the studies included in this review for threat- (**A, C, E**, in blue) and reward-related learning (**B, D, F**, in orange) including paradigm type (**A, B**), type of threat or reward (**C, D**), and outcome measures used (**E, F**). Numbers represent the number of studies to which a certain specification applies (note that these do not add up to the total number of studies as multiple specifications may apply to a single study).

We identified a total of 3127 publications, and after screening of title, abstract, and full text, 81 articles investigating associations between ACEs and threat (n=38 publications) and reward learning (n=43 publications) were retained and included in analyses (see Supplementary file 1 for details). A breakdown of sample and ACE characteristics as well as experimental specifications of these studies are detailed in the following section and illustrated in Figure 1 and Figure 2, respectively.

**Figure 1—figure supplement 1.**
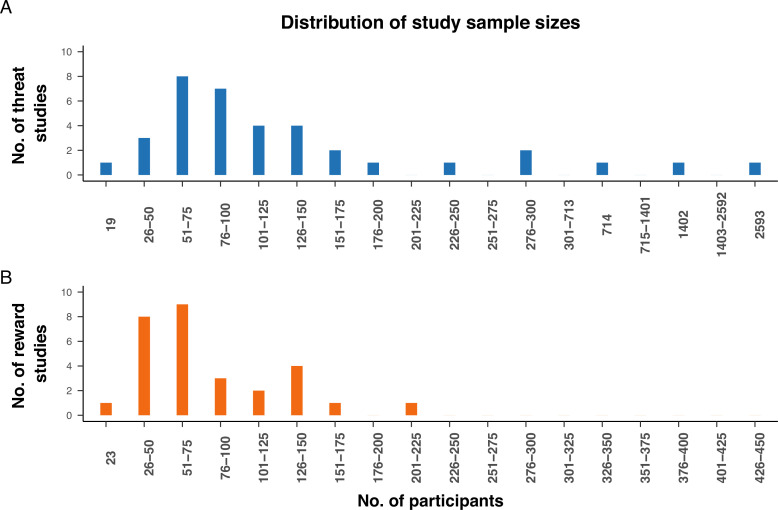
Distribution of sample sizes across studies.

### Summary of sample characteristics

Studies in the threat and reward learning fields were compared across several dimensions. First, in terms of participant age, studies in the threat field relied on roughly similar proportions of pediatric samples (i.e. children/adolescents) and samples of adults who report that they have experienced adverse events as children (Figure 1A and B), while in the reward field slightly more studies were conducted in children or adolescent samples. Second, across both fields, studies typically recruited healthy individuals who were screened for psychiatric disorders or community samples (i.e. participants drawn from the general population with little or no exclusion criteria for psychiatric symptoms or diagnoses). Only very few studies recruited clinical samples (Figure 1C and D), although in the threat field participants were often recruited from populations with high risk of experiencing trauma (e.g. low socioeconomic status regions, agencies that work with families exposed to violence or food banks). Third, while most studies across both research fields did not specifically aim to study specific ACE types (Figure 1E and F), threat-learning studies investigated predominantly samples reporting experience of threat-related adverse events (Figure 1E), as assessed mostly through self-reported questionnaires (Figure 1G). In contrast, studies from the reward learning field also used official records and diagnostic criteria for medical conditions as well as customized questions not part of a validated questionnaire for the assessment of ACEs (Figure 1H). Further, some studies from the reward learning field specifically focused on parental substance abuse (category ‘nonspecific’, Figure 1F) and reduced neighborhood quality or socioeconomic status. Fourth, studies from the threat learning field assessed mostly subjective adverse experiences rather than exposure to potentially adverse events or environments, whereas this ratio was more balanced across the reward learning studies (Figure 1I and J).

### Summary of paradigm characteristics

In the threat learning field, the majority of studies used a fear conditioning paradigm (71.05%) with varying experimental phases (e.g. acquisition training, extinction training, generalization, return of fear), and the remaining studies employed different forms of threat anticipation tasks (Figure 2A and see Box 1 for details on other paradigms such as threat of shock task, the NPU threat task or an aversive avoidance task) which involved learning by direct experiences or instructions. The paradigms employed in the reward-learning field were more heterogeneous spanning 15 different paradigms which illustrates that there is no prototypical reward learning task in the field (see Box 1 for an overview). The most commonly applied task was the MID task (19/43 studies). Other more common reward tasks included for example instrumental reward learning (3/43 studies), and probabilistic learning (3/43 studies) while other tasks were only employed in individual studies (see Figure 2B). As such, studies in the threat learning field used primary reinforcers (e.g. aversive stimuli), while the reward learning field used exclusively secondary reinforcers such as monetary reward (Figure 2C and D). In addition, while the threat learning field mainly employed subjective ratings and psychophysiological outcome measures (mainly skin conductance response (SCR) and fear potentiated startle (FPS)) to assess learning-driven changes, the reward learning field focused primarily on task behavior metrics, such as reaction time, accuracy, points earned, learning rates, and reward prediction error in addition to fMRI (Figure 2E,F). For further details on paradigm characteristics see Supplementary file 5 and Supplementary file 6.

### Study quality evaluation

Supplementary file 7, Supplementary file 8 and Supplementary file 9 show the assessments of study quality. The quality assessment tool used in the meta-analysis by Oltean et al., 2023 was adapted to the present review and focuses on sample characteristics and ACE assessment methods. Paradigm specifications were not evaluated, because objective criteria for assessing the quality of for example fear conditioning paradigms are not available. The quality assessment showed that, in both the threat and reward learning field, sample sizes were very small (less than 30 subjects per group) in a substantial amount of studies (33.3 %). However, 50% of studies had large sample sizes of 60 participants or more speaking for enhanced interpretability of results (see Figure 1—figure supplement 1 for a distribution of sample sizes). In addition, the samples were at least somewhat representative and subjects were screened for psychopathologies in the majority of the studies (88.9 %). In 50% of the studies only very few or even single ACE types were assessed (e.g. institutional rearing or low SES) - 38.9% using nonvalidated assessment instruments or composite scores from different instruments.

### Associations between exposure to ACEs and threat-related learning

The following results are structured according to the phases of a typical classical fear conditioning paradigm. During fear acquisition training, a neutral cue is repeatedly paired with an aversive stimulus (e.g. electrotactile stimulation; unconditioned stimulus, US). This pairing turns the previously neutral cue into a conditioned stimulus (CS+), which triggers conditioned fear responses (CRs), while a second neutral cue (CS-) is never paired with the US, serving as a safety stimulus. In a fear generalization phase, additional stimuli, perceptually similar to the CS+ and CS-, are presented (generalization stimuli; GSs) to assess generalization of the conditioned fear response. During a subsequent fear extinction training phase, both CS+ and CS- are presented without the US, leading to a gradual decrease in CRs. For details on the paradigm and the different variations employed in the studies included in this review, see Box 1.

*Acquisition.* Across a number of threat-related learning paradigms (n=21 studies), a consistent finding emerged of reduced discrimination between signals of threat and safety (i.e. CS+ and CS-) in individuals reporting ACEs. Reduced discrimination was rather consistently driven by blunted responding to cues signaling threat (i.e. CS+, anticipation of a negative image) in psychophysiological fear responses as measured with SCR (Harnett et al., 2019; Klingelhöfer-Jens et al., 2023; Kuehl et al., 2020; Machlin et al., 2019; McLaughlin et al., 2016), FPS (Lis et al., 2020; Stout et al., 2021; Thome et al., 2018), as well as self-reported fear ratings (Qiu et al., 2023; see Figure 2E for outcome measures used across studies). These results originate from samples of adults reporting ACEs as children (6 out of 10 studies) as well as children and adolescents (3 out of 5 studies; see Supplementary file 3 and Supplementary file 4 for details). However, three additional studies reported enhanced rather than reduced discrimination between signals of threat and safety in exposed individuals reporting a history of ACEs in SCRs (Marusak et al., 2021) and FPS (Zoladz et al., 2022) in females, but not males (Morrison et al., 2022). Of note, methodological challenges (use of unorthodox SCR scoring windows, Marusak et al., 2021; reporting of exclusively within-CS or within-group statistics,Morrison et al., 2022; Zoladz et al., 2022; without providing the crucial statistical test for an interaction effect, Gelman and Stern, 2006; Nieuwenhuis et al., 2011) render the claims of these studies difficult to interpret unambiguously. At the same time, however, similar methodological challenges were also evident in some studies that do show reduced threat discrimination in individuals reporting ACEs (Machlin et al., 2019; Thome et al., 2018, see discussion for details), highlighting the need for consistent methodological approaches in the fear conditioning field. Furthermore, although only rarely analyzed, severity of ACEs was also negatively correlated with responsiveness to danger signals (McLaughlin et al., 2016; Qiu et al., 2023; but see Estrada et al., 2020). A substantial number of studies also report no effects of ACEs on fear acquisition for at least one out of several outcome measures, including SCR (Bremner et al., 2005; Estrada et al., 2020; Huskey et al., 2022; Morrison et al., 2022; Scharfenort et al., 2016b), FPS (Deslauriers et al., 2018; Huskey et al., 2022; Jovanovic et al., 2009; Kuehl et al., 2020; Morrison et al., 2022; Rowland et al., 2022; Stenson et al., 2021; Thome et al., 2018; Zoladz et al., 2022), heart rate (Huskey et al., 2022), ratings of valence and arousal (Klingelhöfer-Jens et al., 2023; Qiu et al., 2023; Schellhaas et al., 2022), fear (McLaughlin et al., 2016; Scharfenort et al., 2016b), expectancy/contingency (Huskey et al., 2022; Klingelhöfer-Jens et al., 2023; Zoladz et al., 2022) as well as null findings also during re-acquisition (Bremner et al., 2005) and behavioral distance (Marusak et al., 2021). In addition, two studies suggest developmental effects of ACEs on threat learning in which successful CS discrimination at an earlier than typical age (i.e. already at 4–5 years, Machlin et al., 2019) and more adult-like functional connectivity between the hippocampus and prefrontal regions (Silvers et al., 2016) was observed.

*Generalization*. Patterns of reduced discrimination between signals of threat and safety following threat learning in individuals reporting ACEs also emerges in generalization phases (5 out of 7 studies: Klingelhöfer-Jens et al., 2023; Lange et al., 2019; Qiu et al., 2023; Thome et al., 2018; Zoladz et al., 2022). This was, however, again mostly driven by blunted CS+ responding in SCRs (Klingelhöfer-Jens et al., 2023), lower US expectancy ratings (Qiu et al., 2023), blunted valence and arousal ratings to the CS+ as well as enhanced ratings to the CS- (Lange et al., 2019) rather than differences in generalization gradients. A single study reported enhanced startle to the CS- in females reporting ACEs only (Zoladz et al., 2022), although no statistical results for males or females that do not report ACEs were provided. Of note, these effects matched the direction of effects for the same study during preceding acquisition training phase in Klingelhöfer-Jens et al., 2023 but were in the opposite direction in Zoladz et al., 2022 while no acquisition results were reported in Lange et al., 2019. No study reported enhanced CS discrimination during generalization, but a number of null findings emerged (ratings of US expectancy/contingency: Lange et al., 2019; Klingelhöfer-Jens et al., 2023; Zoladz et al., 2022, valence and arousal: Klingelhöfer-Jens et al., 2023, risk: Lis et al., 2020 and fear: Lange et al., 2019, FPS: Lis et al., 2020). Interestingly, generally no differences in generalization gradients were observed in these studies, even though one study (Zoladz et al., 2022) claims enhanced generalization in exposed females. As this study, however, did not observe any statistically significant differences between any of the conditioned and generalization stimuli, these results are difficult to interpret. On a behavioral level, trauma-exposed individuals reported highest threat uncertainty (longer reaction times) to highly-ambiguous GSs, while healthy controls reported most uncertainty when rating GSs that were most similar to the CS+ (Thome et al., 2018). In sum, a converging pattern emerged across studies whereby individuals reporting a history of ACEs show reduced discrimination between learned signals of danger and safety, primarily driven by blunted responding to signals of danger during fear acquisition training and generalization.

*Extinction*. For the extinction phase (N=12, whereof six do not report acquisition results), most studies report no effects of ACEs on extinction learning (SCR: Bremner et al., 2005; Huskey et al., 2022; Kuehl et al., 2020; Marusak et al., 2021; McLaughlin et al., 2016; Scharfenort et al., 2016b; Susman et al., 2021; FPS: France et al., 2022; Huskey et al., 2022; Kuehl et al., 2020; ratings of fear: Scharfenort et al., 2016b; McLaughlin et al., 2016 expectancy: Huskey et al., 2022; as well as heart rate: Bremner et al., 2005; Huskey et al., 2022; or behavioral distance: Marusak et al., 2021). Of the three studies that observed a significant association between ACEs and fear extinction, one reports higher US expectancy to the CS- with an increasing number of experienced traumatic events. The same study demonstrated an enhanced behavioral distance to the CS+ as compared to the CS- during extinction recall (but not extinction) for children reporting a history of ACEs compared to children reporting no history of ACEs (Marusak et al., 2021). Two other studies report enhanced SCR to the CS+ in children with a history of ACEs during extinction (Jenness et al., 2019; Milojevich et al., 2020). The results, however, are inconsistent across the two publications reporting results from the same experiment using the same sample: McLaughlin et al., 2016 and Machlin et al., 2019 report the results from a preceding fear acquisition training to the extinction phase referred to in Jenness et al., 2019 and Milojevich et al., 2020, respectively (outlined in detail in the discussion).

*General reactivity*. In addition to the overall pattern of blunted threat-learning, a number of studies also report heightened general (physiological) reactivity in ACE exposed individuals (FPS: Jovanovic et al., 2009; Kreutzer and Gorka, 2021; Pole et al., 2007; Rowland et al., 2022; Wolitzky-Taylor et al., 2014, interaction effects: Young et al., 2019; Zoladz et al., 2022, descriptive evidence: Morrison et al., 2022; SCR:Estrada et al., 2020, i.e. decreased habituation, interaction effects:Jovanovic et al., 2020; Scharfenort et al., 2016b; Young et al., 2019 and risk ratings:Lis et al., 2020; Thome et al., 2018). Of note, Kreutzer and Gorka, 2021 observed generally enhanced startle reactivity in individuals that have experienced interpersonal trauma (and higher trauma load), but blunted responding in individuals exposed to other trauma types (and lower trauma load) as compared to controls. In addition, Jovanovic et al., 2022 report a negative correlation of startle reactivity towards the CS+ and trauma exposure, but only in participants aware of experimental contingencies, while they report the opposite pattern for unaware participants. Decreased general reactivity, however, is only reported by one study using SCR (Klingelhöfer-Jens et al., 2023) and some studies report null findings (all outcome measures: Young et al., 2018; Huskey et al., 2022; Jovanovic et al., 2022; FPS and contingency ratings: Jovanovic et al., 2009; valence, arousal, contingency ratings: Klingelhöfer-Jens et al., 2023, heart rate: Young et al., 2019).

*Neuroimaging*. Neuroimaging results are presented separately and across all experimental phases, as only few studies report such findings. Results (see Figure 2E) for both amygdala and hippocampus activation during acquisition training were inconsistent across studies. For the amygdala, blunted CS discrimination and learning slopes (DeCross et al., 2022) as well as enhanced (Bremner et al., 2005) or reduced reactivity in individuals reporting ACEs (Harnett et al., 2019) and null findings (Silvers et al., 2016) are reported. For the hippocampus, higher activation for CS+ vs. CS- in individuals reporting ACEs (Silvers et al., 2016) or a negative association with ACEs (Harnett et al., 2019) and null findings (DeCross et al., 2022; Scharfenort et al., 2016b) are reported. Negative associations of neural activation with ACEs are further reported for the dorsolateral and ventromedial prefrontal cortex (Harnett et al., 2019). During fear generalization, no associations between ACEs were observed with (re-)activation of the amygdala, vmPFC (Lange et al., 2019), insula, dorsal anterior cingulate cortex (dACC), hippocampus, or vmPFC (Morey et al., 2015). During extinction recall, children reporting ACEs showed stronger (re-)activation in the dACC and the insula (but not in other regions of interest (ROIs)) to the extinguished CS+ as compared to controls in absence of group differences for the CS- (Marusak et al., 2021).

*Summary of results*. For fear acquisition, a total of 21 studies reported results. Of these studies, nine studies reported blunted responses to the threat cue in individuals with a history of ACEs (in any outcome measure). In contrast, three studies report enhanced responding to the threat cue in individuals with a history of ACEs (even though methodological challenges hamper a clearcut interpretation of these results, see introduction and discussion for details). For threat generalization, a total of seven studies reported results for a generalization phase. Of those, five reported blunted responding to threat cues in individuals reporting ACEs compared to controls. It should be noted that it is very unlikely that all studies of a larger number of studies will show significant results (Lakens and Etz, 2017). Therefore, it can be assumed that there is a tendency toward blunted responding to threat cues during (experimental) threat acquisition and generalization. In sum, we conclude that the results point towards generally blunted threat learning (see also likelihood ratio tests in Supplementary file 10).

### Associations between exposure to ACEs and reward-related learning

The systematic literature search for reward learning revealed a mix of null findings alongside the same amount of studies showing attenuated reward (learning) performance. Here, attenuated reward performance serves as an umbrella term describing a plethora of outcome measures across the different tasks (see Figure 2B) that share a common element of deficient behavior during probabilistic, reward-related reinforcement learning.

*Behavioral results*. Fourteen out of 28 studies reporting behavioral results show blunted responding to rewarding feedback following adverse experiences during childhood (see Figure 2F). The most commonly employed indicators of reward learning performance in these studies are measures of task performance (i.e. number of correct responses, points or reward earned) and speed of learning i.e, reaction time or learning rate (Delgado et al., 2022; Dennison et al., 2019; Harms et al., 2018; Patterson et al., 2013; Pechtel and Pizzagalli, 2013; Sheridan et al., 2018; Weiss et al., 2019; White et al., 2022; Wilkinson et al., 2021; Wismer Fries and Pollak, 2017). Deficient reward expectation or the violation thereof (i.e., prediction error) in the ACE group was restricted to studies employing reinforcement learning models (Hanson et al., 2017; Letkiewicz et al., 2022). Furthermore, in an exploration-exploitation task, reduced exploration and learning rate in exposed individuals is interpreted as a less optimal strategy to maximize rewards (Lloyd et al., 2022). In addition, in an incentive saccade task, exposed individuals show reduced responsiveness to rewarding feedback and less improvement of error performance under positive reinforcement (Mueller et al., 2012). One study (Dillon et al., 2009) reports that exposed participants rated rewarding cues as less positive in a MID task.

*Behavioral null results*. Importantly, the same number of studies (n=14) report null results for the association between ACEs and reward learning performance. Outcome measures were comparable between studies reporting significant and null findings e.g., response times, success rate or number of errors (Bjork et al., 2008; Boecker-Schlier et al., 2016; Cisler et al., 2019; Dennison et al., 2016; Dillon et al., 2009; Gonzalez et al., 2016; Mehta et al., 2010; Morris et al., 2015; Müller et al., 2015; Smith and Pollak, 2022; Weiland et al., 2013), measures of reward expectation and prediction error (Cisler et al., 2019). A more unique set of outcome measures – commission and omission errors - obtained from passive avoidance tasks was used in two studies with null findings (Blair et al., 2022; Gerin et al., 2017), which might suggest that while active reward learning seems to be affected by ACEs, reinforcement learning in the context of passive avoidance may not be.

*Neuroimaging results*. In addition to these behavioral measures, 17 studies additionally or exclusively used neuroimaging (i.e. fMRI) to compare brain activity elicited during reward anticipation or feedback in participants with and without a history of ACEs. Ten of these studies provide evidence for reduced neural (re)activation during the anticipation of a rewarding outcome following ACEs in distributed areas (Birn et al., 2017; Casement et al., 2014; Dillon et al., 2009; Mehta et al., 2010; Morelli et al., 2021) as well as in dedicated reward-processing circuitry including the ventral striatum and the insula (Boecker-Schlier et al., 2016; Gerin et al., 2017; Martz et al., 2022; Mullins et al., 2020; Yau et al., 2012). In contrast, six studies show enhanced (re)activation (e.g. in the thalamus, midbrain, insula, ventral striatum, inferior and medial frontal gyrus, dorsolateral prefrontal gyrus) during reward anticipation (Casement et al., 2014; DelDonno et al., 2019; Gonzalez et al., 2016; Hendrikse et al., 2022; Kwarteng et al., 2021; Romens et al., 2015). Three studies show ACE-related enhanced brain (re)activation (i.e. inferior frontal, cingulate and superior temporal gyrus as well as, prefrontal cortex, thalamus, putamen) during loss feedback or prediction errors (Birn et al., 2017; Gerin et al., 2017; Yang et al., 2021) including a linear relationship between number of traumatic events and ventral ACC activation (Eckstrand et al., 2019). An additional four studies show no difference between exposed participants and an unexposed control group during reward anticipation (Bjork et al., 2008; Müller et al., 2015; Weiland et al., 2013) and delivery (Boecker-Schlier et al., 2016).

*Summary of results*. Together, the current systematic literature review suggests evidence for reduced learning associated with rewards in participants with a history of ACEs. This finding is consistent with a recent meta-analysis (Oltean et al., 2023) that included 14 of the 43 studies reviewed here but had a broader focus on reward processing in general. The current review extends such general findings by focusing specifically on reward learning mechanisms and specifically including experiences of deprivation - which was in the studies included here operationalized for instance as low SES or institutional rearing: A total of 28 studies obtained and reported behavioral measures of reward learning performance. Of those studies, 14 studies reported a significant reduction in reward learning or the valuation of rewarding outcomes. Fourteen studies reported no significant group differences or associations with ACEs in reward learning. Again, it is important to note that it is highly unlikely that all studies from a large number of studies will show significant results- even when assuming that an effect does exist (Lakens and Etz, 2017). Therefore, we conclude that blunted reward learning in individuals reporting ACEs emerges as a consistent pattern of results. A more inconsistent pattern of results could be detected for fMRI activation patterns: 10 out of 17 studies suggest reduced (re-)activation in (not only) reward-related brain regions during the anticipation of a rewarding outcome. At the same time, seven studies showed the opposite result pattern with enhanced activation in midbrain regions and the activation of a wider brain network during reward anticipation. In sum, we conclude that the results point towards generally blunted reward learning (see also likelihood ratio tests in Supplementary file 10).

## Discussion

Here, we provide a systematic literature review on associations of ACEs with threat and reward learning while also focusing on general experimental and assessment practices in the field. The results paint a rather converging picture of blunted threat and reward learning (but no effect on safety learning or generalization) across different samples, ACE types and behavioral outcome measures.

### ACEs are linked to blunted threat learning

Blunted threat learning manifested primarily as reduced discrimination between learned signals of threat and safety, consistently driven by reduced responding specifically to the threat signal. Thus, reporting a history of ACEs appears to be associated with enduring effects on the capacity to differentiate environmental cues for aversive and safe outcomes. Of note, this pattern is distinct from what is typically observed in patients suffering from anxiety and stress-related disorders - enhanced responding specifically to the learned safety signal (e.g., Duits et al., 2015), which likewise results in reduced CS discrimination. Yet, it is important to note that not all patient samples in the threat learning field are characterized by exposure to adverse events, even though this is a strong risk factor associated with the development of psychopathologies. As most of the studies included in this review were conducted in non-clinical samples, it could be speculated that blunted conditioned threat responding may reflect a potential resilience factor, since participants were generally healthy despite a history of ACEs. Yet, this interpretation seems rather implausible in light of blunted CS+ responding also in studies including at-risk and patient samples (i.e., patients suffering from PTSD following childhood sexual abuse, Lange et al., 2019; Lis et al., 2020; Thome et al., 2018). Hence, we suggest that threat learning processes in individuals with a history of ACEs are distinct from those generally observed in anxiety patients. As ACEs are considered a potent risk factor for the development of psychopathology (e.g., Teicher et al., 2022), we speculate that exposed individuals may represent a distinct sub-group of patients which has so far been understudied due to a strong focus on group level inferences (patients vs. controls) and a lack of studies aiming to identify and utilize individual-level heterogeneity in response pattern.

### ACEs are linked to blunted reward learning

Reduced reward learning performance presented itself as reduced accuracy or reward earned and speed of learning in individuals with a history of ACEs which can be interpreted as blunted responding to, and integration of, reinforcing reward information. This finding is in line with a recent meta-analysis demonstrating a medium-sized association between ACEs and reward learning (in addition to other aspects of reward processing, Oltean et al., 2023). In contrast to the results presented in the meta-analysis, the current work has a much broader understanding of ACEs that includes adversity ranging from specific threatening experiences (e.g. sexual abuse) to long-lasting exposure to potentially adverse events for which the individual experience is less clear and may differ substantially between individuals (e.g. growing up in a low SES environment, parental substance abuse). The fact that we observe a similar pattern of results with a much broader definition of ACEs compared to the previous meta-analysis should be considered a strength of the current work and can be taken as evidence for the robustness of the identified effects. Our systematic investigation of the literature additionally revealed a mix of blunted (n=10) and enhanced (n=7) responding at the level of the brain during reward anticipation in a distributed set of brain regions. Further, evidence from several studies indicates that individuals with a history of ACEs show hyperresponsivity to losses, suggesting that the differences in reward anticipation are not due to diminished hedonic value (Birn et al., 2017) but rather reflect reduction in incentive salience (Olney et al., 2018). This interpretation is further supported by a study showing that individuals that report ACEs rate rewarding cues - not the reward itself - as less positive than unexposed individuals (Dillon et al., 2009). A more far-reaching interpretation would be that ACEs might have affected the development of the dopaminergic system, which has been discussed as a consequence of childhood adversity more broadly (for a discussion see Smith and Pollak, 2022). It would, however, be premature to draw strong conclusions about such neurobiological mechanisms without more conclusive evidence. From a neuropathological perspective, the collected findings are in line with findings in anhedonia which is similarly characterized by selective impairments in reinforced actions rather than reward responsiveness (Pizzagalli, 2014).

### Robustness of blunted threat and reward learning in light of heterogeneity in samples, procedures and operationalizations

Integrating the results from two research fields, we can conclude that the pattern of blunted behavioral responding to threat or rewards emerges independent of diverse sample or paradigm choices. Hence, we suggest that our conclusions hold for both research fields in general. For instance, this pattern of results is observed in pediatric samples (i.e. children or adolescents) versus adults, and regardless of whether individuals were exposed to only potentially adverse events or whether they had experienced severe threat or harm. Furthermore, our systematic literature review highlights the existence of rather homogeneous threat but quite heterogeneous reward learning paradigms. It is hence noteworthy that a rather converging pattern was observed not only in the threat learning field - in which similar paradigms are applied across studies - but also in the reward learning field despite diversity of the employed paradigms and hence the specific sub-processes investigated. We thus conclude that ACEs seem to be linked to generally blunted learning from threat and reward.

From an evolutionary perspective, it is generally adaptive to quickly learn to link environmental cues to threats and rewards to promote survival and thriving of the organism in normative environments. The often unpredictable nature of environments featuring adverse conditions may render it more adaptive to dampen behavioral modification by the erratic and infrequent signals of threat and reward. This pattern of responding, potentially resembling ‘emotional numbness’ (Litz and Gray, 2002), may be understood as a behavioral and neural recalibration to an ever-changing environment (Gerin et al., 2017) and thus as a coping strategy. It can hence be speculated that the pattern of blunted threat and reward learning in individuals reporting a history of ACEs may represent a coping strategy to environmental demands. From a therapeutic perspective, blunted threat and reward learning constitutes a potentially modifiable experience-dependent plasticity process that is at the core of many well established clinical interventions such as cognitive behavioral therapy and hence holds promise to be targeted in clinical interventions. More specifically, discrimination training may enhance the ability to discriminate between signals of threat and safety which may prevent overgeneralization. While successful clinical translation is based on individual level effects, the studies reviewed here nearly exclusively focus on group level inferences. It is hence an important next step for future work to disentangle whether these associations observed on average also map onto response patterns at the individual level. In addition, it is important to note that the scope of effects due to perturbed maturation of threat and reward learning processes following ACE might extend beyond simple associative learning to other experience-dependent domains. For example, ACE-driven blunted reward or threat signals during intricate social situations may interfere with the ability to acquire normative social cognition capacities that are based on accurate reinforcement, and impair social functioning as individuals grow up (Leblanc and Ramirez, 2020). Indeed, social learning has been shown to be malleable in children and affected by environmental factors such as caretaker education (Bulgarelli and Molina, 2016). Similarly, a recent meta-analysis demonstrated that children and adolescents with a history of ACE, and particularly deprivation, show executive functioning difficulties, such as poor inhibitory control (Johnson et al., 2021). Given that the extent of such capacities is believed to depend on the availability and presence of rewards (which affords experiences of inhibition, Burton et al., 2021), an environment of early-life deprivation may result in diminished ability to acquire adequate inhibitory control capacity that is required for general executive functioning. Given such potential links, future research is encouraged to examine the role of associative learning deficits due to ACE in perturbed development of cognitive and emotional functions that extend beyond simple threat and reward responding.

### No evidence from the literature for a link between specific ACE types and reward and/or threat learning

Importantly, our systematic literature review does not find evidence for distinct effects of specific ACEs with either reward or threat learning performance. In the threat learning field, four out of nine studies focusing exclusively on threat-related ACEs show blunted responding to threat-cues in individuals with a history of ACEs (Kuehl et al., 2020; Lis et al., 2020; McLaughlin, 2016; Thome et al., 2018), while three studies report null-findings (Jovanovic et al., 2009; Rowland et al., 2022; Stenson et al., 2021) and two studies enhanced threat responding (Marusak et al., 2021; Morrison et al., 2022). The pattern of results from the two available studies focusing exclusively on deprivation-related ACEs is comparable: One study investigating the relationship between deprivation-related ACEs and threat learning reports reduced CS discrimination driven by blunted responding to cues signaling threat in self-reported fear ratings in individuals with a history of ACEs as compared to controls (Qiu et al., 2023). The second study reports higher hippocampus (but not amygdala) (re)activation for the CS+ as compared to the CS- in individuals reporting a history of ACEs as well as a more ‘adult-like’ connectivity pattern in previously institutionalized individuals (Silvers et al., 2016). The latter study does not report results from psychophysiological measures or ratings rendering the interpretation of brain imaging data somewhat difficult. The pattern of results in the seven studies investigating associations between deprivation-specific experience and behavioral indices of reward learning also seem to match the general pattern of results reported here. Four studies (Delgado et al., 2022; Sheridan et al., 2018; White et al., 2022; Wismer Fries and Pollak, 2017) show reduced behavioral reward learning performance in the group exposed to deprivation experiences while three studies show no differences at the behavioral level (Gonzalez et al., 2016; Mehta et al., 2010; Smith and Pollak, 2022). Two additional studies did not provide behavioral measures of reward learning (Mullins et al., 2020; Romens et al., 2015). Of the studies focusing on threat-only experiences, four studies (Hanson et al., 2017; Harms et al., 2018; Letkiewicz et al., 2022; Pechtel and Pizzagalli, 2013) showed blunted responding, while only one study (Cisler et al., 2019) revealed a null result. Hence, we conclude that the results from the systematic literature search point towards generally blunted threat- and reward learning in individuals reporting a history of ACEs and blunted reward and threat learning - irrespective of the specific type of ACE (i.e. threat vs. deprivation; see also likelihood ratio tests in Supplementary file 10). Yet, most studies included in this review did not differentiate explicitly between adversity subtypes which does not allow us to draw firm conclusions on this from the literature. This observed pattern of results, however, stands in contrast to predictions by prominent theoretical accounts in the field (DMAP, Sheridan and McLaughlin, 2014; McLaughlin et al., 2014) that posit distinct (neuro-) biological effects of different ACE types. Yet, while such ‘splitting approaches’ (Smith and Pollak, 2021) are theoretically appealing and currently represent the dominant view in the field of threat learning (McLaughlin et al., 2019), this is a matter of ongoing debate. In brief, one challenge that has been highlighted is that certain aspects of ACEs are inseparable and can be conceptualized as different sides of the same coin. While co-occurrence of different dimensions are considered to be statistically controlled within the DMAP framework, it has been argued that this is impossible when different dimensions root in an identical event (e.g. a criminal neighborhood comes with threat of physical harm but most probable also with deprivational experiences such as the lack of safety and material resources). Additional criticism includes that there is little evidence for the core DMAP dimensions (i.e., deprivation and threat) mapping indeed onto specific neurobiological systems (Carozza et al., 2022; Smith and Pollak, 2021) or clinical outcomes (Witt et al., 2016). In addition a wealth of research suggests that the effects of experiences of early life adversity are cumulative, non-specific and rather unlikely to be tied to specific types of adverse events (Danese et al., 2009; Danese and Widom, 2020; Gehred et al., 2021; Smith and Pollak, 2022; Young et al., 2019).

### No evidence for an impact of developmental timing of ACEs on behavioral threat and reward learning

Another factor potentially influencing the association of learning patterns and childhood experiences is the age distribution of the sample and particularly the developmental timing of ACEs. More precisely, when investigating pediatric samples vs. adults who report ACEs, it is challenging to distinguish the effect of recency of the experiences from the developmental timing effects (discussed in Gee, 2021). In the current literature search, studies from the threat field relied approximately equally often on pediatric samples (i.e. children or adolescents), and adults with a history of ACEs (see Figure 1A and B) and the ratios of studies reporting blunted threat responding during fear acquisition vs null findings were similar across pediatric (3 out of 5) and adult samples (6 out of 10). In contrast, studies from the reward field relied more often on children or adolescents (n=29) than adult (n=16) samples. However, the ratios of children/adolescent to adult samples did not differ between studies who report blunted behavioral responding (12:5) vs null results (11:5) in the reward field. Thus, our literature review provides no evidence that the reported associations between ACEs and either threat or reward learning processes, or their direction may vary as a function of developmental timing of ACEs or recency of the experiences. However, these behavioral findings stand in contrast to a recently published meta-analysis reporting adversity-related alterations in amygdala and PFC BOLD activation in emotion processing, memory processing, inhibitory control, and reward processing tasks only in adult samples (having experienced adversity recently or during childhood), but not in children or adolescents (Gee, 2021; Hosseini-Kamkar et al., 2023). In sum, we do not find evidence for an impact of development timing of ACEs on threat and reward learning for behavioral outcome measures across the studies included in the current review. At the same time, there is evidence in the literature that such an effect might exist at the neural level (Hosseini-Kamkar et al., 2023).

### No evidence for specificity of an association between different outcome measures and the association between ACEs and threat or reward learning

Across the studies included in this work, a variety of different outcome measures has been used to study associations between ACEs and threat (e.g. SCR, FPS, ratings, fMRI BOLD response) as well as reward learning (e.g. behavioral measures, fMRI). Even though it is well known that different outcome measures tap into different underlying processes (Lonsdorf et al., 2017), the pattern of results observed does not seem to differ depending on the outcome measure. It should be noted though that these different outcome measures might be differentially sensitive to individual differences vs. group effects. Recently, the reliability of different outcome measures (i.e. SCR, fear ratings and BOLD fMRI) was compared (Klingelhöfer-Jens et al., 2022; see also Flournoy et al., 2024 for BOLD fMRI only) with the conclusion that there is no universally objectively most reliable measure, even though some specifications led to more reliable estimates. Yet, too little is known on this topic to meaningfully compare the studies accordingly.

### General challenges of investigating ACE type specific associations with threat and reward learning

**Figure 3.**
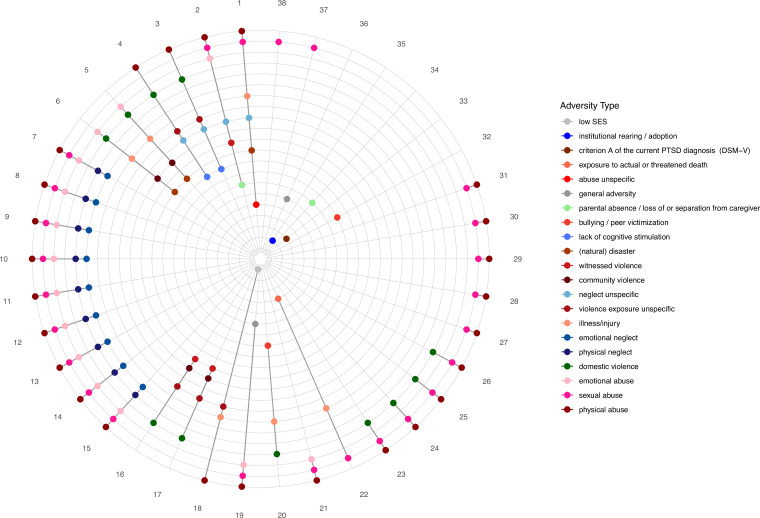
Distinct adversity types that were assessed in the 38 articles from the threat learning field. Numbers refer to the studies listed below the figure. The colored dots represent the adversity types listed in the legend on the right. Shades of red correspond to threat-related experiences, while blue dots correspond to deprivation-related experiences and green dots correspond to household dysfunction. Adversity types that did not fit into any of these categories were colored in gray. We included all adversity types that were considered as early adversity according to the studies and were assessed accordingly. The adversity types are being captured rather roughly as they represent the content of the assessment instruments as a whole or its subscales but not individual items. Figure 3—source data 1.Threat learning studies.

**Figure 4.**
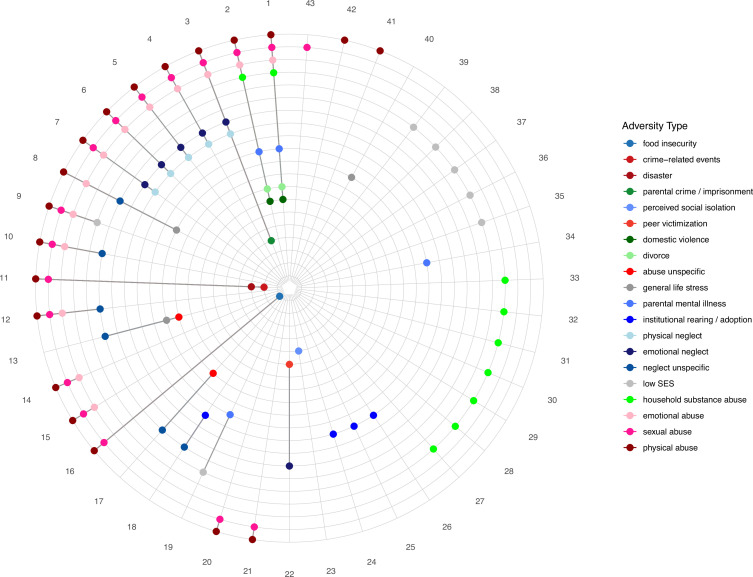
Distinct adversity types that were assessed in the 43 studies from the reward learning field. Numbers refer to the studies listed below the figure. The colored dots represent the adversity types listed in the legend on the right. Shades of red correspond to threat-related experiences, while dots in shades of blue correspond to deprivation-related experiences and dots in shades of green correspond to household dysfunction. Adversity types that did not fit into any of these categories were colored in gray. We included all adversity types that were considered as early adversity according to the studies and were assessed accordingly. The adversity types are being captured rather roughly as they represent the content of the assessment instruments as a whole or its subscales but not individual items. Studies Reward Learning. Figure 4—source data 1.Reward learning studies.

Our systematic inventory of ACE operationalization in the field of threat and reward learning (see Figures 3 and 4 as well as Supplementary file 2 for a list of questionnaires used in the included studies) highlights substantial heterogeneity in assessment tools and operationalization as a general challenge for cross-study comparison and drawing broad inference across this field (Koppold et al., 2023; Smith and Pollak, 2022). Likewise, the operationalization of ACEs varied greatly between studies (e.g. dimensional vs. categorical and specific vs. general adversity types) and often involved the generation of (artificial) groups from a continuous variable (Cohen, 1983) by median-split dichotomization (Jovanovic et al., 2020; Lange et al., 2019) or by applying cut-offs - that may vary even for a single questionnaire (e.g., CTQ, Bernstein et al., 1997; Bernstein and Fink, 1998). These data reduction approaches may obscure meaningful variability (Gee, 2021) and render the composition of adequate control groups challenging. Relatedly, it is a challenge that a number of studies focus exclusively on a specific ACE subtype without screening for other experiences (of no interest to this study). More precisely, studies focusing on deprivation-related ACEs oftentimes did not assess potential additional threat-related experiences. Further the studies focusing on threat-related ACEs typically did not assess potential additional deprivation-related experience. As a consequence, control and ACE groups may be characterized by similar overall levels of ACEs and may only differ with regard to one specific ACE (Kuehl et al., 2020; Marusak et al., 2021; McLaughlin et al., 2016; Morrison et al., 2022). In other words, participants assigned to the control group might have had severe adverse experiences not screened for (e.g. Jovanovic et al., 2020; Zoladz et al., 2022; Stout et al., 2021). This lack of broad screening might be particularly problematic as there is little support for distinct neurobiological effects of different adversity types as discussed above.

### Navigating methodological challenges

Taken together, we echo a recent call for ‘a basis for classifying adversity’ (Koppold et al., 2023; Pollak and Smith, 2021) and highlight that improving and potentially standardizing assessment, measurement and classification is urgently needed to improve comparability, replicability, and cumulative knowledge generation. For future research, data-driven approaches could be considered to address this problem of heterogeneity and co-occurrence of ACEs (Brieant et al., 2023). We also call for a more comprehensive in-depth phenotyping and characterization of the adverse childhood experiences (i.e. onset, developmental timing, controllability, e.g. Cloitre et al., 2009; Cowell et al., 2015), including subjective evaluations (Baldwin et al., 2019; Danese and Widom, 2020; Pollak and Smith, 2021), rather than simply screening for the exposure to events or environments that are potentially experienced as adverse (e.g. parental substance abuse), as well as for longitudinal studies in humans and complementary cross-species translational work that could aid to improve understanding about underlying mechanisms (see Box 2 for further topical and methodological directions). In addition to mastering assessment challenges, the field would profit from a generally increased focus on precision in reporting (e.g. sample and specification, methods) and adhering to published guidelines for processing psychological data (e.g., Boucsein, 2012; Blumenthal et al., 2005) and experimental procedures (Lonsdorf et al., 2017).

Further, we emphasize the need to report results of all (particularly preceding) experimental phases and stimuli rather than splitting them up into different publications (e.g., *Scientific salami slicing*; nature materials 2005). Even though hypotheses may be specific to extinction, group differences can only be meaningfully interpreted in light of the results during acquisition training. More precisely, differences observed during extinction training may not represent differences in extinction learning, but differences during threat learning that are transferred to later experimental phases. Unfortunately, this is not always common practice in the reviewed studies (e.g., Jenness et al., 2019; McLaughlin, 2016 and Machlin et al., 2019; Milojevich et al., 2020). In this context, we also highlight that the experimental paradigm and response quantification approaches need to be tailored to the outcome measures used. A mismatch may render results uninterpretable and may bias results if it goes undetected. For instance, skin conductance responses are slow physiological responses with the onset of a stimulus-bound peak typically occurring 1–4s after stimulus onset and the peak occurring even later than that. As a consequence, this makes a minimum inter-stimulus-interval of at least 5–6s necessary to avoid overlapping and hence confounded responses (unless model-based approaches are employed). If the inter-stimulus interval, however, is shorter, the response to the CS+ will be specifically confounded by the SCRs to the US (which co-terminates typically with CS offset) and render data uninterpretable. For instance, using CS specific scoring windows in an attempt to circumvent this problem (Marusak et al., 2021), or, when SCR scoring windows are longer than the stimulus presentations, a single ‘score’ may include SCRs to several successive trials including the US (Machlin et al., 2019; Milojevich et al., 2020).

Box 2. **Future directions to advance research on the association between ACEs and threat as well as reward learning processes**

**Methods-focused future directions**

**Assessment of ACEs**

- More attention to assessment tools: avoid assessment modifications (e.g. adding or dropping items) which threaten construct validity (Flake et al., 2017; Flake and Fried, 2019) and replicability. If modifications are unavoidable, these need to be reported with sufficient detail, ensuring construct validity, original factor structure, and profound scientific reasoning as outlined in Flake et al., 2017.
- Adhere to validated cut-offs and preferably report cut-off details rather than merely referring to previous publications.
- Consider (additional) assessment tools which allow for a fine-grained evaluation of potentially relevant ACE characteristics including onset and duration of exposure and controllability (e.g. MACE, Teicher and Parigger, 2015; although following Zorowitz and Tuominen, 2022 it lacks subscale specificity) as well as social aspects (e.g. social support).
- Consider making materials openly available (e.g. questionnaires, interviews used) to facilitate cumulative knowledge generation.
- Consider significant sample differences based on retrospective or prospective assessment of early life adversity and related implications on measurement selection (e.g. questionnaire or interview; as mentioned in Baldwin et al., 2019).
- Include subjective evaluations as there is converging evidence that risk for psychopathology develops based on subjective rather than objective evaluations (Baldwin et al., 2019; Danese and Widom, 2020; Pollak and Smith, 2021).

**General methodological recommendations**

- Provide sufficient details on the sample, paradigm, data recording and processing (consider supplementary material in case of space restraints) and avoid referring exclusively to previous work which can result in reference chains ending with implausible or ambiguous information.
- Adhere to published methodological guidelines for data recording, experimental design and terminology. For instance, short CS-US intervals and a failure to account for these in SCR response quantification may result in artificially enhanced CS+ responses due to the US and CS+ response being inseparable (as in Machlin et al., 2019; Milojevich et al., 2020).
- Avoid common statistical errors (as e.g. Makin and Orban de Xivry, 2019) such as inferring group differences from a significant within-group effect in one group and a non-significant within-group effect in a second group (the same applies for within-CS effects).
- Provide statistics not only for significant effects but also for non-significant results as well as post-hoc tests to avoid ambiguity and support cumulative knowledge generation.
- Consider providing single-trial data and individual-level data in visualizations (Weissgerber et al., 2015; potentially in supplementary material) to allow for a more comprehensive interpretation including habituation processes.
- Avoid “salami-slicing” and report results for all experimental phases. If results need to be published separately for different experimental phases, clearly highlight this as well as explicitly refer to the results reported previously. It hampers cumulative knowledge generation if publication 1 reports a significant effect for fear acquisition training but not extinction while publication 2 in the same sample publishes no differences in the last trial of acquisition (without referring to the previous work) and a significant effect on extinction in an outcome measure not included in publication 1 (as in Jenness et al., 2019; Machlin et al., 2019; McLaughlin et al., 2016; Milojevich et al., 2020).
- In case results include higher order interactions, consider discussing whether the study has acceptable power for interpreting them.
- Differences in responding to unconditioned stimulus such as threat and reward signals themselves should be considered.
- Tasks vary substantially and may not tap into the same processes even though they claim so (e.g. in the blocked fear conditioning design, the learning blocks with unreinforced CSs might rather be extinction learning, Machlin et al., 2019; Milojevich et al., 2020).
- Progress will be supported and facilitated by increasing data sharing practices (Ehlers and Lonsdorf, 2022) as only ten out of 81 studies provided open data (see Supplementary file 5 and Supplementary file 6).
- Include information on potential group differences to reinforcers (e.g. unconditioned threat and reward cues) as these may underlie group differences in learning.

**Topical directions**

- Provide a more comprehensive sample characterization of ACEs beyond a specific study focus to support cumulative knowledge generation and cross-study integration (e.g. provide a comprehensive experience profile for participants including control groups even though the study focus is specifically on household violence).
- Longitudinal developmental samples may aid the identification of mechanisms.
- Increased focus on variability between ACEs (e.g. developmental timing, protective factors) may aid the identification of mechanisms (Gee, 2021).
- Investigate potential mediators including epigenetics, neuroendocrine as well as immunological and neurobiological aspects.
- Increased focus on psychometric properties and reliability of the used measures is key for individual-level investigations and different from those suitable for group-level inferences.
- Furthermore, as ethical considerations restrict research in humans to observational studies, complementary cross-species translational work, including animal models in which life histories can be actively generated, will be important for testing mechanistic hypotheses on risk and resilience in the future.

### Summary and outlook

In sum, this work summarizes and integrates evidence from two potential mechanistic routes on how ACEs, a potent risk factor for psychopathology, lead to blunted responding to environmental cues supporting reward- and threat-related learning processes. Differences in samples (children vs. adults, clinical vs. healthy), different paradigms, and considerable variance in the operationalisation and assessment of ACEs as well as different subtypes or dimensions of ACEs do not appear to have a systematic influence on this pattern of results. The fact that blunted responding to threat and reward following early adversity is such a robust finding in the existing literature, underscores that these altered learning mechanisms are a promising target for tailored clinical prevention and intervention programs. Yet, we also identify a number of challenges - foremost with respect to ACE assessment and methodological precision - that hamper cumulative knowledge generation as well as progress in the field. We call for an increased focus on measurement (homogenization) as well as studies in larger cohorts, cross-lab collaboration as well as increased data sharing practices to achieve the statistical power or leveraging replicable and robust insights. Such large scale studies hold promise to shed light on the substantial heterogeneity in individual risk and resilience trajectories and will allow moving beyond group averages and capitalizing on individual differences (see Box 2). It should be noted that threat and reward learning are certainly not the only potential mechanistic routes that link ACEs and psychopathology. Other potentially relevant mechanistic routes that involve adaptation to adverse environments may be linked to the risk to psychopathology, such as social cognition, executive functions, emotion regulation and the quality of an individuals’ social network (McCrory et al., 2017; McCrory et al., 2022). While addressing all these diverse potential mechanisms is beyond the scope of this work, future research should consider these factors in the study of associations between psychopathology and ACEs.

## Data Availability

This article has been posted on a preprint server: https://doi.org/10.31234/osf.io/nfpb. The manuscript was written in R markdown. Data and code to generate and reproduce the manuscript file are openly available in Zenodo at: https://doi.org/10.5281/zenodo.11636897. The following dataset was generated: RugeJ EhlersMR KastrinogiannisA Klingelhöfer-JensM KoppoldA AbendR LonsdorfTB 2024How adverse childhood experiences get under the skin: A systematic review, integration and methodological discussion on threat and reward learning mechanisms.Zenodo10.5281/zenodo.11636897PMC1125172539012794

## Additional files

Supplementary file 1. Details on the systematic literature search, PRISMA flow chart.

Supplementary file 2. List of questionnaires used in the included studies.

Supplementary file 3. Sample specifications of the threat learning studies.

Supplementary file 4. Sample specifications of the reward learning studies.

Supplementary file 5. Paradigm specifications of the threat learning studies.

Supplementary file 6. Paradigm specifications of the reward learning studies.

Supplementary file 7. Quality assessment tool.

Supplementary file 8. Quality assessment of the threat learning studies.

Supplementary file 9. Quality assessment of the reward learning studies.

Supplementary file 10. Likelihood ratio tests.
