# Supplementary material for: How adverse childhood experiences get under the skin: A systematic review, integration and methodological discussion on threat and reward learning mechanisms
Source: eLife. 2024 Jul 16;13:e92700. doi: 10.7554/eLife.92700 (PMC11251725; doi:10.7554/eLife.92700)
Supplement: Figure 3—source data 1. [file elife-92700-fig3-data1.docx]

| **Studies Threat Learning** | | | | | | | |
| --- | --- | --- | --- | --- | --- | --- | --- |
| 1 | Pole (2007) | 11 | Estrada (2020) | 21 | Jovanovic (2009) | 31 | Lis (2020)#4 |
| 2 | Wolitzky-Taylor (2022) | 12 | Klingelhöfer-Jens (under review) | 22 | Kreutzer (2021) | 32 | Radoman (2019) |
| 3 | Milojevich (2019)#1 | 13 | Stout (2021) | 23 | Susman (2021) | 33 | Huskey (2022) |
| 4 | Machlin (2019)#1 | 14 | Zoladz (2022) | 24 | Jenness (2018)#2 | 34 | Qiu (2022) |
| 5 | France (2022) | 15 | Lange (2018) | 25 | McLaughlin (2016)#2 | 35 | Silvers (2016) |
| 6 | Jovanovic (2022) | 16 | Stenson (2021) | 26 | DeCross (2022) | 36 | Scharfenort (2016) |
| 7 | Deslauriers (2018) | 17 | Jovanovic (2020) | 27 | Young (2019)#3 | 37 | Rowland (2022) |
| 8 | Hall (2022) | 18 | Harnett (2019) | 28 | Young (2018)#3 | 38 | Morrison (2022) |
| 9 | Schellhaas (2022) | 19 | Bremner (2005) | 29 | Kuehl (2020) |  |  |
| 10 | Morey (2015) | 20 | Marusak (2020) | 30 | Thome (2018)#4 |  |  |
|  | # overlapping sample |  |  |  |  |  |  |

**Figure 3 Source data 1**
