# Supplementary material for: How adverse childhood experiences get under the skin: A systematic review, integration and methodological discussion on threat and reward learning mechanisms
Source: eLife. 2024 Jul 16;13:e92700. doi: 10.7554/eLife.92700 (PMC11251725; doi:10.7554/eLife.92700)
Supplement: Figure 4—source data 1. [file elife-92700-fig4-data1.docx]

**Figure 4 Source data 1**

| **Studies Reward Learning** | | | | | | | |
| --- | --- | --- | --- | --- | --- | --- | --- |
| 1 | Patterson (2013) Exp2 | 12 | Dennison (2016) | 23 | Smith (2022) | 34 | Morris (2015) |
| 2 | Patterson (2013) Exp1 | 13 | Birn (2017) | 24 | Wismer Fries (2017) | 35 | White (2022) |
| 3 | Lloyd (2022) | 14 | Weiss (2019) | 25 | Sheridan (2018) | 36 | Romens (2015) |
| 4 | Yang (2021) | 15 | Dillon (2009) | 26 | Mehta (2010) | 37 | Mullins (2020) |
| 5 | Hendrikse (2022) | 16 | Dennison (2019) | 27 | Kwarteng (2021) | 38 | Gonzalez (2016) |
| 6 | DelDonno (2019) | 17 | Wilkinson (2019) | 28 | Weiland (2013) | 39 | Delgado (2022) |
| 7 | Blair (2022) | 18 | Mueller (2012) | 29 | Martz (2022) Sample 1 | 40 | Kennedy (2021) |
| 8 | Boecker-Schlier (2016) | 19 | Morelli (2021) | 30 | Martz (2022) Sample 2 | 41 | Harms (2017) |
| 9 | McCutcheon (2019) | 20 | Letkiewicz (2022)# | 31 | Bjork (2008) | 42 | Hanson (2017) |
| 10 | Gerin (2017) | 21 | Cisler (2019)# | 32 | Müller (2014) | 43 | Pechtel (2013) |
| 11 | Eckstrand (2019) | 22 | Casement (2014) | 33 | Yau (2012) |  |  |
|  | # overlapping sample |  |  |  |  |  |  |
