# Supplementary material for: How adverse childhood experiences get under the skin: A systematic review, integration and methodological discussion on threat and reward learning mechanisms
Source: eLife. 2024 Jul 16;13:e92700. doi: 10.7554/eLife.92700 (PMC11251725; doi:10.7554/eLife.92700)
Supplement: Supplementary file 1. [file elife-92700-supp1.docx]

# Supplementary file 1 Systematic literature Search

The literature search was conducted collaboratively with help of the free online tool ‘rayaan’ (<https://www.rayyan.ai/>) by J.R., A.Ka., M.R.E. and T.B.L. using search terms outlined in Supplementary Table 1, which also provides an overview of the number of publications identified, retained and excluded. We retained a total of 81 publications that were included in the systematic literature review with 38 and 43 publications investigating an association between ACEs and threat as well as reward learning processes, respectively. We decided to exclude conference papers as well as gray literature as the results from these kinds of publications might still change and did not yet undergo a structured review process. Note that five additional studies, that were not identified through the systematic literature search, were included as a recent meta-analysis (Oltean et al., 2022) included them and we considered them relevant. In addition, in the threat field, the results from the acquisition and extinction training phase of one study were reported in two different publications in two cases ((Jenness et al., 2019; McLaughlin et al., 2016; and Machlin et al., 2019; Milojevich et al., 2020)). Therefore, it should be noted that we only show methodological results for the 36 distinct studies from the total of 38 publications in the respective figures. It was challenging to reconcile the respective results as these were not entirely consistent across these publications deriving from the same study. More precisely, one publication reports a significant effect during acquisition training but not extinction (McLaughlin et al., 2016) and the second study reports a significant effect during extinction training but not during the last trial of acquisition (Jenness et al., 2019). In addition, in two cases, two publications rely on an overlapping sample reflecting different timepoints during data collection (Lis et al., 2020; Thome et al., 2018; Young et al., 2019; as well as Young et al., 2018). We further included one unpublished study from our group (Klingelhöfer-Jens et al., under review) in the review. Our literature search focused on associations between ACE’s and threat as well as reward-related learning processes. Some of the identified studies focused additionally or even primarily on higher order interactions or used learning performance as predictor. These findings will not be described in detail here as they are beyond the focus of our work. These additional results include for instance higher-order interactions (e.g., 3- or 4- way interactions) with childhood adversity and a number of variables such as specific genotypes (COMTval148met, BDNFval66met, ADCYAP1R1 (Deslauriers et al., 2018; Jovanovic et al., 2020; Young et al., 2018)), volume in specific brain regions such as the hippocampus and corpus callosum (Young et al., 2019), stimulus intensity and the modulation of activation in certain brain areas (e.g. insula, LC and thalamus, Morey et al. (2015)) as well as interactions between threat-related reactivity and childhood adversity on symptom scores (e.g., problematic alcohol, anxiety and depression Hall et al. (2022); Radoman et al. (2019)) or interactions with subsequent memory tasks (Schellhaas et al., 2022).

## PRISMA Flow Chart

| **Associations between exposure to ACEs and** | **threat learning processes** | **reward learning processes** |
| --- | --- | --- |
| date search conducted | 2022-12-08 | 2022-12-09 |
| search terms pubmed | (("fear conditioning"[All Fields] OR "threat conditioning"[All Fields] OR "conditioned inhibition"[All Fields] OR "conditioned inhibition"[All Fields] OR ("fear learning"[All Fields] OR "threat learning"[All Fields] OR "extinction learning"[All Fields]) OR ("return of fear"[All Fields] OR "extinction retention"[All Fields] OR "fear generalization"[All Fields] OR "threat generalization"[All Fields] OR "aversive anticipation"[All Fields] OR "threat of shock"[All Fields] OR "fear-potentiated"[All Fields] OR "fear inhibition"[All Fields])) **AND** ((("childhood"[All Fields] OR "early"[All Fields] OR "youth"[All Fields] OR "adolesc*"[All Fields]) **AND** ("adversity"[All Fields] OR "adversities"[All Fields] OR "maltreatment"[All Fields] OR "abuse"[All Fields] OR "neglect"[All Fields] OR "stress"[All Fields] OR "trauma"[All Fields] OR "deprivation"[All Fields] OR "institutionalization"[All Fields] OR "orphanage"[All Fields] OR "adoption"[All Fields] OR "harassment"[All Fields] OR "bullying"[All Fields] OR "household violence"[All Fields] OR "domestic violence"[All Fields] OR "poverty"[All Fields] OR "low SES"[All Fields] OR "food insecurit*"[All Fields])) OR "adverse childhood experiences"[MeSH Terms] OR "early childhood"[All Fields])) **AND** (humans[Filter]) | ("childhood" OR "early" OR “youth” OR "adolesc*") AND ("advers*" OR "maltreatment" OR "abuse" OR "neglect" OR "stress" OR "trauma" OR "deprivation" OR "institutionalization" OR "orphanage" OR "adoption" OR "harassment" OR "bullying"OR "household violence" OR "domestic violence" OR "poverty" OR "low SES" OR "food insecurit*") AND ("reward" OR "reinforcement" OR "probabilistic") AND (“learning” OR “anticipation” OR “performance”) NOT ("mice" OR "mouse" OR "rodents" OR "rabbits") |
| search terms Web of Science | ALL= ( "fear conditioning" OR "threat conditioning" OR "conditioned inhibition" OR "fear learning" OR "threat learning" OR "extinction learning" OR "return of fear" OR "extinction retention" OR "fear generalization" OR "fear generalization" OR "threat generalization" OR "aversive anticipation" OR "threat of shock" OR "fear-potentiation" OR "fear-potentiated" OR "fear inhibition") **AND** ALL =("childhood" OR "early" OR “youth” OR "adolesc*") **AND ALL=**  ("advers*" OR "maltreatment" OR "abuse" OR "neglect" OR "stress" OR "trauma" OR "adverse childhood experiences" OR "early childhood" OR "deprivation" OR "institutionalization" OR "orphanage"OR "adoption" OR "harassment" OR "bullying" OR "household violence" OR "domestic violence" OR "poverty" OR "low SES" OR "food insecurit*")) | ALL=("childhood" OR "early" OR “youth” OR "adolesc*") AND ALL=("advers*" OR "maltreatment" OR "abuse" OR "neglect" OR "stress" OR "trauma" OR "deprivation" OR "institutionalization" OR "orphanage" OR "adoption" OR "harassment” OR "bullying" OR "household violence" OR "domestic violence" OR "poverty" OR "low SES" OR "food insecurit*" ) AND ALL=("reward" OR "reinforcement" OR "probabilistic") AND ALL=(“learning” OR “anticipation” OR “performance”) NOT ALL=("mice" OR "mouse" OR "rodents" OR "rabbits") |
| number of identified publications | 1222  PubMed: 281  Web of Science: 941) | 1905 PubMed: 761 Web of ‘Science: 1144 |
| number of publications after exclusion of duplicates | 1110 | 1543 |
| Number of publications after title and abstract screening | 105 | 99 |
| Number of publications after full text screening | 37+1* | 38 + 5** |

*An additional unpublished study was included (Klingelhöfer-Jens et al., under review).
**An additional 5 publications were included based on a recent meta-analysis on childhood adversity and reward processing (Oltean et al., 2022) that were, however, not identified through the systematic literature search.

# References

Deslauriers, J., Acheson, D. T., Maihofer, A. X., Nievergelt, C. M., Baker, D. G., Geyer, M. A., Risbrough, V. B., & Marine Resiliency Study Team. (2018). *COMT* Val158met polymorphism links to altered fear conditioning and extinction are modulated by PTSD and childhood trauma. *Depression and Anxiety*, *35*(1), 32–42. <https://doi.org/10.1002/da.22678>

Hall, O. T., Phan, K. L., & Gorka, S. (2022). Childhood Adversity and the Association Between Stress Sensitivity and Problematic Alcohol Use in Adults. *Journal of Traumatic Stress*, *35*(1), 148–158. <https://doi.org/10.1002/jts.22709>

Jenness, J. L., Miller, A. B., Rosen, M. L., & McLaughlin, K. A. (2019). Extinction Learning as a Potential Mechanism Linking High Vagal Tone with Lower PTSD Symptoms among Abused Youth. *Journal of Abnormal Child Psychology*, *47*(4), 659–670. <https://doi.org/10.1007/s10802-018-0464-0>

Jovanovic, T., Stenson, A. F., Thompson, N., Clifford, A., Compton, A., Minton, S., van Rooij, S. J. F., Stevens, J. S., Lori, A., Nugent, N., Gillespie, C. F., Bradley, B., & Ressler, K. J. (2020). Impact of ADCYAP1R1 genotype on longitudinal fear conditioning in children: Interaction with trauma and sex. *Neuropsychopharmacology*, *45*(10), 1603–1608. <https://doi.org/10.1038/s41386-020-0748-2>

Klingelhöfer-Jens, M., Hutterer, K., Schiele, M., Leehr, E. J., Schümann, D., Rosenkranz, K., Böhnlein, J., Repple, J., Deckert, J., Domschke, K., Dannlowski, U., Lueken, U., Reif, A., Romanos, M., Zwanzger, P., Pauli, P., Gamer, M., & Lonsdorf, T. B. (under review). *Reduced discrimination between signals of danger and safety but not overgeneralization is linked to exposure to childhood adversity in a large sample of healthy adults (under review)*.

Lis, S., Thome, J., Kleindienst, N., Mueller-Engelmann, M., Steil, R., Priebe, K., Schmahl, C., Hermans, D., & Bohus, M. (2020). Generalization of fear in post-traumatic stress disorder. *Psychophysiology*, *57*(1). <https://doi.org/10.1111/psyp.13422>

Machlin, L., Miller, A. B., Snyder, J., McLaughlin, K. A., & Sheridan, M. A. (2019). Differential Associations of Deprivation and Threat With Cognitive Control and Fear Conditioning in Early Childhood. *Frontiers in Behavioral Neuroscience*, *13*, 80. <https://doi.org/10.3389/fnbeh.2019.00080>

McLaughlin, K. A., Sheridan, M. A., Gold, A. L., Duys, A., Lambert, H. K., Peverill, M., Heleniak, C., Shechner, T., Wojcieszak, Z., & Pine, D. S. (2016). Maltreatment Exposure, Brain Structure, and Fear Conditioning in Children and Adolescents. *Neuropsychopharmacology : Official Publication of the American College of Neuropsychopharmacology*, *41*(8), 1956–1964. <https://doi.org/10.1038/npp.2015.365>

Milojevich, H. M., Machlin, L., & Sheridan, M. A. (2020). Early adversity and children’s emotion regulation: Differential roles of parent emotion regulation and adversity exposure. *Development and Psychopathology*, *32*(5), 1788–1798. <https://doi.org/10.1017/S0954579420001273>

Morey, R. A., Dunsmoor, J. E., Haswell, C. C., Brown, V. M., Vora, A., Weiner, J., Stjepanovic, D., Wagner, H. R., VA Mid-Atlantic MIRECC Workgroup, & LaBar, K. S. (2015). Fear learning circuitry is biased toward generalization of fear associations in posttraumatic stress disorder. *Translational Psychiatry*, *5*(12), e700. <https://doi.org/10.1038/tp.2015.196>

Oltean, L.-E., Șoflău, R., Miu, A. C., & Szentágotai-Tătar, A. (2022). Childhood adversity and impaired reward processing: A meta-analysis. *Child Abuse & Neglect*, 105596. <https://doi.org/10.1016/j.chiabu.2022.105596>

Radoman, M., Akinbo, F. D., Rospenda, K. M., & Gorka, S. M. (2019). The impact of startle reactivity to unpredictable threat on the relation between bullying victimization and internalizing psychopathology. *Journal of Psychiatric Research*, *119*, 7–13. <https://doi.org/10.1016/j.jpsychires.2019.09.004>

Schellhaas, S., Schmahl, C., & Bublatzky, F. (2022). Social threat and safety learning in individuals with adverse childhood experiences: Electrocortical evidence on face processing, recognition, and working memory. *European Journal of Psychotraumatology*, *13*(2), 2135195. <https://doi.org/10.1080/20008066.2022.2135195>

Thome, J., Hauschild, S., Koppe, G., Liebke, L., Rausch, S., Herzog, J. I., Müller-Engelmann, M., Steil, R., Priebe, K., Hermans, D., Schmahl, C., Bohus, M., & Lis, S. (2018). Generalisation of fear in PTSD related to prolonged childhood maltreatment: An experimental study. *Psychological Medicine*, *48*(13), 2223–2234. <https://doi.org/10.1017/S0033291717003713>

Young, D. A., Neylan, T. C., Chao, L. L., O’Donovan, A., Metzler, T. J., & Inslicht, S. S. (2019). Child abuse interacts with hippocampal and corpus callosum volume on psychophysiological response to startling auditory stimuli in a sample of veterans. *Journal of Psychiatric Research*, *111*, 16–23. <https://doi.org/10.1016/j.jpsychires.2019.01.011>

Young, D. A., Neylan, T. C., O’Donovan, A., Metzler, T., Richards, A., Ross, J. A., & Inslicht, S. S. (2018). The interaction of BDNF Val66Met, PTSD, and child abuse on psychophysiological reactivity and HPA axis function in a sample of Gulf War Veterans. *Journal of Affective Disorders*, *235*, 52–60. <https://doi.org/10.1016/j.jad.2018.04.004>
