# Supplementary material for: How adverse childhood experiences get under the skin: A systematic review, integration and methodological discussion on threat and reward learning mechanisms
Source: eLife. 2024 Jul 16;13:e92700. doi: 10.7554/eLife.92700 (PMC11251725; doi:10.7554/eLife.92700)
Supplement: Supplementary file 2. [file elife-92700-supp2.docx]

**Supplementary file 2
List of Questionnaires used in the included studies**

|  | **Full name** | **No. items** | **Subscales** | **Reference** | **Comments** |
| --- | --- | --- | --- | --- | --- |
| CECA.Q | Childhood Experiences of Care and Abuse Questionnaire | 36 | Mother / Father | personal communication with Antonia Bifulco & AKo, 06.11.2022 |  |
| CTQ-SF | Childhood Trauma Questionnaire - Short Form | 28 | physical abuse, emotional abuse, sexual abuse, physical neglect, emotional neglect | personal communication with Vanessa Freund & AKo,, 18.11.2022 |  |
| ETISR -SF | Early Trauma Inventory Self Report-Short Form | 29 | General Traumas, Physical Punishment, Emotional Abuse, Sexual Events | personal communication with Douglas Bremner & AKo, 14.11.2022, MAPI platform (online portal) |  |
| JVQ | Juvenile Victimization Questionnaire | 34 | Conventional Crime, Child Maltreatment,Peer and Sibling Victimization, Sexual Victimizations, Witnessing and Indirect Victimization | Finkelhor et al. (2005) |  |
| LEC-5 | Life Events Checklist for DSM-V | 17 | - | Weathers et al. (2013) |  |
| LSC-R | Life Stressor Checklist - Revised | 30 | - | personal communication with Terence Keane, Michelle Bovin & AKo, 16.11.2022 |  |
| Life events checklist | Life events checklist | 27 | - | Caspi et al. (1996) | modified version |
| THS | Trauma History Screen | 14 | - | Carlson et al. (2005) |  |
| UCLA-PTSD RI | UCLA Child/Adolescent PTSD Reaction Index for *DSM-5* | 15 | - | personal communication with Alan Steinberg & AKo, 14.11.2022 |  |
| VEX-R | Violence Exposure Scale for Children-Revised | 25 | - | personal communication with Ariana Shahinfar & AKo, 14.11.2022 |  |
| ACE-Q | Adverse Childhood Experiences Questionnaire | 19 | - | Felitti et al. (1998) |  |
| CLES | Coddington life events scale | 40 | *-* | personal communication with Karen Smith & AKo, 14.01.2023 | modified version |
| ELSQ | Early Life Stress Questionnaire | 19 | - | personal communication with Wojciech Dragan & AKo, 13.01.2023 |  |
| GHQ | Generalized Harassment Questionnaire | 21 | passive, verbal, physical, cyberbullying | personal communication with Kathleen Rospenda & JR, 25.01.2023 | school version |
| TAQ | Traumatic Antecedents Questionnaire | 40 | - | Luxenberg et al. (2001) |  |
| THQ | Traumatic History Questionnaire | 24 | Crime Related Events, General Disaster and Trauma, Physical and Sexual Experiences | Hooper et al. (2011) |  |
| PC-CTS | Conflict Tactics Scale | 30 | Nonviolent Discipline, Psychological Aggression, Physical Assault (minor /severe/ very severe),, Neglect | Straus et al. (1996) | parent questionnaire |
| TEI | Traumatic Events Inventory | 15 | *-* | personal communication with Jennifer Stevens & JR, 24.01.2023 | modified version |
| CAPI | Child Abuse Potential Inventory | 160 | - | Milner (1986) |  |
| MNBS -CR | The Multidimensional Neglectful Behavior Scale | 51 | Emotional, Cognitive, Supervision, Physical, Abandonment, Exposure to Conflict, Alcohol use, General appraisal | Straus et al. (1995) | modified version |
| HSQ | Home Screening Questionnaire | 34 | - | Frankenburg and Coops (1986) | modified version |
| Mac Arthur SSS | MacArthur Scale of subjective social status | 2 | - | Hoebel et al. (2015) |  |
| PEQ | Peer Experiences Questionnaire | 9 | Peer Victimization, Bullying | Vernberg et al. (1999) | customized by Giovazolias et al. (2010) |

**References Questionnaires**

Carlson, E., Palmieri, P., Smith, S., Kimerling, R., Ruzek, J., & Burling, T. (2005). *The
 Trauma History Screen (THS).* [Measurement instrument]. Available from
 <http://www.ptsd.va.gov>

Caspi, A., Moffitt, T. E., Thornton, A., Freedman, D., Amell, J. W., Harrington, H., Smeijers,
 J., & Silva, P. A. (1996). The life history calendar: A research and clinical assessment
 method for collecting retrospective event-history data. International Journal of
 Methods in Psychiatric Research, 6(2), 101–114. [https://doi.org/10.1002/(SICI)1234-
 988X(199607)6:2<101::AID-MPR156>3.3.CO;2-E](https://doi.org/10.1002/(SICI)1234-%20%09988X(199607)6:2%3c101::AID-MPR156%3e3.3.CO;2-E)

Felitti, V. J., Anda, R. F., Nordenberg, D., Williamson, D. F., Spitz, A. M., Edwards, V.,
 Koss, M. P., & Marks, J. S. (1998). Relationship of childhood abuse and household
 dysfunction to many of the leading causes of death in adults. The Adverse Childhood
 Experiences (ACE) Study. *American Journal of Preventive Medicine, 14(*4), 245–258.
 <https://doi.org/10.1016/s0749-3797(98)00017-8>

Finkelhor, D., Hamby, S. L., Ormrod, R., & Turner, H. (2005). The Juvenile Victimization
 Questionnaire: reliability, validity, and national norms. *Child abuse & neglect*, *29*(4),
 383-412.

Frankenburg, W. K., & Coons, C. E. (1986). Home Screening Questionnaire: its validity in
 assessing home environment. *The Journal of pediatrics*, *108*(4), 624-626.

Hoebel, J., Müters, S., Kuntz, B. *et al.* Messung des subjektiven sozialen Status in der
 Gesundheitsforschung mit einer deutschen Version der MacArthur Scale.
 *Bundesgesundheitsbl.* 58, 749–757 (2015). https://doi.org/10.1007/s00103-015-2166-x

Hooper, L. M., Stockton, P., Krupnick, J., & Green, B. L. (2011). The development, use, and
 psychometric properties of the Trauma History Questionnaire. *Journal of Loss and
 Trauma,* *16,* 258-283.

Luxenberg, T., Spinazzola, J., & Van der Kolk, B. A. (2001). Complex trauma and disorders
 of extreme stress (DESNOS) diagnosis, part one: Assessment. *Directions in
 psychiatry*, *21*(25), 373-392.

Milner, J. (1986). The Child Abuse Potential Inventory Manual, *Webster, NC: Psytec*.

Straus, M. A., Hamby, S. L., Boney-McCoy, S., & Sugarman, D. B. (1996). The Revised
 Conflict Tactics Scales (CTS2): Development and Preliminary Psychometric Data.
 *Journal of Family Issues, 17*(3), 283–316.
 <https://doi.org/10.1177/019251396017003001>

Straus, M. A., Kinard, E. M., & Williams, L. M. (1995). The multidimensional neglectful
 behavior scale, Form A: Adolescent and adult-recall version. *Durham, NH: University
 of New Hampshire: Family Research Laboratory. Available in: http://pubpages. unh.
 edu/~ mas2*.

Vernberg, E. M., Jacobs, A. K., & Hershberger, S. L. (1999). Peer victimization and attitudes
 about violence during early adolescence. *Journal of clinical child psychology*, *28*(3),
 386-395. <https://doi.org/10.1207/S15374424jccp280311>
 (modified version available from http://users.sch.gr/euadamop/wordpress/wp-
 content/uploads/2016/04/PEQ-questionnaire.pdf)

Weathers, F. W., Blake, D. D., Schnurr, P. P., Kaloupek, D. G., Marx, B. P., & Keane, T. M.
 (2013). *The Life Events Checklist for DSM-5 (LEC-5) – Standard.* [Measurement
 instrument]. Available from <https://www.ptsd.va.gov/>
