# Supplementary material for: How adverse childhood experiences get under the skin: A systematic review, integration and methodological discussion on threat and reward learning mechanisms
Source: eLife. 2024 Jul 16;13:e92700. doi: 10.7554/eLife.92700 (PMC11251725; doi:10.7554/eLife.92700)
Supplement: Supplementary file 3. [file elife-92700-supp3.docx]

| **No.** | **Author (year)** | **Sample information** | | | | | | | | | **Childhood adversity** | | | | | | | |
| --- | --- | --- | --- | --- | --- | --- | --- | --- | --- | --- | --- | --- | --- | --- | --- | --- | --- | --- |
|  |  | **N** | **N male/female** | **Age mean in years** | **Age range** | **Recruitment strategy** | **Exclusion criteria** | **Assessement of general or recent adversity** | **Handling of psychiatric disorders** | **Psychopathology screening** | **N exposed** | **N unexposed** | **Categorical or dimenstional analyses** | **Exposure severity considered** | **Adversity type considered or recruited**  **blue = adverse experience green = exposure to potentially adverse events** | **Adversity types explicitly not considered** | **Assessment instrument** | **Cut-off used** |
| 1 | Machlin (2019) same sample as Milojevich (2020) | 64 | 27/36 | 6.2 | 4 - 7 | oversampling for at risk and diversity: low SES families, racial or ethnic minorities, low education of caregiver, or parents met a clinical cut-off for concern in the CAPI | none | no | included | no | not applicable (dimensional scores used) | | dimensional | yes | neglect, cognitive stimulation, violence exposure, domestic violence, physical abuse |  | VEX-R, CTS-2, CAPI,  MNBS-CR (subset), HSQ (subset), MacArthur SSS | z-transformed sum- score across measures |
| 2 | Milojevich (2020) same sample as Machlin (2019) | 64 | 27/36 | 6.2 | 4 - 7 | oversampling for at risk and diversity: low SES families, racial or ethnic minorities, low education of caregiver, or parents met a clinical cut-off for concern in the CAPI | none | no | included | no | not applicable (dimensional scores used) | | dimensional | yes | neglect, cognitive stimulation, violence exposure, domestic violence, physical abuse |  | VEX-R, CTS-2, CAPI,  MNBS-CR (subset), HSQ (subset) | z-transformed sum- score across measures |
| 3 | Marusak (2021) | 44 | 22/22 | 8.8 | 6 - 11 | recruited from local healthcare providers | nonnative English speakers, history of brain injury with loss of consciousness or a neurological condition (e.g., epilepsy), OCD, psychotic disorder, or significant learning disorder | no | included | PTSD, anxiety disorders, depression | 20 | 24 | categorical | no | domestic violence, witness, bullying/peer victimization, victim, Illness/medically-related trauma |  | parent and/or child report, JVQ | criterion A trauma listed on the UCLA PTSD RI (DSM-IV) |
| 4 | Jovanovic (2022) | 62 | 33/29 | 9 | 9 | recruited from a longitudinal study of trauma exposure, community sample in Detroit, Michigan. | autism spectrum disorder, hearing loss as assessed by an audiometer, and cognitive disability via parent report | no | included | PTSD, anxiety disorders | not applicable (dimensional scores used) | | dimensional | no | domestic and community violence, injuries, natural disasters, and verbal abuse (experienced or witnessed) |  | TESI-C | number of events |
| 5 | Qiu (2022) | 72 | 36/36 | 10.6 | not provided | recruited at a primary school | not reported | no | not excplicitly reported | depression | 31 | 41 | categorical and dimensional | no | parent being away for work for at least 6 consecutive month per year |  | one question | parent being away for work for at least 6 consequetive month per year |
| 6 | France (2022) | 29 | 14/15 | 9.55 | 9 | recruited through an ongoing study of childhood trauma exposure conducted by the Detroit Trauma Project, in Detroit, Michigan. | hearing loss, neurological disorder, developmental impairment, or autism spectrum disorder | no | not excplicitly reported | anxiety disorders | not applicable (dimensional scores used) | | dimensional | no | domestic and community violence, injuries, natural disasters, and verbal abuse (experienced or witnessed) |  | TESI-C | number of events |
| 7 | Silvers (2016) | 89 | 34/55 | 12 | 7 - 16 | recruitment from a larger study (no details, no refrence); no information how healthy individuals or previously institutionalized children were recurited | not reported | no | not excplicitly reported | anxiety disorders | 46 | 43 | categorical | not applicable | institutionalization and adoption age 3-120month | anything that is not prevoius instituationalization in an orphanage and subsequent adoption | not applicable | not applicable |
| 8 | DeCross (2022) | 147 | 74/73 | 12.65 | 8 - 16 | recruited at schools, and adoption / food bannk / after school programs, parenting programs, shelters, preventing programs and general community | IQ < 80, pervasive developmental disorder, psychosis, mania, substance abuse, safety concerns | no | included | PTSD, anxiety disorders, depression, externalizing disorders | 77 | 70 | categorical | no | physical and sexual abuse, direct witnessing of domestic violence (i.e. towards a caregiver) |  | CTQ, CECA, UCLA PTSD- RI, JVQ, VEX-R | CTQ: Bernstein '97 |
| 9 | McLaughlin (2016) same sample as Jenness (2018) | 90 | 47/43 | 13.5 | 6 - 18 | oversampling for at risk: recruited from schools, medical clinics, and the general community, neighborhoods with high levels of violent crime, clinics that serve predominantly low-SES clients, agencies that work with families exposed to violence | none | no | included + robustness analyses | PTSD, anxiety disorders, depression, externalizing disorders | 35 | 55 | categorical | yes | physical and sexual abuse, witnessing domestic violence | accidents, injuries and witnessing community violence; other scales than physical and sexual were not considered | CTQ + CECA composite score | CTQ: Bernstein '97 |
| 10 | Jenness (2018) same sample as McLaughlin (2016) | 94 | 48/46 | 13.5 | 6 - 18 | oversampling for at risk: recruited from schools, medical clinics, and the general community, neighborhoods with high levels of violent crime, clinics that serve predominantly low-SES clients, agencies that work with families exposed to violence | none | no | not excplicitly reported | PTSD | 38 | 56 | categorical | no | (a) physical abuse, sexual abuse, or witnessing more than 2 incidents of domestic violence during the CECA interview or (b) scores on the CTQ physical and sexual abuse subscales that exceed a validated threshold | accidents, injuries and witnessing community violence; other scales than physical and sexual were not considered | CTQ + CECA composite score | criterion A trauma listed on the UCLA PTSD RI (DSM-IV),  CTQ: Bernstein '97 |
| 11 | Susman (2021) | 165 | 86/79 | 12.65 | 9 - 17 | recruited from neighborhoods with high levels of violent crime, clinics that served a predominantly low SES catchment area, and agencies that work with families who have been victims of violence | IQ < 80, pervasive developmental disorder, psychosis, mania, substance abuse, safety concerns | no | included | PTSD, anxiety disorders, depression, externalizing disorders | 86 | 79 | categorical | no | physical and sexual abuse, direct witnessing of domestic violence |  | CTQ, CECA, UCLA PTSD- RI, JVQ, VEX-R | CTQ: Bernstein '97 |

|  |  | **Sample information** | | | | | | | | | **Childhood adversity** | | | | | | | |
| --- | --- | --- | --- | --- | --- | --- | --- | --- | --- | --- | --- | --- | --- | --- | --- | --- | --- | --- |
| **No.** | **Author (year)** | **N** | **N male/female** | **Age mean in years** | **Age range** | **Recruitment strategy** | **Exclusion criteria** | **Assessement of general or recent adversity** | **Handling of psychiatric disorders** | **Psychopathology screening** | **N exposed** | **N unexposed** | **Categorical or dimenstional analyses** | **Exposure severity considered** | **Adversity type considered or recruited**  **blue = adverse experience green = exposure to potentially adverse events** | **Trauma types explicitly not considered as exposure** | **Assessment instrument** | **Cut-off used** |
| 12 | Wolitzky-Taylor (2022) | 127 | 44/83 | 17 | baseline:  - 17  startle protocol completed:  16 - 18 | high school juniors from Los Angeles and Chicago, oversampling for high neuroticism | current clinically significant Axis I psychiatric disorder at the baseline assessment | no | current diagnoses excluded | PTSD, anxiety disorders, depression, externalizing disorders | not applicable (dimensional scores used) | | dimensional | yes | separations from or loss of a caregiver, caregiver neglect, emotional abuse, physical abuse, witnessing violence, and sexual abuse and assault |  | CTI | sum of adversity severity scores for each domain of adversity |
| 13 | Kreutzer (2021) | 112 | 36/76 | 18.5 | 17 - 19 | recruited through adverstisements on social media and in the local community | serious medical condition, psychotropic medication use, deafness, pregnancy, lifetime and/or current alcohol or substance use disorder, and psychosis. | no | included | PTSD, anxiety disorders, depression | 57 | 55 | categorical | no | exposures to actual or threatened death, serious injury, or sexual violence; divided into interpersonal and non-interpersonal trauma |  | SCID-5 | criterion A trauma listed on the UCLA PTSD RI (DSM-V) |
| 14 | Lange (2018) | 113 | 22/91 | 21 | 16 - 25 | recuited via posters in schools, pulblic places and newspaper ads | **HC:** history of psychiatric diagnosis or treatment, or a current DSM-IV axis I disorder; subclinical group: current psychiatric treatment or a significant need for care; **both groups:** lefthandedness, alcohol and substance dependence, current use of psychotropic drugs, a history of neurological disease, severe head trauma, organic brain disease and MRI contraindications | no | included | PTSD, anxiety disorders, depression | 58 | 55 | categorical | yes | emotional abuse, physical abuse, sexual abuse, emotional neglect and physical neglect |  | CTQ | median split |
| 15 | Zoladz(2022) | 291 | 156/135 low CA: 133/87  high CA: 71/48 | 19.37 | > 18 | healthy undergraduate students | PTSD, skin diseases, history of syncope or vasovagal response to stress, heart condition/ cardiovascular issues, severe head trauma, current treatment with narcotics, beta-blockers, steroids; substance use disorder,hearing loss, regular nightshift worl, recreational drugs | no | life time diagnoses excluded | PTSD, anxiety disorders, depression | 71 | 220 | categorical (high vs. low exposure) | no | emotional abuse, physical abuse, sexual abuse, emotional neglect and physical neglect |  | CTQ | CTQ: Bernstein '98 |
| 16 | Pole (2007) | 90 | 76/24 | 28.5 | not reported | incoming police academiy cohort in San Francisco | no exclusion criteria, but controlled for potentially confounding medication and past major depression | no | current diagnoses excluded | PTSD, anxiety disorders, depression | 25 | 65 | categorical | yes | disaster, physical assault, serious illness / accident, serious abuse, serious neglect, sexual assault | events that were merely witnessed, happend to some else and were not associated with intense peritraumatic distress | LSC-R | 0 events or 1 and more |
| 17 | Scharfenort (2016) | 76 | 41/35 | 25 | not reported | recruited from a screening sample (mainly students) | current or prior psychiatric / neurological disorders | recent | life time diagnoses excluded | PTSD, anxiety disorders, depression | 35 | 41 | categorical and dimensional | yes | general exposure (no subtypes) |  | life events checklist | 1 events or 1 and more |
| 18 | Lis (2020)  overlapping sample with Thome (2018) | PTSD: 64;  HC: 30 | all female | app. 35 | 18 - 65 | PTSD patients were recruited from a longitudinal treatment study (before they started treatment), HC recruited through the database at the Department for Psychosomatic Medicine and Psychotherapy, CIMH Mannheim, and public advertisements | **PTSD:** lifetime diagnosis of schizophrenia or Bipolar I disorder, current substance dependence, a body mass index <16, or intake of the following psychotropic drugs: tricyclic antidepressants, neuroleptics, trazodon, benzodiazepines, anxiolytic drugs, as well as beta adrenergic blocking agents. suicide attempt within the last 2 months; **HC:** no axis I disorder or borderline, no life time psychotherapy experience | no | explicitly clinical sample | PTSD, anxiety disorders, depression | 64 | 30 | categorical | yes | physical and sexual abuse |  | CTQ, LEC-5 | CTQ: Bernstein '98 |
| 19 | Thome (2018) overlapping sample with Lis (2020) | PTSD: 30;  TC: 30 HC:  30 | 0/30 each | app. 31 | 18 - 65 | PTSD patients were recruited from a longitudinal treatment study (before they started treatment), TC & HC recruited through the database at the Department for Psychosomatic Medicine and Psychotherapy, CIMH Mannheim, and public advertisements | **PTSD:** lifetime diagnosis of schizophrenia or Bipolar I disorder, current substance dependence, a body mass index <16, or intake of the following psychotropic drugs: tricyclic antidepressants, neuroleptics, trazodon, benzodiazepines, anxiolytic drugs, as well as beta adrenergic blocking agents. suicide attempt within the last 2 months; HC: no axis I disorder or borderline, no life time psychotherapy experience, **TC & HC:** lifetime diagnosis of any axis - I or borderline personality disorder, the intake of psychotropic drugs or experiences of psychotherapeutic interventions | no | explicitly clinical sample | PTSD, anxiety disorders, depression | 60 | 30 | categorical | yes | physical and sexual abuse  emotional abuse, physical abuse, sexual abuse |  | CTQ, LEC-5 | CTQ: Bernstein '98 |
| 20 | Jovanovic (2009) | 60 | 32/28 | app. 45 | 18 - 63 | recruited from a larger study investgating genetic and environmental factors contributing to PTSD , primarily African- American, low SES, inner-city population | active psychosis and severe medical illness | no | included | PTSD, anxiety disorders, depression | 20 | 40 | categorical | yes |  |  | CTQ | CTQ: Bernstein '98 |
| 21 | Stout (2021) | 2593 | 2593/0 | 22.7 | not reported | recruited from First Marine Division infantry battalions preparing to deploy to either Iraq or Afghanistan | none | recent | included | PTSD, anxiety disorders, depression | 1312 | 1145 | categorical and dimensional | yes | emotional abuse, physical abuse, sexual abuse, emotional neglect and physical neglect |  | CTQ | CTQ: Bernstein '98 |

|  |  | **Sample information** | | | | | | | | | **Childhood adversity** | | | | | | | |
| --- | --- | --- | --- | --- | --- | --- | --- | --- | --- | --- | --- | --- | --- | --- | --- | --- | --- | --- |
| **No.** | **Author (year)** | **N** | **N male/female** | **Age mean in years** | **Age range** | **Recruitment strategy** | **Exclusion criteria** | **Assessement of general or recent adversity** | **Handling of psychiatric disorders** | **Psychopathology screening** | **N exposed** | **N unexposed** | **Categorical or dimenstional analyses** | **Exposure severity considered** | **Adversity type considered or recruited**  **blue = adverse experience green = exposure to potentially adverse events** | **Trauma types explicitly not considered as exposure** | **Assessment instrument** | **Cut-off used** |
| 22 | Huskey (2022) | 42 | 7/35 | 20.4 | 18 - 50 | recruited via social media and flyers  - resulting in mainly (psychology) students | cardiorespiratory and cardiovascular conditions, history of seizures, epilepsy, neurological conditions, or brain disorders, Hearing impairments | recent | not excplicitly reported | PTSD, anxiety disorders | 17 | 9 | categorical and dimensional | no | traumatic events that comprise Criterion A of the current PTSD diagnosis as defined by the DSM-V |  | THS | criterion A trauma listed on the UCLA PTSD RI (DSM-V) |
| 23 | Bremner (2005) | 19 | 0/19 | 37 | not mentioned | newspaper advertisement | major medical illness, substance abuse, medication, organic mental disorders or co-morbid psychotic disorders, retained metal, a history of head trauma, loss of consciousness, cerebral infectious disease, or dyslexia | no | life time diagnoses excluded | PTSD, anxiety disorders, depression | 8 | 11 | categorical | no | physical,emotional, and sexual abuse, as well as general traumatic events |  | ETI-SR | sum score |
| 24 | Kuehl (2020) | 118 | 54/64 | app. 35 | not mentioned | recruited through public potings and via affective disorder unit at the Charité Berlin | CNS relevant diseases, neurological diseases, severe somatic diseases, diabetes type 1 and 2, diseases of steroid hormones, hypertonia, tinnitus, hearing impairments, current infections, pregnancy as well as the intake of psychotropic medication. **HC:** current mental disroder; **Depressed patients:** schizophrenia, schizoaffective disorder, bipolar disorder,depr. with psychotic features, dementia, panic disorder, alcool or drug dependence | no | current diagnoses excluded | PTSD, anxiety disorders, depression, externalizing disorders | 50 | 68 | categorical | no | repeated physical or sexual abuse at least once a month over one year or more |  | CTQ, ETI | physical or sexual abuse at least once a month over one year or more until the age of 18 |
| 25 | Morrison (2022) | 110 | all female | no access to information | 18 - 45 | Blood plasma samples were selected from a biobank generated by a larger study of risk factors for PTSD - participants were recruited through waitingrooms of a large urban tertiary care center serving low income populations with high trauma load | not reported | no | included | PTSD, depression | 74 | 27 | categorical | no | sexual abuse or assault (and different age of exposure gruops) |  | TEI (subset) | sum score |
| 26 | Klingelhöfer-Jens (2023) | 1402 | 557/845 | exposed: 26.8, unexposed: 25.1 | 18 - 50 | recruited via online platforms within a Collaborative Research Center  on fear and anxiety (SFB-TRR58) at the Universities of Würzburg, Münster, and Hamburg | left-handedness, non-Caucasian descent, current or lifetime diagnosis of psychiatric and neurological disorders, intake of illegal drugs or psychoactive medication, excessive consumption of alcohol, nicotine or caffeine, pregnancy | recent and general | current + lifetime diagnoses excluded | German version of the Mini International Psychiatric Interview | 203 | 1199 | both | yes | emotional abuse, physical abuse, sexual abuse, emotional neglect and physical neglect |  | CTQ | CTQ: Bernstein '98,  Häuser 2011 |
|  |  | **Studies with a slightly different focus or with ACEs only included in higher order interaction** | | | | | | | | | | | | | | | | |
| 27 | Jovanovic (2020) | 63 | 34/29 | 10 | 8 - 13 | Mothers were recruited from an ongoing study of PTSD and trauma exposure. | diagnosis of autism spectrum disorder, bipolar disorder, psychotic disorder, or cognitive disability | no | included | PTSD | 36 | 21 | categorical (high vs. low exposure) | no | minor and severe violence victimization and witnessing violence at home, school, and neighborhood. |  | VEX-R | median split |
| 28 | Stenson (2021) | 78 | 38/40 | 10.2 (first visit) | 8 - 13 | recruited from a larger study of African-American primary caregivers and children from a low- income, urban population with high trauma exposure (J | autism spectrum disorders, bipolar or psychotic disorders, and cognitive disability | no | included | PTSD, anxiety disorders | not applicable (dimensional scores used) | | included as covariate | yes | minor and severe violence victimization and witnessing violence in the home, school, and neighborhood. |  | VEX-R, TESI | not mentioned |
| 29 | Radoman (2019) | 70 | 21/49 | 181.4 | 17-19 | recruited through flyers social media neaby a highschool an college campus (all were students) | major active medical or neurological illness, lifetime history of manic/psychotic  symptoms or active suicidal ideation, deafness, traumatic brain  injury, psychotropic medication use (past 4 months), lifetime history of  alcohol or substance use disorder, positive drug screen, and pregnancy. | no | partly excluded/partly included | PTSD, anxiety disorders, depression | not applicable (dimensional scores used) | | dimensional | yes | bullying only (past year, aged 16-18) | any events earlier than the last 12 month and anything except for bullying | GHQ | sum score (includes frequency and intensity) |
| 30 | Rowland (2022) | FPS paradigm: 285; fMRI: 95 | all female | mid 30ies (unclear for subsample) see supplement | 18-65 | recruited through waiting rooms of general medical clinic with a low income black population visiting typically | history of bipolar disorder, schizophrenia, other psychotic disorder,pregnancy, or illegal drug use (cocaine, marijuana, opiates, amphe-tamines, and methamphetamines) | general | included | PTSD | not applicable (dimensional scores used) | | included as covariate | yes | sexual abuse by an older teenager or adult  a) between age 0 and 13, b) between 14 and 17 or after the age of 17 (the latter would not qualify as childhood really) |  | TEI (subset), CTQ | sum score |
| 31 | Estrada (2020) | 164 | 114/50 | 40.24 | 18-75 | recruited through flyers in a high crime region | insufficient reading, IQ < 70; schizophrenia, bipolar disorder, or psychosis, history of auditory impairment, loss of consciousness >30 min, seizures | general | included + robustness analyses | PTSD, anxiety disorders, depression, externalizing disorders | not mentioned | not mentioned | included as covariate | yes | emotional abuse, physical abuse, sexual abuse, emotional neglect and physical neglect |  | CTQ, ETV | not mentioned |
| 32 | Morey (2015) | 67 | 51/16 | 42 | not mentione | veterans from a repository | major Axis I diagnosis (other than depression), contraindication to MRI, substance dependence, traumatic brain injury, and neurological disorders | no | included | PTSD, anxiety disorders, depression, externalizing disorders | not applicable (dimensional scores used) | | dimensional | yes | emotional abuse, physical abuse, sexual abuse, emotional neglect and physical neglect |  | CTQ | sum score |

|  |  | **Sample information** | | | | | | | | | **Childhood adversity** | | | | | | | |
| --- | --- | --- | --- | --- | --- | --- | --- | --- | --- | --- | --- | --- | --- | --- | --- | --- | --- | --- |
| **No.** | **Author (year)** | **N** | **N male/female** | **Age mean in years** | **Age range** | **Recruitment strategy** | **Exclusion criteria** | **Assessement of general or recent adversity** | **Handling of psychiatric disorders** | **Psychopathology screening** | **N exposed** | **N unexposed** | **Categorical or dimenstional analyses** | **Exposure severity considered** | **Adversity type considered or recruited**  **blue = adverse experience green = exposure to potentially adverse events** | **Trauma types explicitly not considered as exposure** | **Assessment instrument** | **Cut-off used** |
| 33 | Schellhaas (2022) | 64 | 5/59 | 32 | 19-60 | recuitment of individuals with diverse range of childhood maltreatment | acute and/or chronic physical diseases (e.g. cardiovascular, respiratory, or neurological diseases), psychotic disorders, use of psychotropic drugs (except selective serotonin and norepinephrine reuptake inhibitors [SSRIs and SNRIs]), and current (past 12 months) substance dependence and/or abuse | no | partly excluded/partly included | PTSD, anxiety disorders, depression, externalizing disorders | all | 0 | included as covariate | yes | emotional abuse, physical abuse, sexual abuse, emotional neglect and physical neglect: in a pre-screening, one item of every subscale was presented with an overall value of >=1 |  | CTQ | sum score |
| 34 | Young (2018) overlapping sample with Young 2019 | 226 |  | 44.8 | not reported | gulf war veteran, recruited from larger study | severe physical impairment or medical illness, current or lifetime history of psychosis or of suicidal or homicidal  ideation, and a history of neurological or systemic illness affecting  central nervous system functioning | general | included | PTSD | 70 | 156 | categorical | no | childhood physical or sexual abuse prior to the age of 16 | all that are not sexual or phyical abuse | THQ | none (likely single item yes sufficient) |
| 35 | Young (2019) overlapping sample with Young 2018 | 147 | 115/32 | 50 | not reported | gulf war veteran, recruited from larger study | severe physical impairment or medical illness, current or lifetime history of psychosis or of suicidal or homicidal  ideation, and a history of neurological or systemic illness affecting  central nervous system functioning | general | included | PTSD | 45 | 102 | categorical | no | childhood physical or sexual abuse prior to the age of 16 | all that are not sexual or phyical abuse | THQ | none (likely single item yes sufficient) |
| 36 | Hall (2022) | 131 | 97/34 | 25.7 | 18-65 | sample was taken from a larger study with HC and individualy with depression, anxiety or both | major active medical or neurological problem; lifetime history of mania or psychosis; current OCD; intellectual disability or pervasive developmental disorder; current psychiatric treatment; psychoactive medication (past 4 months); a history of traumatic brain injury; pregnancy | no | explicitly clinical sample | anxiety disorders, depression | 45 | 86 | categorical | no | emotional abuse, physical abuse, sexual abuse, emotional neglect and physical neglect |  | CTQ | CTQ: Bernstein '98 |
| 37 | Deslauriers (2018) | 714 | 714/0 | app. 22 | not mentioned | US Marines from infantry battalions at bases in Southern California that were doployed to Afghanistan | All ancestries other than European-American were excluded to reduce population stratification, PTSD diagnosis before dedployment | general | not excplicitly reported | PTSD, depression | 264 | 450 | categorical | no | emotional abuse, physical abuse, sexual abuse, emotional neglect and physical neglect |  | CTQ | CTQ: Bernstein '98 |
| 38 | Harnett (2019) | 198 | 98/100 | 20.73 | one school cohort | initially recruited from 5th grade classrooms in local public schools as part of a larger study | not reported | no | not excplicitly reported | no | not reported | not reported | categorical | no | within the 12 months before each assessment: victimized or witnessed: 1) threat of violence, 2) physical violence, and  3) threat or physical violence involving a weapon; Victimized: 4) violent injury requiring medical treatment; family income , neighborhood disadvantage | traumatic events that happened outside the three 1-year time windows of assessment | customized questions | average scores for witnessing and victimization for each timepoint. the average score of all four time points was used as an index of violence exposure in the present study |
