# Supplementary material for: How adverse childhood experiences get under the skin: A systematic review, integration and methodological discussion on threat and reward learning mechanisms
Source: eLife. 2024 Jul 16;13:e92700. doi: 10.7554/eLife.92700 (PMC11251725; doi:10.7554/eLife.92700)
Supplement: Supplementary file 4. [file elife-92700-supp4.docx]

|  |  | **Sample information** | | | | | | | |  | **Childhood adversity** | | | | | | | |
| --- | --- | --- | --- | --- | --- | --- | --- | --- | --- | --- | --- | --- | --- | --- | --- | --- | --- | --- |
| **No.** | **Author (year)** | **N** | **N male/female** | **Age mean in yrs** | **Age range** | **Recruitment strategy** | **Exclusion criteria** | **Assessement of general or recent adversity** | **Handling of psychiatric disorders** | **Psychopathology screening** | **N exposed** | **N unexposed** | **Categorical or dimenstional analyses** | **Exposure severity considered** | **Exposure type considered or recruited**  blue = adverse experience  green = exposure to potentially adverse events | **Trauma types explicitely not**  **considered as exposure** | **Assessment instrument** | **Cut-off used** |
| 1 | Birn (2017) | 54 | 26/28 | 20.5 | 19-23 | recruited from larger study, stress assessment at age 10 and 10 year follow-up, highest and lowest quintiles re-contacted | none | recent | not explicitly reported | depression | 29 | 25 | dimensional | yes | neglect, abuse, general life stress |  | YLSI | > 4.0 for early life stress, < 2.5 for controls on a 10- point scale |
| 2 | Bjork (2008) | 26 | 16/10 | 13.9 | 12-16 | Recruitment through parent undergoing treatment + community | Axis I disorders, probable fetal alcohol exposure | no | exclusion | Axis I disorders | 13 | 13 | categorical | no | Parental alcoholism |  | DSM-IV criteria for alcoholism (SSAGA assessment) | One or two parents with alcohol use problem |
| 3 | Blair (2022) | 142 | 91/51 | 16.4 | 14-18 | care facility for behavioral and mental health problems, community via flyers or social media | none | no | not explicitly reported | MDD, GAD, PTSD, ADHD, CD | 91 | 51 | dimensional | yes | emotional/physical neglect, emotional/sexual/physical abuse |  | CTQ | CTQ: Walker et al., 1999 |
| 4 | Boecker-Schlier (2016) | 168 | 71/97 | 24.5 | 25 | large epidemiological study, children born between 1986-1988; first borns; German-speaking parents | handicap, MRI contraindications, current psychopathology, psychotropic medication | no | not explicitly reported | psychiatric disorders incl. ADHD; YASR | not applicable (dimensional scores used) | | dimensional | yes | emotional, sexual abuse, neglect, general adversity |  | parent interview | Rutter&Quinton, 1977; childhood family adversity compound score over childhood period |
| 5 | Casement (2014) | 120 | 0/120 | NA | 16 | recruited from larger longitudinal study (Pittsburg Girls Study); screened at age 8 for high depressive symptoms + matched controls; invited back at age 16 | Eligible for scanning | no | included | depression | not applicable (dimensional scores used) |  | dimensional | yes | Low parental warmth; peer victimization |  | Items from parent-child rating scale; items from peer experiences scale |  |
| 6 | Cisler (2019) same sample as Letkiewicz (2022) | 60 | 0/60 | not provided | 11-17 | community sample, half exposed to assault that could be remembered, half not; balance in PTSD diagnoses in assaulted sample | controls: mental health disorders, trauma exposure, and psychiatric treatment; all: histories of psychotic symptoms, developmental disorders, neurocognitive disorders, MRI contraindications, pregnancy, history of traumatic brain injury, loss of consciousness greater than 10 min, and major medical disorders | no | included | PTSD, mood, anxiety disorders | 30 | 30 | categorical and dimensional | yes | physical, sexual assault |  | interview for assault, CTQ | 1 or more assaults, CTQ only used dimentionally |
| 7 | DelDonno (2019) | 50 | 11/39 | 27 | 18-55 | community sample: roughly half of participants have MDD | psychotic symptoms, bipolar disorder or mania, family history of psychosis, suicidal attempts in the past 6 months, chronic or serious medical conditioning, smoking, alcohol or substance abuse | no | included | MDD; comorbid anxiety, BISBAS | 50 | 0 | dimensional | yes | emotional, physical or sexual abuse, emotional or physical neglect |  | CTQ |  |
| 8 | Delgado (2022) | 227 | 115/112 | 75.28 months | 5-7 | Recruited from nine public schools in Uruguay | none | no | not explicitly reported | NA | 108 | 119 | categorical | no | Low SES |  | Socioeconomic level index (SLI), questionnaire |  |
| 9 | Dennison (2016) | 59 | 23/36 | 17 | 13-20 | recruited from larger community- based study | psychiatric medication, braces, claustrophobia, active substance dependence, pervasive developmental disorder, non-English speaking, safety concerns | no | included | depression, externalizing and internalizing disorders | 21 | 38 | categorical | no | neglect, emotional, sexual and physical abuse |  | CTQ + CECA | report during CECA interview or CTQ cut-off (Walker et al., 1999) |
| 10 | Dennison (2019) | 94 | 48/46 | 13.6 | 6-19 | community sample: schools, prevention programs, medical clinics, general community | MRI contraindication and younger than 7 years of age for MRI portion | no | included | depression | 38 | 56 | categorical | no | physical or sexual abuse, neglect, food insecurity |  | CTQ + CECA + 4 questions about food insecurity | abuse: reported in CECA interview or CTQ subscale threshold (Walker et al., 1999); neglect: CECA cutoff (Bifulco et al., 2005) |
| 11 | Dillon (2009) | 44 | 20/24 | 33.8 | not provided | maltreated recruited from other study; community sample for controls | both: left-handedness, history of neurological or medical conditions, MRI contraindications; controls only: psychopathology, psychotropic medication, | no | included | MDD, anxiety disorders, PTSD | 11 | 31 | categorical | yes | emotional, physical or sexual abuse |  | adult attachment interview, conflict tactics scale, traumatic stress schedule (TSS) | multimodel assessment of whether or not abuse was present or not |
| 12 | Eckstrand (2019) | 111 | 33/78 | 22 | 18-25 | counseling service for mental healthcare, community ads, participant registry | all: left handedness, not fluent in English; controls: present psychological distress, personal history of psychiatric illness | no | included | anhedonia, depression, anxiety | 50 | 61 | dimensional | yes | crime-related events, general disaster, unwanted physical/sexual experience |  | THQ | no, dimensional approach |
| 13 | Gerin (2017) | 37 | 11/26 | 13 | 10-15 | via social services department; via schools/advertisements for controls | presence of pervasive developmental disorder, neurological abnormalities, MRI contraindications, IQ below 70 | no | included | anxiety, depression, PTSD, conduct problem, hyperactivity | 18 | 19 | categorical | yes | neglect, emotional, sexual and physical abuse |  | adversity history and severity reported by social worker or adoptive parent | severity rated from 0-4 (Kaufmann et  al., 1994); subtype estimated based on  file information |
| 14 | Gonzalez (2016) | 83 | 42/41 | 24.4 | NA | Recruited from larger, longitudinal study (Virginia Institute for Development in Adulthood) | If participants couldn’t bring opposite sex partner to testing session (due to ongoing relationship study); MR safety criteria not met | no | not explicitly reported | no | NA | NA | dimensional | yes | Low neighborhood quality |  | Initially: Neighborhood Quality Questionnaire (NQQ), controlled for parental SES and current income | no, dimensional approach |
|  |  | **Sample information** | | | | | | | |  | **Childhood adversity** | | | | | | | |
| **No.** | **Author (year)** | **N** | **N male/female** | **Age mean in yrs** | **Age range** | **Recruitment strategy** | **Exclusion criteria** | **Assessement of general or recent adversity** | **Handling of psychiatric disorders** | **Psychopathology screening** | **N exposed** | **N unexposed** | **Categorical or dimenstional analyses** | **Exposure severity considered** | **Exposure type considered or recruited**  blue = adverse experience  green = exposure to potentially adverse events | **Trauma types explicitely not**  **considered as exposure** | **Assessment instrument** | **Cut-off used** |
| 15 | Hanson (2017) | 81 | 40/41 | 15 | 12-17 | exposed: collaboration with child protective services; CPS send letters to families with documented maltreatment | none | no | not explicitly reported | no | 41 | 40 | categorical | no | physical abuse |  | official records + PC-CTS | Shalev (2012); PC-  CTS score >20 categorized as abused; <10 as unexposed |
| 16 | Harms (2017) | 53 | 31/22 | 15 | 14-17 | abuse: child protective services; controls: locals ads | none | no | not explicitly reported | no | 31 | 22 | categorical | yes | physical abuse |  | official records + PC-CTS, YLSI | abuse according to records; controls: no abuse according to records and parent interview |
| 17 | Hendrikse (2022) | 114 | 38/76 | 43 | 18-70 | purposive sampling | current medical condition, structural brain abnormality, current psychiatric disorder, psychiatric medications, positive toxicology, incomplete primary schooling | no | not excplicitly reported | MINI | 57 | 57 | categorical | yes | emotional, physical or sexual abuse, emotional or physical neglect |  | CTQ | CTQ: Bernstein et al., 1998 |
| 18 | Kennedy (2021) | 77 | 38/39 | 15.1 | 12-17 | flyers in community | none | no | not excplicitly reported | anxiety, depression, social withdrawal, conflict with others, violation of social norms | not applicable (dimensional scores used) | dimensional | yes | early life adversity |  | YLSI | none |  |
| 19 | Kwarteng (2021) | 2679 | 1368/1311 | NA | 9-10 | Data from publicly available data from larger study (ABCD study, recruited at age 9-10); screened for parental substance use and matched controls | Data with null values and unusable fMRI data | no | not explicitly reported | none | 1373 | 1306 | categorical | no | Parental substance use |  | Clinical manual for the National Consortium on Alcohol and Neurodevelopment in Adolescence Study (NCANDA) | Biological parents had to report two or more problems with alcohol or other drugs |
| 20 | Letkiewicz (2022) same sample as Cisler (2019) | 60 | 0/60 | 15.3 | 11-17 | community sample, half exposed to assault that could be remembered, half not; balance in PTSD diagnoses in assaulted sample | controls: mental health disorders, trauma exposure, and psychiatric treatment; all: histories of psychotic symptoms, developmental disorders, neurocognitive disorders, MRI contraindications, pregnancy, history of traumatic brain injury, loss of consciousness greater than 10 min, and major medical disorders | no | included | PTSD, mood, anxiety disorders | 31 | 29 | categorical | yes | physical or sexual assault |  | CTQ | sexual abuse scores based on cut-off of > 13; Bernstein et al., 1998 |
| 21 | Lloyd (2022) | 145 | not provided | 38.9 | not provided | exposed: ads in charity and support groups for trauma survivors; controls: community (university, Prolific) | none | no | not excplicitly reported | no | 47 | 98 | categorical | yes | emotional, physical or sexual abuse, emotional or physical neglect, parental imprisonment |  | ACE | high if ≥ 4 ACEs |
| 22 | Martz (2022)  Sample 1 | 11360 | 5907/5453 | 9.93 | 9-10 | Data from publicly available data from larger study (ABCD study, recruited at age 9-10); screened for parental substance use and matched controls |  | no | not explicitly reported | none | 1447/223 | 9690 | categorical | yes | Parental alcoholism |  | Clinical manual for the National Consortium on Alcohol and Neurodevelopment in Adolescence Study (NCANDA) | One or two parents with alcohol use problem |
| 23 | Martz (2022)  Sample 2 | 112 | 68/44 | 10.54 | 7-15 | Recruited from ongoing study of families with parental alcoholism (MLS study) | Mother drinking during pregnancy or signs of fetal alcohol spectrum disorder, any neurological, acute, uncorrected or chronic medical illness; any current or recent (within six months) treatment with centrally active medications, including sedative hypnotics; and history of psychosis or schizophrenia in first-degree relatives. The presence of most Axis I psychiatric or developmental disorders was exclusionary | no | exclusion | Axis I disorders | 51/40 | 21 | categorical | yes | Parental alcoholism |  | Diagnostic Interview Schedule -Version 4 | One or both parents with a lifetime history of alcoholism |
| 24 | McCutcheon (2019) | 42 | 20/22 | 26.5 | 18-45 | online, leaflets, newspaper ads | no personal history of psychiatric illness, no family history of psychotic illness | no | not excplicitly reported | no | 20 | 22 | categorical | no | 2 of 3: urban living, migration, childhood adversity (physical, emotional or sexual abuse) |  | official information + self- report + CTQ | urban living from census, CTQ: Bernstein et al., 2003 |
|  |  |  |  |  |  |  |  |  |  |  |  |  |  |  |  |  |  |  |
|  |  | **Sample information** | | | | | | | |  | **Childhood adversity** | | | | | | | |
| **No.** | **Author (year)** | **N** | **N male/female** | **Age mean in yrs** | **Age range** | **Recruitment strategy** | **Exclusion criteria** | **Assessement of general or recent adversity** | **Handling of psychiatric disorders** | **Psychopathology screening** | **N exposed** | **N unexposed** | **Categorical or dimenstional analyses** | **Exposure severity considered** | **Exposure type considered or recruited**  blue = adverse experience  green = exposure to potentially adverse events | **Trauma types explicitely not**  **considered as exposure** | **Assessment instrument** | **Cut-off used** |
| 25  26 | Mehta (2010)  Morelli (2021) | 23  46 | 12/11  21/25 | 16  7.3 | not provided  6-8 | Romanian adoptees living close to London; controls: local schools  flyers, mailing list, oversampling for parents w depression | MRI contraindications  developmental or physical disability, non-fluent English, lifetime history of psychotic or bipolar disorder in parent | no  recent | not excplicitly reported  included + robustness analyses | quasi-autism, hyperactivity, cognitive impairment, disinhibited attachment  depression, anxiety | 12 | 11 | categorical  dimensional | no  yes | adoption (global deprivation)  low familiy income, low parental education, single parent household, parental depression, parental hostility, four addtitional events of early life stress |  | deprivation experience + self report  composite score: low family income, low parental education, single parent household, exposure to parental depression, parental hostility, min. 4 stressful life events from preschool age psychiatric | sum of scores |
|  |  |  |  |  |  |  |  |  |  |  | not applicable (dimensional scores  used) | |  |  |  |  |  |  |
| 27 | Morris (2015) | 204 | 103/101 | 12.3 | 8-19 | Children of participants from previous study with either childhood onset depression or control group | Participants with low rewards | recent | included | Depression, anxiety | 86 | 118 | categorical | no | Parental depression |  |  | Parent with history of childhood onset depression assessed in previous study |
| 28 | Mueller (2012) | 46 | 20/26 | 16 | not provided | larger ongoing study (Infant Caregiver Project), controls: local newspapers | all: IQ below 80, controls: adopted, medical or psychiatric problems, history of maltreatment | no | included | anxiety, depression, bipolar | 17 | 29 | categorical | no | adoption, neglect |  | deprivation experience + self report | 1 or more experience |
| 29 | Müller (2014) Sample 1 | 412 | NA | NA | 13-15 | Participants were part of a large longitudinal European multi-center study (IMAGEN); recruitment via high schools | Serious medical conditions (e.g. diabetes, rheumatologic disorders, neurological conditions, developmental conditions), previous head trauma with unconsciousness and MRI contra-indications as well as heavy maternal alcohol use during pregnancy (>14 bottles of beer or nine glasses of wine per week) were exclusion criteria. | no | not explicitly reported | Psychiatric disorders | 206 | 206 | dimensional | yes | Alcoholism in first or second degree relative |  | Diagnosis by medical doctor/psychologist or in treatment for it |  |
| 30 | Müller (2014) Sample 2 | 154 | NA | NA | 13-15 | Participants were part of a large longitudinal European multi-center study (IMAGEN); recruitment via high schools | Serious medical conditions (e.g. diabetes, rheumatologic disorders, neurological conditions, developmental conditions), previous head trauma with unconsciousness and MRI contra-indications as well as heavy maternal alcohol use during pregnancy (>14 bottles of beer or nine glasses of wine per week) were exclusion criteria | no | not explicitly reported | Psychiatric disorders | 77 | 77 | categorical | no | Parental alcoholism |  | Diagnosis by medical doctor/psychologist or in treatment for it |  |
| 31 | Mullins (2020) | 6396 | NA | NA | 9-10 | Data from publicly available data from larger study (ABCD study, recruited at age 9-10); screened for parental substance use and matched controls | Participants who didn’t complete the MID, had missing CBCL scores, were scanning on scanner not from Siemens or GE | no | included | Internalizing and externalizing symptoms |  |  | dimensional | yes | Neighborhood deprivation |  | Area deprivation index (ADI) via US census data |  |
| 32 | Patterson (2013) Exp1 | 73 | 14/59 | 19.8 | 18-23 | undergrads at UCLA | none | no | not explicitly reported | anxiety, depression | 36 | 37 | categorical | no | emotional/physical/sexual abuse, domestic violence, divorce, household substance abuse/mental illness/criminal |  | ACEQ |  |
| 33 | Patterson (2013) Exp2 | 212 | 50/162 | 20.2 | 18-39 | undergrads at UCLA oversampled for ELS | none | no | not explicitly reported | anxiety, depression | 126 | 86 | categorical | yes | emotional/physical/sexual abuse, domestic violence, divorce, household substance abuse/mental illness/criminal |  | ACEQ | 0, 1-2 or 3+  experience for no, moderate and high ELS |
| 34 | Pechtel (2013) | 49 | 0/49 | 29 | not provided | online, printed ads | left handedness, significant medical or neurological conditions, current mood disorder, current or past psychotic symptoms, somatoform disorders, personality disorders, lifetime substance dependence, substance abuse within past 6 months, seizures, antidepressant medication in the past 2 months | no | included | MDD | 31 | 18 | categorical | no | childhood sexual abuse (min 3 episodes, age 7-12) | other physical or emotional abuse | self report |  |
| 35 | Romens (2016) | 123 | 0/123 | NA | 16 | recruited from larger longitudinal study (Pittsburg Girls Study); screened at age 8 for high depressive symptoms + matched controls; invited back at age 16 | Eligible for scanning | no | included | depression | not applicable (dimensional scores used) |  | dimensional | yes | Low SES |  | Number of years of household receipt of public assistance |  |
| 36 | Sheridan (2018) | 136 | 67/69 | 12 | 11-14 | BEIP longitudinal study: recruited from child rearding institutions; controls: pediatric clinics, three groups: never institutionalized (NIG), foster care (FCG), prolonged institutional care (CAUG) | none | no | included | depression, social functioning | 48/43 | 47 | categorical/dime nsional | yes | institutional rearing to varying degrees |  | deprivation experience |  |

|  |  | **Sample information** | | | | | | | |  | **Childhood adversity** | | | | | | | |  |
| --- | --- | --- | --- | --- | --- | --- | --- | --- | --- | --- | --- | --- | --- | --- | --- | --- | --- | --- | --- |
| **No.** | **Author (year)** | **N** | **N male/female** | **Age mean in yrs** | **Age range** | **Recruitment strategy** | **Exclusion criteria** | **Assessement of general or recent adversity** | **Handling of psychiatric disorders** | **Psychopathology screening** | **N exposed** | **N unexposed** | **Categorical or dimenstional analyses** | **Exposure severity considered** | **Exposure type considered or recruited**  blue = adverse experience  green = exposure to potentially adverse events | **Trauma types explicitely not**  **considered as exposure** | **Assessment instrument** | **Cut-off used** |  |
| 37 | Smith (2022) | 72 | 29/43 | 8.4 | 8-9 | from Midwestern city | none | no | not explicitly reported | anxiety, depression | not applicable (dimensional scores used) | | dimensional | yes | perceived social isolation |  | CLEC | number of stressful events, perception of social isolation as additional measure |  |
| 38 | Weiland (2014) | 70 | 46/24 | 20.1 | 18-22 | Recruited from ongoing study of families with parental alcoholism (MLS study) | Mother drinking during pregnancy or signs of fetal alcohol spectrum disorder, any neurological, acute, uncorrected or chronic medical illness; any current or recent (within six months) treatment with centrally active medications, including sedative hypnotics; and history of psychosis or schizophrenia in first-degree relatives. The presence of most Axis I psychiatric or developmental disorders was exclusionary | no | exclusion | Axis I disorders | 49 | 21 | categorical | no | Parental alcoholism |  | DSM-IV criteria for alcoholism | One or both parents with a lifetime history of alcoholism |  |
| 39 | Weiss (2019) | 51 | 33/18 | 51.7 | not provided | addiction clinic: all participants have opiod addiction, in treatment for 3+ months | none | no | not explicitly reported | self harm, drug use | 32 | 19 | categorical | no | physical, verbal or sexual abuse or rape |  | self report |  |  |
| 40 | White (2022) | 172 | 59/113 | 13.94 | 12-15 | Recruitment from Chicago area | Not in 8^th^ grade, not English-speaking, not in good health, fMRI contraindications; history of serious medical illness or axis I psychiatric disorder, medication on previous 3 months, hospitalization in previous 12 months | no | exclusion | Axis I disorders | not applicable (dimensional scores used) |  | dimensional | yes | Low SES |  | MacArthur Network Sociodemographic Questionnaire; income-to-poverty ratio as main measure |  |  |
| 41 | Wilkinson (2021) | 129 | 53/76 | 37.6 | 25-65 | screening through online platform Prolific | not fluent in English, mild cognitive impairment or dementia, mental health disorder, Parkinson's | recent and general | included | social status, depression, anhedonia, stress | 64 | 65 | categorical | no | abuse, neglect |  | ELSQ | 3 or more adverse experiences |  |
| 42 | Wismer Fries (2017) | 52 | 27/25 | 6.3 | not provided | exposed: from Easter European orphanages; controls: always resided with their birth parents | developmental disorder, known or suspected fetal alcohol exposure or fetal alcohol syndrome | no | not explicitly reported | indiscriminate behaviour | 26 | 26 | categorical | no | caregiving neglect (adoption, foster care) |  | deprivation experience | CTQ: Bernstein et al., 1998 |  |
| 43 | Yang (2021) | 45 | 22/23 | 14.9 | 11-19 | group 1: treatment seeking for trauma related CBT; group 2: treatment seeking for anxiety/depression related CBT | MRI contraindications, major medical problems, problems understanding the procedure | no | included + robustness analyses | depression, anxiety | not applicable (dimensional scores used) | | dimensional | yes | emotional, physical or sexual abuse, emotional or physical neglect |  | CTQ |  |  |
| 44 | Yau (2012) | 40 | 24/16 | 20.3 | 18-22 | Recruited from ongoing study of families with parental alcoholism (MLS study) | Mother drinking during pregnancy or signs of fetal alcohol spectrum disorder, any neurological, acute, uncorrected, or chronic medical illness; any Axis I psychiatric or developmental disorders; any current or recent (within 6 months) treatment with centrally active medications; a history of psychosis or schizophrenia in first-degree relatives; and a positive urine drug screen on the day of the study | no | exclusion | Axis I disorders | 20 | | 20 | categorical | Parental alcoholism |  |  | DSM-IV criteria for alcoholism | One or both parents with a lifetime history of alcoholism |
