# Supplementary material for: How adverse childhood experiences get under the skin: A systematic review, integration and methodological discussion on threat and reward learning mechanisms
Source: eLife. 2024 Jul 16;13:e92700. doi: 10.7554/eLife.92700 (PMC11251725; doi:10.7554/eLife.92700)
Supplement: Supplementary file 5. [file elife-92700-supp5.docx]

| **No.** | **Author (year)** | **Open data** | **Paradigm information I** | | | **Paradigm information II** | | | | | | | | | **Outcome measures** | | | | | |
| --- | --- | --- | --- | --- | --- | --- | --- | --- | --- | --- | --- | --- | --- | --- | --- | --- | --- | --- | --- | --- |
|  |  |  | **Paradigm type** | **Experimenal phases** | **Instruction** | **Reinforcement rate %** | **Stimulus (CS) type** | **Stimulus (CS) duration** | **ITI duration in s** | **Number of trials per CS (acquistion)** | **Number of trials per CS (extinction)** | **Number of trials per CS and GS (generalization)** | **US type** | **US calibration** | **Psycho- physiological measures** | **Min. amplitude SCR in us** | **SCR non- responder** | **Ratings** | **Neuroimaging** | **Other measures** |
| 1 | Machlin (2019) same sample as Milojevich (2020) | no | Fear conditioning (block design) | pre-cond, acq, immediate ext | information not provided by authors | 80% | geometric | 1.5 | 0.5 | 40 | 40 | not applicable | tone | fixed level | SCR | 0.05 | excluded (N=3) | contingency (after each exp. phase) | none | none |
| 2 | Milojevich (2020) same sample as Machlin (2019) | no | Fear conditioning (block design) | pre-cond, acq, immediate ext | information not provided by authors | 80% | geometric | 1.5 | 0.6 | 40 | 40 | not applicable | tone | not reported | SCR | 0.05 | not reported by authors | contingency (after each exp. phase) | none | none |
| 3 | Marusak (2021) | no | Fear conditioning | acq, immediate ext, 24h-ext recall (included but not reported: re-ext, renewal, CS+ unextinguished) | information not provided by authors | 80% | VR avatar | 4 | 4-9 | 8 | 8 | not applicable | scream | fixed level | SCR | 0.02 | not reported by authors | fear, expectancy (intermittend) | fMRI (only during exctinction recall for a subsample; ROI: AI, dACC, exploratory: amygdala, hippocampus, vmPFC) | distance to CS (VR) |
| 4 | Jovanovic (2022) | no | Fear conditioning | hab, acq, immediate ext | information not provided by authors | 100% | geometric | 6 | 9-22 | 9 | 12 | not applicable | airblast | fixed level | startle | not applicable | not applicable | contingency (intermittend during extinction) | none | none |
| 5 | Qiu (2022) | upon request | Fear conditioning | acq, gen | threat contingencies not explicitly provided | 75% (acq),  50% (gen) | faces | 3 | 3+1 | 16 | not applicable | 6 | white noise | individually calibrated for being scary/annoying/  unpleasant (range 90-  110dB) | none | not applicable | not applicable | fear, valence, arousal, expectancy (after each exp. phase) | none | none |
| 6 | France (2022) | no | Fear conditioning | acq, immediate ext | information not provided by authors | 100% | geometric | 6.5 | 9-22 | 9 | 12 | not applicable | airblast | fixed level | startle | not applicable | not applicable | expectancy (after each exp. block and phase) | none (fMRI only in a memory face viewing paradigm) | none |
| 7 | Silvers (2016) | no | Fear conditioning (block design) | acq (included but not analyzed: CS+ reinforced trial blocks) | threat contingencies not explicitly provided | no information provided (likely different between participants due to escape component) | geometric | min. 1s (variation due to excape component) | no ITI between CSs | 8 times 37s blocks (3 reinforced CS+, 3 non-reinforce CS+, 2 CS-), trial number differed between participants as block lenght was fixed but due to | none (even though the non-reinfoced CS+ woul be extinction) | not applicable | tone | individually calibrated to be annoying but not painful (max. 65 dB) | none | not applicable | not applicable | none | fMRI (ROI: amygdala, hippocampus) | reaction time for escape, connectivity |
| 8 | DeCross (2022) | yes | Fear conditioning (block design) | acq (to do: bei den block designs kann man ja irgendie nicht unterscheiden was acq und ext ist...) | information not provided by authors | 80% | geometric | 1.5 | 500ms | 40 | not applicable | not applicable | tone | fixed level | none | not applicable | not applicable | none | fMRI (ROI: right amygdala (amygdala, insula, and dorsal ACC) and default mode network (hippocampus, PHG and vmPFC)) | none |
| 9 | McLaughlin (2016) same sample as Jenness (2018) | no | Fear conditioning | pre-cond, acq, immediate ext | some threat contingencies provided | 80% | items | 8 | 8-12 | 10 | 8 | not applicable | tone+ picture | fixed level | SCR | 0.02 | included + robustness check | fear, liking, unpleantness (after each experimental phase) | MRI (subsample) | none |
| 10 | Jenness (2018) same sample as McLaughlin (2016) | no | Fear conditioning | pre-cond, acq, immediate ext | some threat contingencies provided | 80% | items | 8 | 8-12 | 10 | 8 | not applicable | tone+ picture | fixed level | SCR,  respiratory sinus arrhythmia (ECG) | 0.02 | excluded + robustness check | none | none | none |

|  |  |  | **Paradigm information I** | | | **Paradigm information II** | | | | | | | | | **Outcome measures** | | | | | |
| --- | --- | --- | --- | --- | --- | --- | --- | --- | --- | --- | --- | --- | --- | --- | --- | --- | --- | --- | --- | --- |
| **No.** | **Author (year)** | **Open data** | **Paradigm type** | **Experimenal phases** | **Instruction** | **Reinforcement rate %** | **Stimulus (CS) type** | **Stimulus (CS) duration** | **ITI duration in s** | **Number of trials per CS (acquistion)** | **Number of trials per CS (extinction)** | **Number of trials per CS and GS (generalization)** | **US type** | **US calibration** | **Psycho- physiological measures** | **Min. amplitude SCR in us** | **SCR non- responder** | **Ratings** | **Neuroimaging** | **Other measures** |
| 11 | Susman (2021) | yes | Fear conditioning | pre-cond, acq, immediate ext | some threat contingencies provided | 80% | items | 8 | 8-12 | 10 | 8 | not applicable | tone+ picture | fixed level | SCR, vagal tone (ECG) | 0.02 | excluded (N=11, whereof N=8 from violence-exposed group) | none | none | none |
| 12 | Wolitzky-Taylor (2022) | no | NPU threat task | startle hab, predictable, unpredictable and safe condition | explicit contingency instructions | not applicable | NPU  instructions | 55 | 22 | not applicable | not applicable | not applicable | shock | fixed level | startle | not applicable | not applicable | none | none | none |
| 13 | Kreutzer (2021) | no | NPU threat task | startle hab, predictable, unpredictable and safe condition | explicit contingency instructions | 100% | NPU  instructions | 6 | 15-21 | not applicable | not applicable | not applicable | shock | individually calibrated to be annoying but not painful | startle | not applicable | not applicable | none | none | none |
| 14 | Lange (2018) | no | Fear conditioning | pre-cond, acq, gen | some threat contingencies provided | 66% (acq), 50%  (gen) | geometric | 4. 4 | either 2.2 or 4.4 | 12 | not applicable | 12 | shock | individually calibrated to be highly uncomfortable but not painful | none | not applicable | not applicable | fear, valence, arousal (after each phase), US expectancy (trial- by-trial, during) | fMRI (ROI: amygdala, vmPFC) | none |
| 15 | Zoladz(2022) | yes | Fear conditioning | startle hab, pre- cond, acq, 24h delayed gen | information not provided by authors | 100% (acq), 0%  (gen) | +:  geometric  treat of shock instruction | 7  CS- : 6.25 | 9-22 | 12 | not applicable | 3 | airblast | fixed level | startle | not applicable | not applicable | expectancy (trial- by-trial, during trial) | none | none |
| 16 | Pole (2007) | no | Threat of shock | startle hab, low, medium and high threat conditions | explicit threat of shock instructions | not applicable |  | 0.4 | 30-50 | 5 stimuli per threat- level, order of medium and high level counterbalanced | not applicable | not applicable | shock | fixed level | SCL, startle, heart rate (ECG) | not applicable | N=11 excluded due to lacking valid responses | fear, anxiety, danger, anger, stress, annoyance, helplessness, safety, pleasure, calmness, contentment (after | none | none |
| 17 | Scharfenort (2016) | no | Fear conditioning | pre-cond, acq, 24- delayd ext, reinstatement | threat contingencies not explicitly provided | 100% | geometric | 6-8, mean: 7 | 10-16, mean:  13 | 14 | 14 | not applicable | shock | individually calibrated to be painful but tolerable | SCR | 0.02 | N=1 (day 1) and N= 5 (day 2) excluded due to insufficient data quality | fear (after each exp. phase) | fMRI (ROIs; amygdala, vmPFC, hippocampus, anterior insula cortex (AI), ACC and  thalamus) | none |
| 18 | Lis (2020)  overlapping sample with Thome (2018) | no | Fear conditioning | startle hab, pre- cond, acq, gen | some threat contingencies provided | 75% (acq) 25%  (gen) | geometric | 8 | 1.5 - 4 | 12 | not applicable | CS+/CS-: 8; GSs: 4 | shock | individually calibrated to be highly uncomfortable but not painful | startle | not applicable | not applicable | risk (trial-by-trial, during trial) | none | reaction time (for ratings) |
| 19 | Thome (2018) overlapping sample with Lis (2020) | no | Fear conditioning | startle hab, pre- cond, acq, gen | some threat contingencies provided | 75% (acq) 25%  (gen) | geometric | 8 | 1.5 - 4 | 12 | not applicable | CS+/CS-: 8; GSs: 4 | shock | individually calibrated to be highly uncomfortable but not painful | startle | not applicable | not applicable | risk (trial-by-trial, during trial) | none | reaction time (for ratings) |
| 20 | Jovanovic (2009) | no | AX+BX- | startle hab, acq, inhibition test | some threat contingencies provided | 100% | geometric | 6 | 9-22 | 12 | not applicable | not applicable | airblast | fixed level | startle | not applicable | not applicable | risk, contingency (trial-by-trial, during trial) | none | none |

|  |  |  | **Paradigm information I** | | | **Paradigm information II** | | | | | | | | | **Outcome measures** | | | | | |
| --- | --- | --- | --- | --- | --- | --- | --- | --- | --- | --- | --- | --- | --- | --- | --- | --- | --- | --- | --- | --- |
| **No.** | **Author (year)** | **Open data** | **Paradigm type** | **Experimenal phases** | **Instruction** | **Reinforcement rate %** | **Stimulus (CS) type** | **Stimulus (CS) duration** | **ITI duration in s** | **Number of trials per CS (acquistion)** | **Number of trials per CS (extinction)** | **Number of trials per CS and GS (generalization)** | **US type** | **US calibration** | **Psycho- physiological measures** | **Min. amplitude SCR in us** | **SCR non- responder** | **Ratings** | **Neuroimaging** | **Other measures** |
| 21 | Stout (2021) | no | aversive anticipation | anticipation, image viewing | threat contingencies explicitly provided | 100% | geometric | 8 | 8s cue + 2 s picture | 10 | not applicable | not applicable | picture | fixed level | startle | not applicable | not applicable | none | none | none |
| 22 | Huskey (2022) | no | AX+BX- | startle hab, pre- cond, acq, test1, re- acquiston, test2, immedidate ext | some threat contingencies provided | 100% | geometric | 7 | not mentioned exactly,but probably 3 seconds | 12 | 7 | not applicable | airblast | fixed level | startle, heart rate variability (ECG) | not applicable | not applicable | expectancy (trial- by-trial, during trial) | none | none |
| 23 | Bremner (2005) | no | Fear conditioning | pre-cond,acq, immediate ext, 24h delayed unpredictable US phase, 24h delayed ext | some threat contingencies provided | 100% | geometric | 4 | 6 | 16 | 16 | not applicable | shock | individually calibrated to be annoying | SCR, heart rate | not reported by authors | not reported by authors | none | PET (ROI: amygdala) | none |
| 24 | Kuehl (2020) | no | Fear conditioning | pre-cond, startle hab, acq, immediate ext | information not provided by authors | 75% | geometric | 7.5 | 8.5 | 8 | 8 | not applicable | shock | individually calibrated to be clearlly aversive but not painful | SCR, startle | 0.01 | not mentioned | none | none | none |
| 25 | Morrison (2022) | no | Fear conditioning | acq | information not provided by authors | 100% | geometric | CS+: 6.5  CS- : 6 | 9-22 | 3 blocks with 4 trials each CS+, CS-, Noise alone | not applicable | not applicable | airblast | fixed level | SCR, startle | none | included | none | none | none |
| 26 | Klingelhöfer-Jens (2023) | no | Fear conditioning | acq, gen | not instructed threat contingencies | acq: 83.3%; gen:  50% | faces | 6 | 9-12 | 12 | not applicable | 12 | scream | fixed level | SCR | 0.02 | included + robustness check | arousal, valence, contingency, (intermittend) | none | none |
|  |  |  | **Studies with a slightly different focus or with ACEs only included in higher order interaction** | | | | | | | | | | | | | | | | | |
| 27 | Jovanovic (2020) | no | Fear conditioning | startle hab, acq | information not provided by authors | 100% | geometric | 6.5 | 9-22 | 12 | not applicable | not applicable | airblast | fixed level | startle | not applicable | not applicable | none | none | none |
| 28 | Stenson (2021) | no | Fear conditioning | pre-cond, acq | information not provided by authors | 100% | geometric | 6.5 | 9-22 | 9 | not applicable | not applicable | airblast | fixed level | startle | not applicable | not applicable | none | none | none |
| 29 | Radoman (2019) | no | NPU threat task | predictable, unpredictable and safe condition | explicit contingency instructions | not applicable | NPU  instructions | 4 | 15-21 | not applicable | not applicable | not applicable | shock | individually calibrated to be highly annoying but not painful | startle | not applicable | not applicable | none | none | none |
| 30 | Rowland (2022) | no | Fear conditioning | acq, immediate ext | some threat contingencies provided | 100% | geometric | 6 | 9-22  (randomized) | 4 | 8 | not applicable | airblast | fixed level | startle | not applicable | non-applicable | none | none (fMRI only in an emotinoal face viewing paradigm) | none |

|  |  |  | **Paradigm information I** | | | **Paradigm information II** | | | | | | | | | **Outcome measures** | | | | | |
| --- | --- | --- | --- | --- | --- | --- | --- | --- | --- | --- | --- | --- | --- | --- | --- | --- | --- | --- | --- | --- |
| **No.** | **Author (year)** | **Open data** | **Paradigm type** | **Experimenal phases** | **Instruction** | **Reinforcement rate %** | **Stimulus (CS) type** | **Stimulus (CS) duration** | **ITI duration in s** | **Number of trials per CS (acquistion)** | **Number of trials per CS (extinction)** | **Number of trials per CS and GS (generalization)** | **US type** | **US calibration** | **Psycho- physiological measures** | **Min. amplitude SCR in us** | **SCR non- responder** | **Ratings** | **Neuroimaging** | **Other measures** |
| 31 | Estrada (2020) | no | Fear conditioning | baseline phase, hab, acq, post-acq check | information not provided by authors | 100% | sounds | 1 | 10-20 | 6 | not applicable  not applicable | not applicable | startle probe | fixed level | SCR, startle | 0.01 | not excluced | none | none | none |
| 32 | Morey (2015) | no | Fear conditioning | pre-cond, acq, gen | information not provided by authors | acq + gen: 30%, | faces | pre-cond: 4s; acq: 6s, gen: 4 | pre-cond: 5-8  (mean 6.5);  acq: 10-15  (mean 12.5),  gen: 9-15  (mean 12) | 12 CS-, 18 CS+ |  | 8CS-, 12 CS+, 16  each GS | shock | individually calibrated to be highly annoying but not painful | none | not applicable | not applicable | expectancy (trial- by-trial, during trial) | fMRI (ROI: (amygdala, calcarine, IFG, insula, locus coeruleus, thalamus) | none |
| 33 | Schellhaas (2022) | yes | Threat of shock | theat and safety context during memory task after observational fear conditioning or threat instructions | explicit threat of shock instructions or contingencies acquired by observation | not applicable | treat of shock instruction after observational fear learning or threat instructions | not reported by authors (for video) | not reported by authors (for video) | not applicable | not applicable  not applicable | not applicable | shock (but fake electrode) | no calibration and no shock delievered (only threat of shock) | none | not applicable | not applicable | valence, danger, arousal | none | none |
| 34 | Young (2018) overlapping sample with Young 2019 | no | Threat of shock | low, medium and high threat conditions | explicit threat of shock instructions | not appplicable | treat of shock instruction | 4min per conditiong | 1 minute | 5 startle probes (for safe condition 10 but only last 5 analyzed) |  | not applicable | shock | fixed level | SCR, startle, heart rate | none | excluded (at least four (of five) valid responses with a trial for all three physiological measures required for inclusion) | none | MRI | none |
| 35 | Young (2019) overlapping sample with Young 2018 | no | Threat of shock | low, medium and high threat conditions | explicit threat of shock instructions | not appplicable | treat of shock instruction | 4min per conditiong | 1 minute | 5 startle probes (for safe condition 10 but only last 5 analyzed) | not applicable | not applicable | shock | fixed level | SCR, startle, heart rate | none | excluded (at least four (of five) valid responses with a trial for all three physiological measures required for inclusion) | none | MRI | none |
| 36 | Hall (2022) | no | NPU threat task | startle habituation, predictable, unpredictable and safe condition | explicit contingency instructions | 100% | NPU  instructions | 4 | 15-21 | not applicable | not applicable | not applicable | shock | individually calibrated to be highly annoying but not painful | startle | not applicable | not applicable | none | none | none |
| 37 | Deslauriers (2018) | no | Fear conditioning | acq, immediate ext | some threat contingencies provided | 75% | geometric | 6 | 8-13 | 8 | 16 | not applicable | airblast | fixed level | startle | not applicable | not applicable | none | none | none |
| 38 | Harnett (2019) | upon request | Fear conditioning with US alone trials | acq (only US responses), not reported: responding to CS+ and CS- | information not provided by authors | 100% | sounds | 10 | 18 | 24 | not applicable | not applicable | tone | fixed level | SCR | 0.05 | not mentioned | expectancy (trial- by-trial, during trial) | fMRI (ROIs: dlPFC,  dmPFC, vmPFC, hippocampus, amygala) | none |
