# Supplementary material for: How adverse childhood experiences get under the skin: A systematic review, integration and methodological discussion on threat and reward learning mechanisms
Source: eLife. 2024 Jul 16;13:e92700. doi: 10.7554/eLife.92700 (PMC11251725; doi:10.7554/eLife.92700)
Supplement: Supplementary file 6. [file elife-92700-supp6.docx]

|  |  |  | **Paradigm information I** | | | **Paradigm information II** | | | | | **Outcome measures** | | | | |
| --- | --- | --- | --- | --- | --- | --- | --- | --- | --- | --- | --- | --- | --- | --- | --- |
| **No.** | **Author (year)** | **Open data** | **Paradigm type** | **Paradigm name** | **Reward type** | **Reinforcement rates/reward values** | **Stimulus type** | **Total number of trials** | **Phases of interest** | **ITI duration in s** | **Outcome measure I** | **Outcome measure II** | **Outcome measure III** | **fMRI** | **other imaging measures** |
| 1 | Birn (2017) | no | probabilistic learning task | monetary incentive delay task | monetary | -5 to + $5 | geometric symbols | 90 | anticipation, feedback | 2-6 | no behavioral measures for MID task | commission errors |  | yes |  |
| 2 | Blair (2022) | No | probabilistic learning task | passive avoidance | monetary | 20 vs 80 % | shapes | 108 | learning | 1-4 | omission errors |  |  | yes |  |
| 3 | Bjork (2008) | no | probabilistic learning task | monetary incentive delay task | monetary | -5 to + $5 | geometric symbols | 108 | anticipation |  | Hit rate | Reaction time | Omission errors | yes |  |
| 4 | Boecker-Schlier (2016) | No | reward learning task | monetary incentive delay task | monetary or verbal | 60% | smiley | 100 | anticipation, reward delivery | 3-5 | reaction time | number of win trials |  | yes |  |
| 5 | Casement (2014) | No | probabilistic learning task | Reward-guessing task | monetary | Loss, no change, win | cards | 24 | anticipation | 9 |  |  |  | yes |  |
| 6 | Cisler (2019) | No | probabilistic learning task | three-arm bandit task | monetary | 20, 50 or 80 % | faces, houses | 90 | decision, anticipation, feedback | 1.5-3 | prediction error | learning rate | value expectations | yes |  |
| 7 | DelDonno (2019) | No | reward learning task | monetary incentive delay task | monetary | -5 to + $5 | visual | 100 | anticipation | 4 | total reward | accuracy |  | yes |  |
| 8 | Delgado (2022) | No | reward learning task | Children’s gambling task | points | -6 to 6 | cards | 60 | Learning/decision-making |  | Number of advantageous choices |  |  | no |  |
| 9 | Dennison (2016) | No | probabilistic learning task | monetary incentive delay task | monetary | -5 to + $5 | geometric symbols | 208 | response | 2-2.375 | reaction time |  |  | no (for reward) |  |
| 10 | Dennison (2019) | No | probabilistic learning task | monetary incentive delay task | monetary | 0-4 stars | cartoon faces | 132 | anticipation, response, feedback | 1-2 | reward earned | reaction time |  | no | white matter microstructure |
| 11 | Dillon (2009) | No | probabilistic learning task | monetary incentive delay task | monetary | -2.19 to + $2.34 | geometric symbols | 120 | anticipation | 3-7.5 | reaction time | affective ratings |  | yes |  |
|  |  |  | **Paradigm information I** | | | **Paradigm information II** | | | | | **Outcome measures** | | | | |
| **No.** | **Author (year)** | **Open data** | **Paradigm type** | **Paradigm name** | **Reward type** | **Reinforcement rates/reward values** | **Stimulus type** | **Total number of trials** | **Phases of interest** | **ITI duration in s** | **Outcome measure I** | **Outcome measure II** | **Outcome measure III** | **fMRI** | **other imaging measures** |
| 12 | Eckstrand (2019)  Gerin (2017)  Hanson (2017) | No | probabilistic learning task | card-guessing task | monetary | win, loss, mixed, neutral | cards | 96 | feedback | 2-6 | reward prediction error (RPE) |  |  | yes | connectivity |
| 13 |  | No | probabilistic learning task | passive avoidance | points | 70 % | animals | 56 | decision, feedback | 0-4 | omission errors | commission errors | total errors | yes |  |
| 14 |  | No | probabilistic learning task | probabilistic learning task | points | 20 vs 80 % and 30 vs 70% | everyday objects | 200 | NA | 1-6 | decision weights | variability of choice | reward expectation | no |  |
| 15 | Harms (2017) | No | probabilistic learning task | instrumental learning | points | 50 % | everyday objects | 96 | acquisition, reversal | 0.2 | accuracy | learning rate |  | yes |  |
| 16 | Gonzalez (2016) | No | reward learning task | monetary incentive delay task | monetary | -5 to + $5 | geometric symbols | 144 | anticipation, feedback | 2-2.5s | reaction time |  |  | yes |  |
| 17 | Hendrikse (2022) | No | reward learning task | monetary incentive delay task | monetary | 50% | emoticons | 60 | anticipation, feedback |  | reaction time |  |  | yes |  |
| 18 | Kennedy (2021) | No | reward learning task | probabilistic learning task | points | 70% or 80% | everyday objects | 200 | decision, feedback | 1-6 | prediction error | feedback sensitivity parameter |  | no | quantitative anisotropy |
| 19 | Kwarteng (2021) | Yes | probabilistic learning task | monetary incentive delay task | monetary | -5 to + $5 | geometric symbols | 100 | anticipation | 3.5-4.15 | no behavioral measures for MID task |  |  | yes |  |
| 20 | Letkiewicz (2022) | no | reward learning task | social reinforcement learning task | monetary | 20, 50 or 80 % | faces | 90 | (decision, anticipation,) feedback | 1.5-3 | prediction error | learning rate |  | yes |  |
| 21 | Lloyd (2022) | no | exploration-exploitation task | patch foraging task | monetary | variable | apples as tokens | NA | decision, harvest time |  | reaction time | time spend in poor and rich environments |  | no |  |
| 22 | Martz (2022) | no | probabilistic learning task | monetary incentive delay task | monetary | -5 to + $5 | geometric symbols | 100 | anticipation |  | no behavioral measures for MID task |  |  | yes |  |

|  |  |  | **Paradigm information I** | | | **Paradigm information II** | | | | | **Outcome measures** | | | | |
| --- | --- | --- | --- | --- | --- | --- | --- | --- | --- | --- | --- | --- | --- | --- | --- |
| **No.** | **Author (year)** | **Open data** | **Paradigm type** | **Paradigm name** | **Reward type** | **Reinforcement rates/reward values** | **Stimulus type** | **Total number of trials** | **Phases of interest** | **ITI duration in s** | **Outcome measure I** | **Outcome measure II** | **Outcome measure III** | **fMRI** | **other imaging measures** |
| 23 | McCutcheon (2019) | no | probabilistic learning task | salience attribution test | monetary | 50, 90 % | everyday objects | 128 | NA |  | probability ratings | reaction time |  | yes | connectivity |
| 24 | Mehta (2010) | no | probabilistic learning task | monetary incentive delay task | monetary | 0 to $2 | geometric symbols | 144 | anticipation, feedback | variable | accuracy | reaction time |  | yes |  |
| 25 | Morelli (2021) | no | reward learning task | monetary incentive delay task | monetary | 50 % | pinatas | 60 | anticipation, feedback | 2.5-5.5 | none |  |  | yes |  |
| 26 | Morris (2015) | no | Reward learning task | Signal detection task | monetary | 25 vs 75 % | smileys | 300 | learning |  | Response bias | discriminability | accuracy |  |  |
| 27 | Mueller (2012) | no | probabilistic learning task | monetary incentive saccade task | monetary | -1 to + $1 | asterisk | 144 | NA |  | accuracy of saccades | latency |  | no |  |
| 28 | Müller (2014) | No | probabilistic learning task | passive avoidance | monetary | -5 to + $5 | geometric symbols | 66 | anticipation | 3.5-4.15 | success | Reaction time | Errors | yes |  |
| 29 | Mullins (2020) | yes | reward learning task | monetary incentive delay task | monetary | -5 to + $5 | geometric symbols | 100 | anticipation | 3.5-4.15 | Reaction time |  |  | yes |  |
| 30 | Patterson (2013) Exp1 | no | instrumental learning task | instrumental learning task | monetary | 50% and 100% | sequence of button presses | variable | acquisition, extinction | 5 | number of trials | expectancy rating | correct responses | no |  |
| 31 | Patterson (2013) Exp2 | no | instrumental learning task | instrumental learning task | monetary | 50% and 100% | sequence of button presses | variable | acquisition, extinction | 2 | number of trials | correct responses |  | no |  |
| 32 | Pechtel (2013) | no | probabilistic learning task | stimulus selection task | verbal feedback | 20 vs 80 %, 30 vs 70%, 40  and 60% | Snodgrass images | 120 | learning, test | 0.35-0.55 | accuracy | reaction time |  | no |  |
| 33 | Romens (2016) | no | probabilistic learning task | Reward-guessing task | monetary | Loss, no change, win | cards | 24 | anticipation | 9 |  |  |  | yes |  |
| 34 | Sheridan (2018) | no | reward learning task | monetary incentive delay task | monetary | 0-4 stars | pinatas | 132 | anticipation, feedback |  | accuracy |  |  | no |  |

|  |  |  | **Paradigm information I** | | | **Paradigm information II** | | | | | **Outcome measures** | | | | |
| --- | --- | --- | --- | --- | --- | --- | --- | --- | --- | --- | --- | --- | --- | --- | --- |
| **No.** | **Author (year)** | **Open data** | **Paradigm type** | **Paradigm name** | **Reward type** | **Reinforcement rates/reward values** | **Stimulus type** | **Total number of trials** | **Phases of interest** | **ITI duration in s** | **Outcome measure I** | **Outcome measure II** | **Outcome measure III** | **fMRI** | **other imaging measures** |
| 35 | Smith (2022) | yes | Pavlovian conditioning | mixed appetitive and aversive conditioning | points/pictures (pos), sound/pictures (neg) | 20 vs 80 % | coloured shapes | 70 | acquisition, approach/avoid ance w CS | 2.5-5.5 | VAS ratings | learning rate (RT) | heart rate reactivity | no |  |
| 36 | Weiland (2014) | no | probabilistic learning task | monetary incentive delay task | monetary | 5 to + $5 | geometric symbols | 100 | anticipation |  | Succession rate | Amount won |  | yes |  |
| 37 | Weiss (2019) | no | probabilistic learning task | stimulus context reversal paradigm | points | NA | everyday objects | 40-64 | acquisition, retention, reversal |  | correct responses |  |  | no |  |
| 38 | White (2022) | no | probabilistic learning task | Passive avoidance task | monetary | -50 to + $50 | geometric symbols | 96 | Anticipation, Feedback | 0.5-2.5 | accuracy |  |  | yes |  |
| 39 | Wilkinson (2021) | yes | reward learning task | probabilistic reversal learning task (PRLT), probabilistic reward task (PRT) | monetary | 20, 80 % / 20, 60% | patterns/cartoon faces | unclear/300 |  |  | accuracy | learning rate | latency | no |  |
| 40 | Wismer Fries (2017) | no | probabilistic learning task | associative learning task | monetary | 33, 66, 100% | colours or shapes | 147 | learning | 1 | reaction time |  |  | no |  |
| 41 | Yang (2021) | no | reward learning task | monetary incentive delay task | monetary | 50 % | pinatas | 60 | anticipation, feedback | 2.5-5.5 | none |  |  | yes |  |
| 42 | Yau (2012) | no | probabilistic learning task | monetary incentive delay task | monetary | 5 to + $5 | geometric symbols | 100 | anticipation |  | Reaction time |  |  | yes |  |
