# Supplementary material for: How adverse childhood experiences get under the skin: A systematic review, integration and methodological discussion on threat and reward learning mechanisms
Source: eLife. 2024 Jul 16;13:e92700. doi: 10.7554/eLife.92700 (PMC11251725; doi:10.7554/eLife.92700)
Supplement: Supplementary file 7. [file elife-92700-supp7.docx]

**Supplementary file 7**

**Quality Assessment Tool**

*Adapted from* *Oltean, L. E., Șoflău, R., Miu, A. C., & Szentágotai-Tătar, A. (2023). Childhood adversity and impaired reward processing: A meta-analysis.*

**Sample**

1) Was the sample size adequate?

0 = < 30 per group
0,5 = < 50 per group
1 = 50 or more per group

2) Was the sample representative or not?

0 = No (e.g. female psychology students), also if not mentioned
 0,5 = Somewhat (e.g. recruited from university campus/community etc.)
 1 = Yes (e.g. both sexes, large age range, from community etc.)

3) Were the drop-outs described sufficiently?

0 = No description
 1 = Described adequately

4) Controlled for psychopathology?
0 = no, not explicitly reported
1 = yes, excluded or controlled for

**ACE assessment**

5) ACE types considered
 0 = only exposure (e.g. institutionalization, low SES) or only very specific/ few types
 considered, no control for other experiences
 1 = broad range of experiences considered

6) Quality of ACE measure(s):
0 = non validated “composite score” or customized questionnaires
1 = validated questionnaire(s) or interview or official records

7) Analysis of ACEs: Continuous or dichotomous score of ACEs? And if a dichotomous score was used, was there a control group?
 0 = dichotomous score and no control group

1 = dichotomous score WITH control group
 1 = continuous score
