# Supplementary material for: How adverse childhood experiences get under the skin: A systematic review, integration and methodological discussion on threat and reward learning mechanisms
Source: eLife. 2024 Jul 16;13:e92700. doi: 10.7554/eLife.92700 (PMC11251725; doi:10.7554/eLife.92700)
Supplement: Supplementary file 10. [file elife-92700-supp10.docx]

# Supplementary file 10

## Likelihood Ratio Tests

Here, we provide the results of the likelihood ratio tests we calculated for the patterns of results from the results section and discussion. We calculated the probability of the observed ratio of statistically significant results and null effects or results pointing in the opposite direction under the assumption that either the null or the alternative hypothesis is true (based on code provided by Lakens & Etz, 2017). The likelihood ratio is a comparison of how well the two hypotheses (in this case, H0 and H1) predict the observed data (given alpha =0.05 and a power of 0.8). It is important to note that the likelihood ratios are neither intended to serve as quantitative, meta-analytic metrics, nor do they provide any information on effect sizes of the studies or weigh the included studies by quality measures (as typically done in a meta-analysis). Likelihood ratios provide a heuristic estimation of whether the overall pattern of results across the included studies is rather in favor of the H0 or the H1. We believe that this provides additional information as it is highly unlikely that in a set of studies all (i.e., 100%) will yield significant effects even if an effect does exist. Hence, a heterogeneous pattern is more accepted than not expected and may still speak in favor of a specific effect (Lakens & Etz, 2017).

**Summary of Results - Threat learning**
For fear acquisition, 9 out of 21 studies reported blunted responses to the threat cue in individuals with a history of ACEs, yielding a likelihood ratio of 520.90. This indicates that the likelihood that the alternative hypothesis (i.e., there is an effect) is true is 521 times higher than the likelihood that the null hypothesis is true. In contrast, 3 out of 21 studies report enhanced responding to the threat cue in individuals with a history of ACEs which yields a likelihood ratio of 369,934,661.74 in favor of the null hypothesis (i.e., not in favor of an effect in this direction). For threat generalization, 5 out of 7 studies reported blunted responding to threat cues in individuals reporting ACEs compared to controls, yielding a likelihood ratio of 46,474.28 in favor of the alternative hypothesis (i.e., blunted responding).

**Summary of Results - Reward learning**
Of the 28 studies that reported behavioral measures of reward learning performance, 14 studies reported a significant reduction in reward learning or the valuation of rewarding outcomes. Fourteen studies reported no significant group differences or associations with ACEs in reward learning. This pattern of findings yields a likelihood ratio of 24,208,574.79 implying that - given the data - the alternative hypothesis (i.e., blunted reward learning) is 24,208,574.79 times more likely to be true than the null hypothesis.

**Discussion - No evidence from the literature for a link between specific ACE types and reward and/or threat learning**
**Threat-specific experiences and behavioral indices of threat learning:** Four out of 9 studies focusing on threat-specific experiences show blunted responding to threat-cues in individuals with a history of ACEs (Kuehl et al., 2020; Lis et al., 2020; McLaughlin, 2016; Thome et al., 2018) while three studies report null-findings (Jovanovic et al., 2009; Rowland et al., 2022; Stenson et al., 2021). This pattern of findings yields a likelihood ratio of 27.10, implying that - given the data - association between threatening experiences and blunted threat responding is 27.10 times more likely than the null hypothesis. Two out of these 9 studies report enhanced threat responding in individuals who report a history of ACEs (Marusak et al., 2021; Morrison et al., 2022), yielding a likelihood ratio of 213.12 in favor of the null hypothesis (i.e., not in favor of an effect in this direction).

Since there are only two studies focusing exclusively on **deprivation-related ACEs**, we could not calculate a likelihood ratio for this.

**Threat-specific experiences and behavioral indices of reward learning:** Four studies focusing on threat specific experiences (Hanson et al., 2017; Harms et al., 2018; Letkiewicz et al., 2022; Pechtel & Pizzagalli, 2013) report blunted reward learning, while only one study (Cisler et al., 2019) revealed a null result. Based on these findings, the likelihood ratio test suggests that an association between threat-only experiences and blunted responding is 13,797.05 times more likely than a null result.

**Deprivation-specific experiences and behavioral indices of reward learning:** Four studies (H. Delgado et al., 2022; Sheridan et al., 2018; White et al., 2022; Wismer Fries & Pollak, 2017) show reduced behavioral reward learning performance in the group exposed to deprivation experiences while three studies show no differences at the behavioral level (Gonzalez et al., 2016; Mehta et al., 2010; Smith & Pollak, 2022). Two additional studies did not provide behavioral measures of reward learning (Mullins et al., 2020; Romens et al., 2015). A likelihood ratio test provides further evidence that - given the data - an association between deprivation and blunted reward responding is 611.50 times more likely than a null result.

**References**

Cisler, J. M., Esbensen, K., Sellnow, K., Ross, M., Weaver, S., Sartin-Tarm, A., Herringa, R. J., & Kilts, C. D. (2019). Differential Roles of the Salience Network During Prediction Error Encoding and Facial Emotion Processing Among Female Adolescent Assault Victims. *Biological Psychiatry: Cognitive Neuroscience and Neuroimaging*, *4*(4), 371–380. <https://doi.org/10.1016/j.bpsc.2018.08.014>

Delgado, H., Aldecosea, C., Menéndez, Ñ., Rodríguez, R., Nin, V., Lipina, S., & Carboni, A. (2022). Socioeconomic status differences in children’s affective decision-making: The role of awareness in the Children’s Gambling Task. *Developmental Psychology*, *58*(9), 1716–1729. <https://doi.org/10.1037/dev0001382>

Deslauriers, J., Acheson, D. T., Maihofer, A. X., Nievergelt, C. M., Baker, D. G., Geyer, M. A., Risbrough, V. B., & Marine Resiliency Study Team. (2018). *COMT* Val158met polymorphism links to altered fear conditioning and extinction are modulated by PTSD and childhood trauma. *Depression and Anxiety*, *35*(1), 32–42. <https://doi.org/10.1002/da.22678>

Gonzalez, M. Z., Allen, J. P., & Coan, J. A. (2016). Lower neighborhood quality in adolescence predicts higher mesolimbic sensitivity to reward anticipation in adulthood. *Developmental Cognitive Neuroscience*, *22*, 48–57. <https://doi.org/10.1016/j.dcn.2016.10.003>

Hall, O. T., Phan, K. L., & Gorka, S. (2022). Childhood Adversity and the Association Between Stress Sensitivity and Problematic Alcohol Use in Adults. *Journal of Traumatic Stress*, *35*(1), 148–158. <https://doi.org/10.1002/jts.22709>

Hanson, J. L., Van Den Bos, W., Roeber, B. J., Rudolph, K. D., Davidson, R. J., & Pollak, S. D. (2017). Early adversity and learning: Implications for typical and atypical behavioral development. *Journal of Child Psychology and Psychiatry*, *58*(7), 770–778. <https://doi.org/10.1111/jcpp.12694>

Harms, M. B., Shannon Bowen, K. E., Hanson, J. L., & Pollak, S. D. (2018). Instrumental learning and cognitive flexibility processes are impaired in children exposed to early life stress. *Developmental Science*, *21*(4), e12596. <https://doi.org/10.1111/desc.12596>

Jenness, J. L., Miller, A. B., Rosen, M. L., & McLaughlin, K. A. (2019). Extinction Learning as a Potential Mechanism Linking High Vagal Tone with Lower PTSD Symptoms among Abused Youth. *Journal of Abnormal Child Psychology*, *47*(4), 659–670. <https://doi.org/10.1007/s10802-018-0464-0>

Jovanovic, T., Blanding, N. Q., Norrholm, S. D., Duncan, E., Bradley, B., & Ressler, K. J. (2009). Childhood abuse is associated with increased startle reactivity in adulthood. *Depression and Anxiety*, *26*(11), 1018–1026. <https://doi.org/10.1002/da.20599>

Jovanovic, T., Stenson, A. F., Thompson, N., Clifford, A., Compton, A., Minton, S., van Rooij, S. J. F., Stevens, J. S., Lori, A., Nugent, N., Gillespie, C. F., Bradley, B., & Ressler, K. J. (2020). Impact of ADCYAP1R1 genotype on longitudinal fear conditioning in children: Interaction with trauma and sex. *Neuropsychopharmacology*, *45*(10), 1603–1608. <https://doi.org/10.1038/s41386-020-0748-2>

Klingelhöfer-Jens, M., Hutterer, K., Schiele, M. A., Leehr, E., Schümann, D., Rosenkranz, K., Böhnlein, J., Repple, J., Deckert, J., Domschke, K., Dannlowski, U., Lueken, U., Reif, A., Romanos, M., Zwanzger, P., Pauli, P., Gamer, M., & Lonsdorf, T. B. (2023). Reduced discrimination between signals of danger and safety but not overgeneralization is linked to exposure to childhood adversity in healthy adults. *eLife*, *12*. <https://doi.org/10.7554/eLife.91425.1>

Kuehl, L. K., Deuter, C. E., Hellmann-Regen, J., Kaczmarczyk, M., Otte, C., & Wingenfeld, K. (2020). Enhanced noradrenergic activity by yohimbine and differential fear conditioning in patients with major depression with and without adverse childhood experiences. *Progress in Neuro-Psychopharmacology and Biological Psychiatry*, *96*, 109751. <https://doi.org/10.1016/j.pnpbp.2019.109751>

Lakens, D., & Etz, A. J. (2017). Too True to be Bad: When Sets of Studies With Significant and Nonsignificant Findings Are Probably True. *Social Psychological and Personality Science*, *8*(8), 875–881. <https://doi.org/10.1177/1948550617693058>

Letkiewicz, A. M., Cochran, A. L., & Cisler, J. M. (2022). Frontoparietal network activity during model-based reinforcement learning updates is reduced among adolescents with severe sexual abuse. *Journal of Psychiatric Research*, *145*, 256–262. <https://doi.org/10.1016/j.jpsychires.2020.10.046>

Lis, S., Thome, J., Kleindienst, N., Mueller-Engelmann, M., Steil, R., Priebe, K., Schmahl, C., Hermans, D., & Bohus, M. (2020). Generalization of fear in post-traumatic stress disorder. *Psychophysiology*, *57*(1). <https://doi.org/10.1111/psyp.13422>

Machlin, L., Miller, A. B., Snyder, J., McLaughlin, K. A., & Sheridan, M. A. (2019). Differential Associations of Deprivation and Threat With Cognitive Control and Fear Conditioning in Early Childhood. *Frontiers in Behavioral Neuroscience*, *13*, 80. <https://doi.org/10.3389/fnbeh.2019.00080>

Marusak, H. A., Hehr, A., Bhogal, A., Peters, C., Iadipaolo, A., & Rabinak, C. A. (2021). Alterations in fear extinction neural circuitry and fear-related behavior linked to trauma exposure in children. *Behavioural Brain Research*, *398*, 112958. <https://doi.org/10.1016/j.bbr.2020.112958>

McLaughlin, K. A. (2016). Future Directions in Childhood Adversity and Youth Psychopathology. *Journal of Clinical Child & Adolescent Psychology*, *45*(3), 361–382. <https://doi.org/10.1080/15374416.2015.1110823>

McLaughlin, K. A., Sheridan, M. A., Gold, A. L., Duys, A., Lambert, H. K., Peverill, M., Heleniak, C., Shechner, T., Wojcieszak, Z., & Pine, D. S. (2016). Maltreatment Exposure, Brain Structure, and Fear Conditioning in Children and Adolescents. *Neuropsychopharmacology : Official Publication of the American College of Neuropsychopharmacology*, *41*(8), 1956–1964. <https://doi.org/10.1038/npp.2015.365>

Mehta, M. A., Gore-Langton, E., Golembo, N., Colvert, E., Williams, S. C. R., & Sonuga-Barke, E. (2010). Hyporesponsive Reward Anticipation in the Basal Ganglia following Severe Institutional Deprivation Early in Life. *Journal of Cognitive Neuroscience*, *22*(10), 2316–2325. <https://doi.org/10.1162/jocn.2009.21394>

Milojevich, H. M., Machlin, L., & Sheridan, M. A. (2020). Early adversity and children’s emotion regulation: Differential roles of parent emotion regulation and adversity exposure. *Development and Psychopathology*, *32*(5), 1788–1798. <https://doi.org/10.1017/S0954579420001273>

Morey, R. A., Dunsmoor, J. E., Haswell, C. C., Brown, V. M., Vora, A., Weiner, J., Stjepanovic, D., Wagner, H. R., VA Mid-Atlantic MIRECC Workgroup, & LaBar, K. S. (2015). Fear learning circuitry is biased toward generalization of fear associations in posttraumatic stress disorder. *Translational Psychiatry*, *5*(12), e700. <https://doi.org/10.1038/tp.2015.196>

Morrison, K. E., Stenson, A. F., Marx-Rattner, R., Carter, S., Michopoulos, V., Gillespie, C. F., Powers, A., Huang, W., Kane, M. A., Jovanovic, T., & Bale, T. L. (2022). Developmental Timing of Trauma in Women Predicts Unique Extracellular Vesicle Proteome Signatures. *Biological Psychiatry*, *91*(3), 273–282. <https://doi.org/10.1016/j.biopsych.2021.08.003>

Mullins, T. S., Campbell, E. M., & Hogeveen, J. (2020). Neighborhood Deprivation Shapes Motivational-Neurocircuit Recruitment in Children. *Psychological Science*, *31*(7), 881–889. <https://doi.org/10.1177/0956797620929299>

Oltean, L.-E., Soflău, R., Miu, A. C., & Szentágotai-Tătar, A. (2022). Childhood adversity and impaired reward processing: A meta-analysis. *Child Abuse & Neglect*, 105596. <https://doi.org/10.1016/j.chiabu.2022.105596>

Pechtel, P., & Pizzagalli, D. A. (2013). Disrupted Reinforcement Learning and Maladaptive Behavior in Women With a History of Childhood Sexual Abuse: A High-Density Event-Related Potential Study. *JAMA Psychiatry*, *70*(5), 499. <https://doi.org/10.1001/jamapsychiatry.2013.728>

Radoman, M., Akinbo, F. D., Rospenda, K. M., & Gorka, S. M. (2019). The impact of startle reactivity to unpredictable threat on the relation between bullying victimization and internalizing psychopathology. *Journal of Psychiatric Research*, *119*, 7–13. <https://doi.org/10.1016/j.jpsychires.2019.09.004>

Romens, S. E., Casement, M. D., McAloon, R., Keenan, K., Hipwell, A. E., Guyer, A. E., & Forbes, E. E. (2015). Adolescent girls’ neural response to reward mediates the relation between childhood financial disadvantage and depression. *Journal of Child Psychology and Psychiatry*, *56*(11), 1177–1184. <https://doi.org/10.1111/jcpp.12410>

Rowland, G. E., Mekawi, Y., Michopoulos, V., Powers, A., Fani, N., Bradley, B., Ressler, K. J., Jovanovic, T., & Stevens, J. S. (2022). Distinctive impacts of sexual trauma versus non-sexual trauma on PTSD profiles in highly trauma-exposed, Black women. *Journal of Affective Disorders*, *317*, 329–338. <https://doi.org/10.1016/j.jad.2022.08.099>

Schellhaas, S., Schmahl, C., & Bublatzky, F. (2022). Social threat and safety learning in individuals with adverse childhood experiences: Electrocortical evidence on face processing, recognition, and working memory. *European Journal of Psychotraumatology*, *13*(2), 2135195. <https://doi.org/10.1080/20008066.2022.2135195>

Sheridan, M. A., McLaughlin, K. A., Winter, W., Fox, N., Zeanah, C., & Nelson, C. A. (2018). Early deprivation disruption of associative learning is a developmental pathway to depression and social problems. *Nature Communications*, *9*(1), 2216. <https://doi.org/10.1038/s41467-018-04381-8>

Smith, K. E., & Pollak, S. D. (2022). Early life stress and perceived social isolation influence how children use value information to guide behavior. *Child Development*, *93*(3), 804–814. <https://doi.org/10.1111/cdev.13727>

Stenson, A. F., van Rooij, S. J. H., Carter, S. E., Powers, A., & Jovanovic, T. (2021). A legacy of fear: Physiological evidence for intergenerational effects of trauma exposure on fear and safety signal learning among African Americans. *Behavioural Brain Research*, *402*, 113017. <https://doi.org/10.1016/j.bbr.2020.113017>

Thome, J., Hauschild, S., Koppe, G., Liebke, L., Rausch, S., Herzog, J. I., Müller-Engelmann, M., Steil, R., Priebe, K., Hermans, D., Schmahl, C., Bohus, M., & Lis, S. (2018). Generalisation of fear in PTSD related to prolonged childhood maltreatment: An experimental study. *Psychological Medicine*, *48*(13), 2223–2234. <https://doi.org/10.1017/S0033291717003713>

White, S. F., Nusslock, R., & Miller, G. E. (2022). Low Socioeconomic Status Is Associated with a Greater Neural Response to Both Rewards and Losses. *Journal of Cognitive Neuroscience*, *34*(10), 1939–1951. <https://doi.org/10.1162/jocn_a_01821>

Wismer Fries, A. B., & Pollak, S. D. (2017). The role of learning in social development: Illustrations from neglected children. *Developmental Science*, *20*(2), e12431. <https://doi.org/10.1111/desc.12431>

Young, D. A., Neylan, T. C., Chao, L. L., O’Donovan, A., Metzler, T. J., & Inslicht, S. S. (2019). Child abuse interacts with hippocampal and corpus callosum volume on psychophysiological response to startling auditory stimuli in a sample of veterans. *Journal of Psychiatric Research*, *111*, 16–23. <https://doi.org/10.1016/j.jpsychires.2019.01.011>

Young, D. A., Neylan, T. C., O’Donovan, A., Metzler, T., Richards, A., Ross, J. A., & Inslicht, S. S. (2018). The interaction of BDNF Val66Met, PTSD, and child abuse on psychophysiological reactivity and HPA axis function in a sample of Gulf War Veterans. *Journal of Affective Disorders*, *235*, 52–60. <https://doi.org/10.1016/j.jad.2018.04.004>
